# Supplementary figures and images for: DDK/Hsk1 phosphorylates and targets fission yeast histone deacetylase Hst4 for degradation to stabilize stalled DNA replication forks
Source: eLife. 2021 Oct 5;10:e70787. doi: 10.7554/eLife.70787 (PMC8565929; doi:10.7554/eLife.70787)

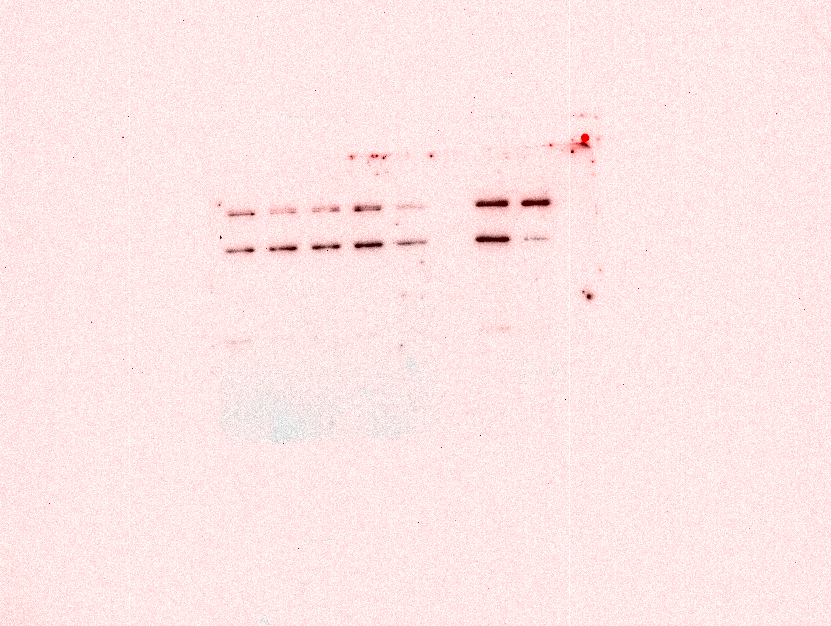

Supplement: Figure 1—source data 1. [file elife-70787-fig1-data1.zip › Figure 1/Figure 1A Hst4.png]

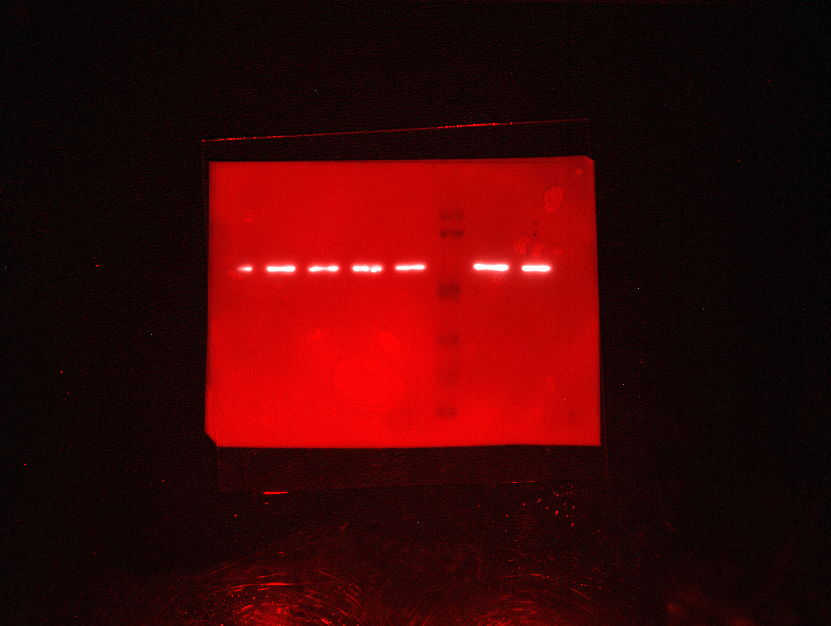

Supplement: Figure 1—source data 1. [file elife-70787-fig1-data1.zip › Figure 1/Figure 1A Tubulin.png]

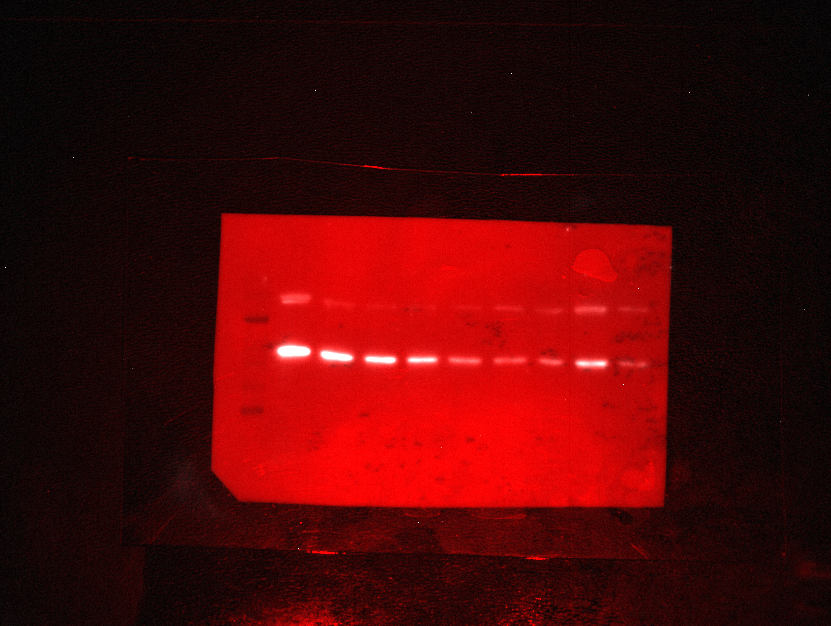

Supplement: Figure 1—source data 1. [file elife-70787-fig1-data1.zip › Figure 1/Figure 1B Hst4.png]

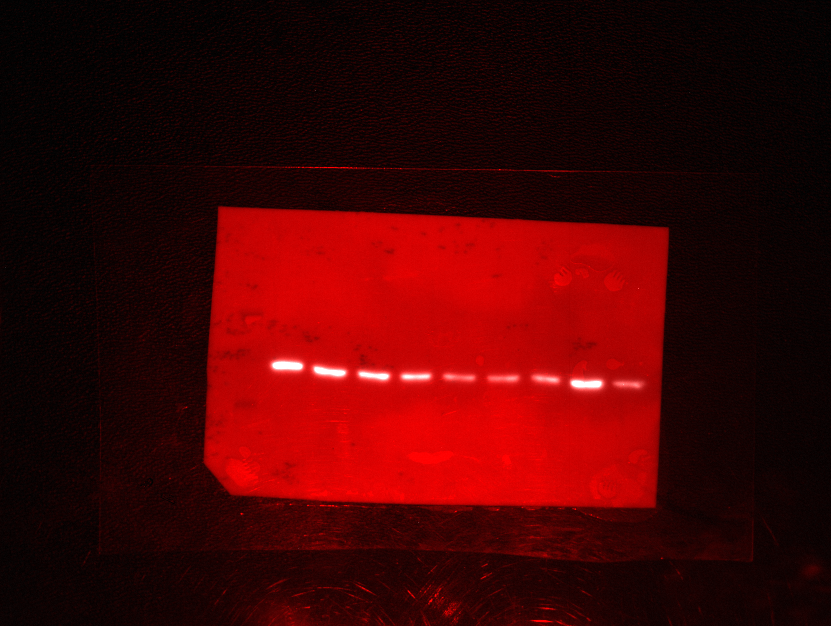

Supplement: Figure 1—source data 1. [file elife-70787-fig1-data1.zip › Figure 1/Figure 1B Tubulin.png]

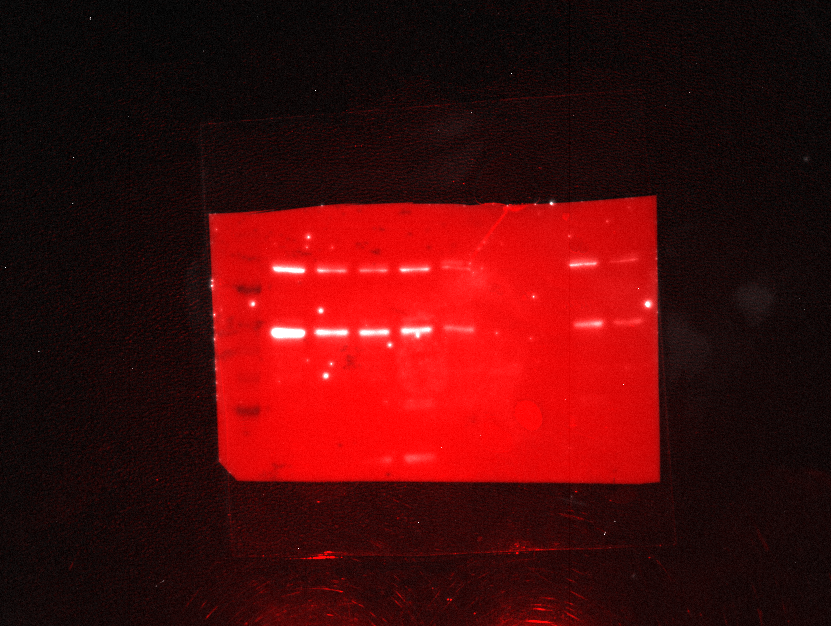

Supplement: Figure 1—source data 1. [file elife-70787-fig1-data1.zip › Figure 1/Figure 1C Hst4.png]

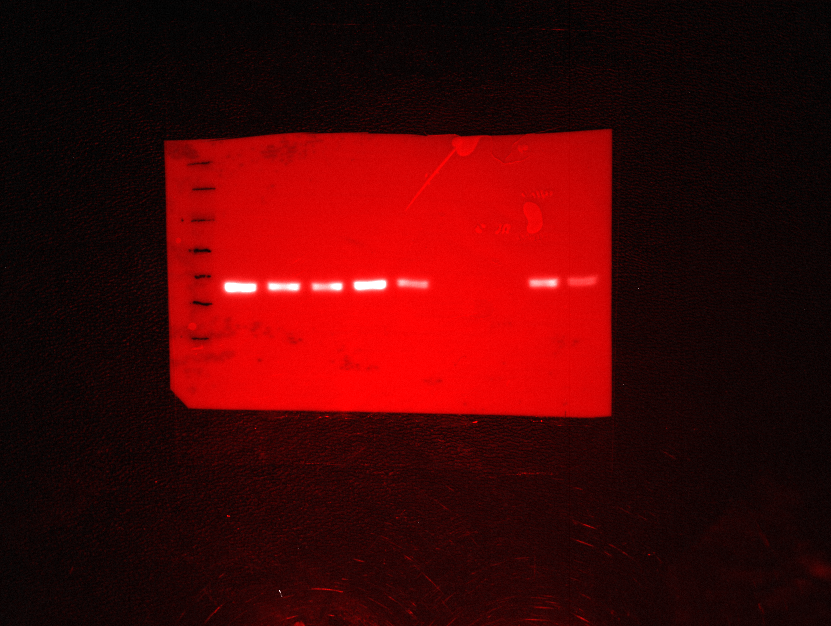

Supplement: Figure 1—source data 1. [file elife-70787-fig1-data1.zip › Figure 1/Figure 1C Tubulin.png]

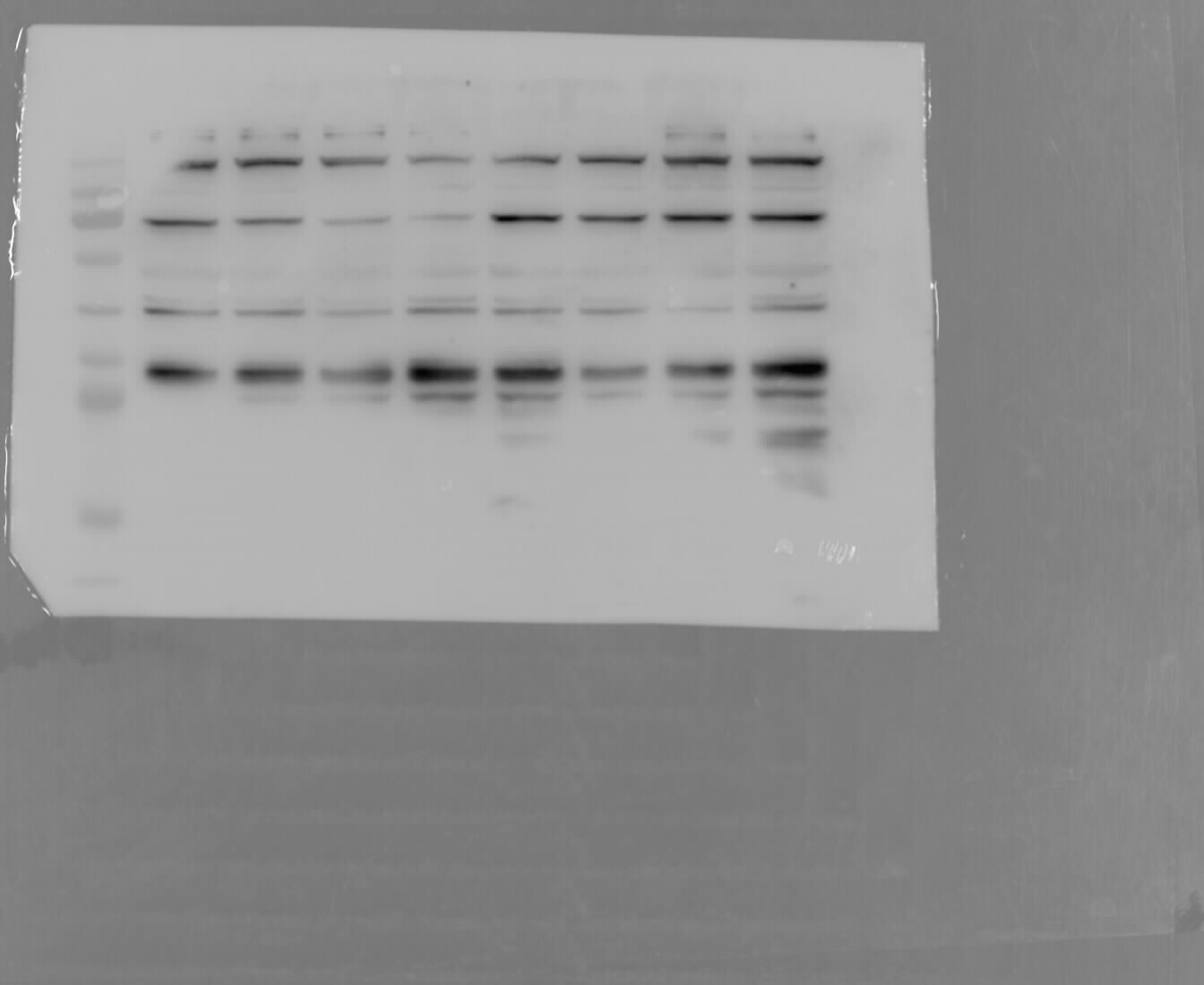

Supplement: Figure 1—source data 1. [file elife-70787-fig1-data1.zip › Figure 1/Figure 1D Hst4.JPG]

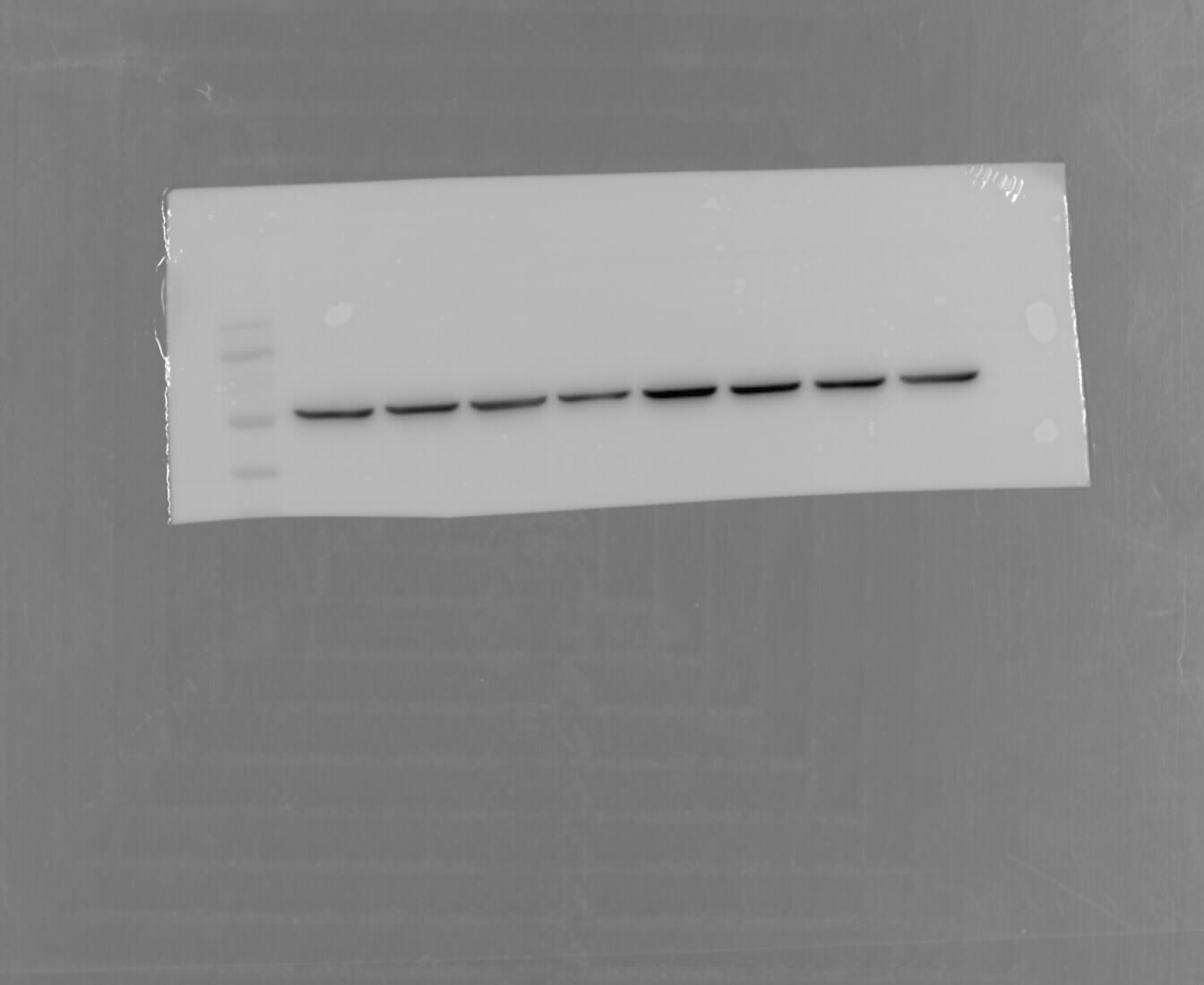

Supplement: Figure 1—source data 1. [file elife-70787-fig1-data1.zip › Figure 1/Figure 1D Tubulin.JPG]

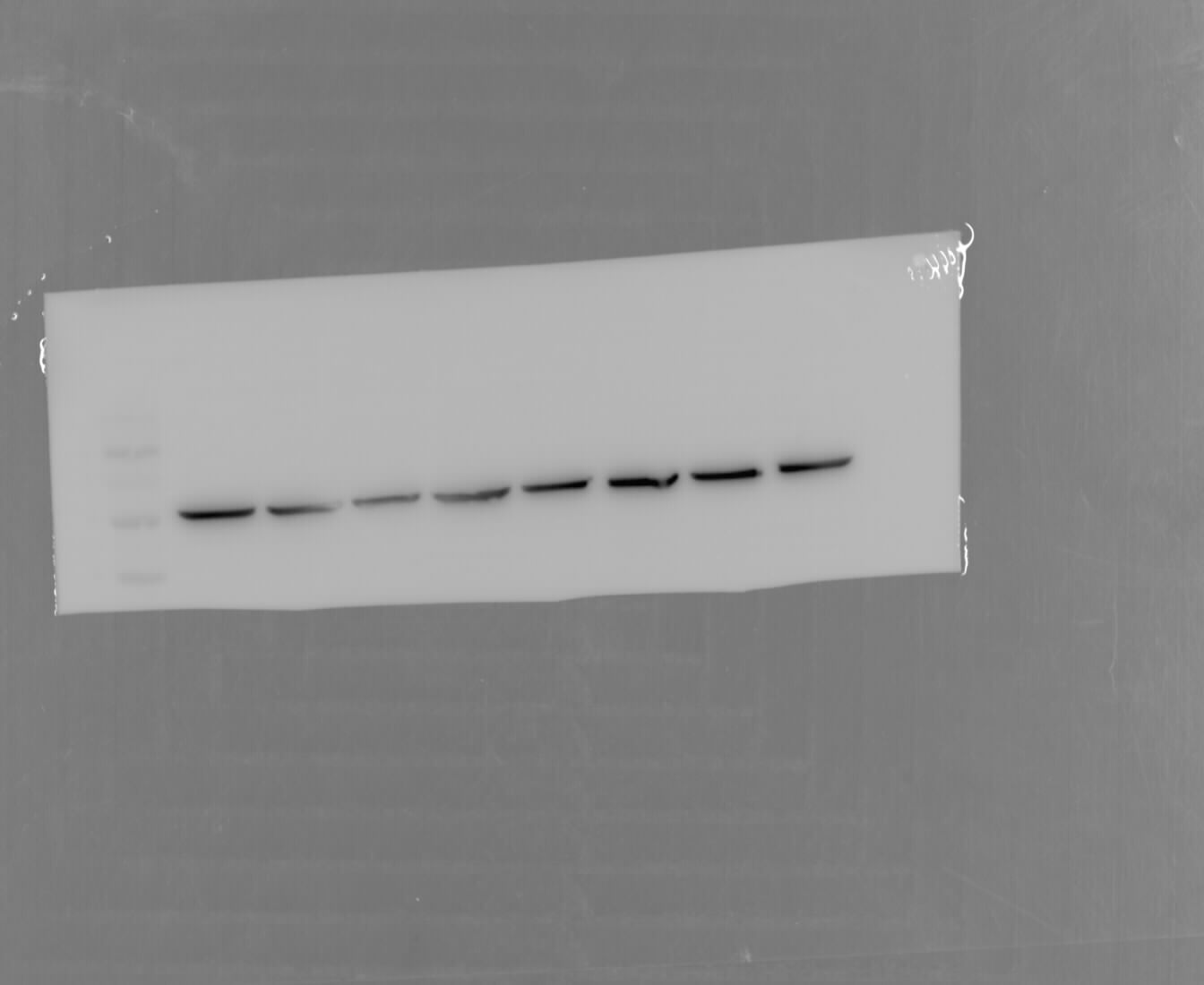

Supplement: Figure 1—source data 1. [file elife-70787-fig1-data1.zip › Figure 1/Figure 1E Tubulin.JPG]

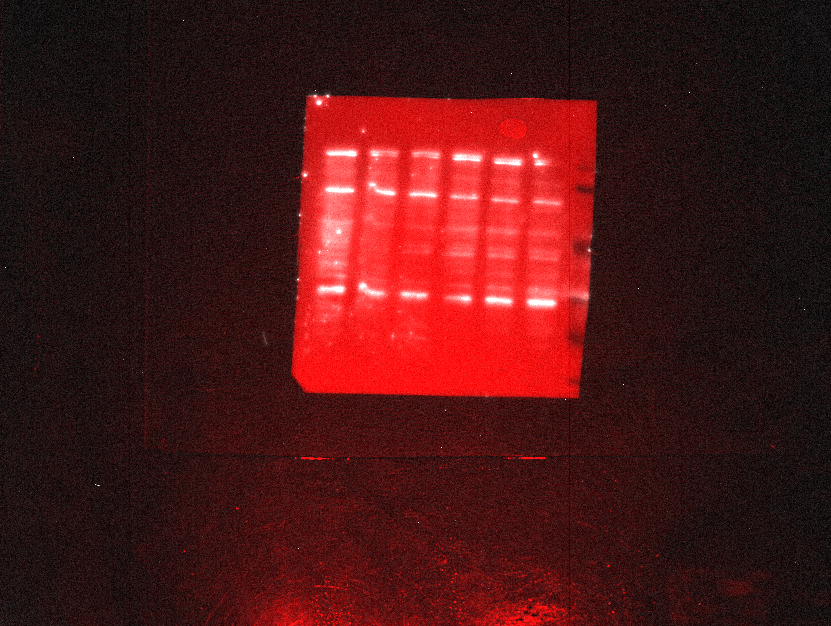

Supplement: Figure 1—source data 1. [file elife-70787-fig1-data1.zip › Figure 1/Figure 1G Hst4.png]

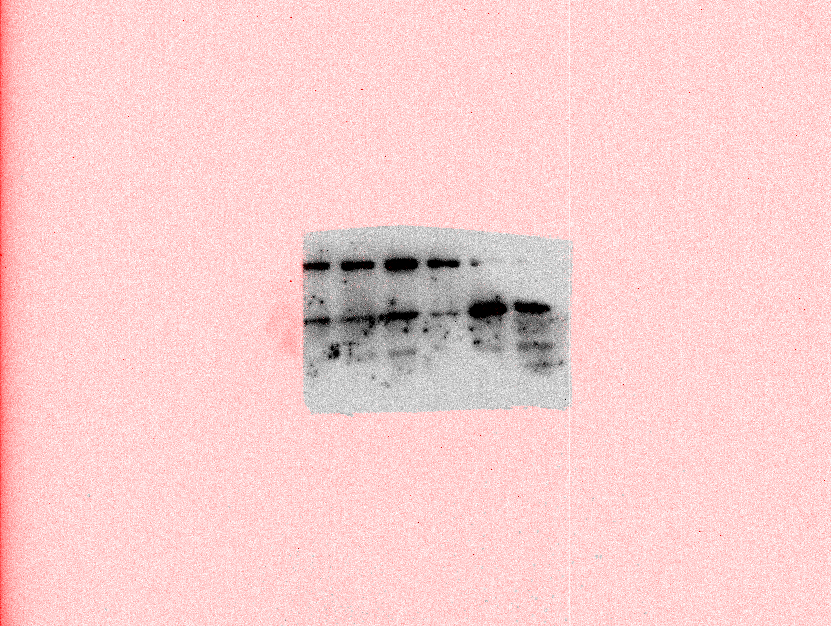

Supplement: Figure 2—source data 1. [file elife-70787-fig2-data1.zip › Figure 2/Figure 2B Hst4.png]

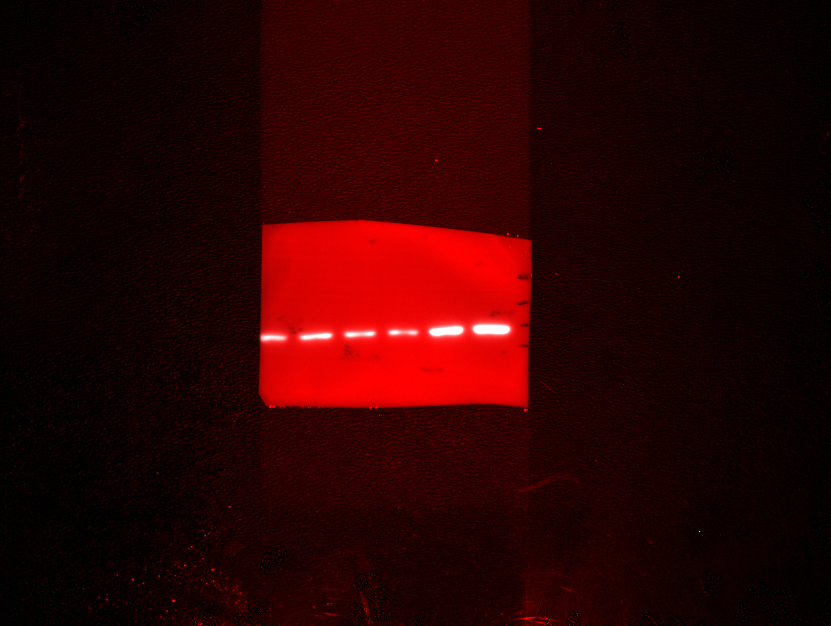

Supplement: Figure 2—source data 1. [file elife-70787-fig2-data1.zip › Figure 2/Figure 2B Tubulin.png]

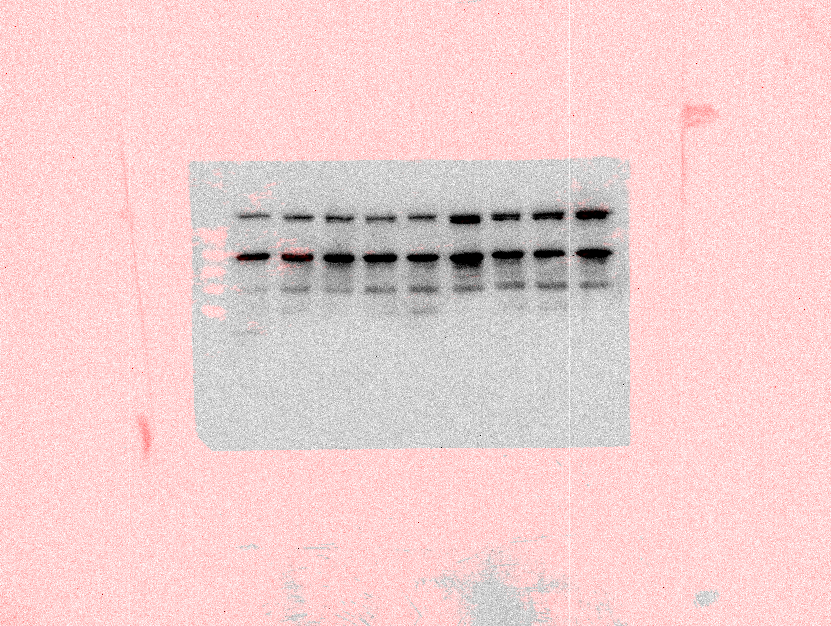

Supplement: Figure 2—source data 1. [file elife-70787-fig2-data1.zip › Figure 2/Figure 2C Hst4.png]

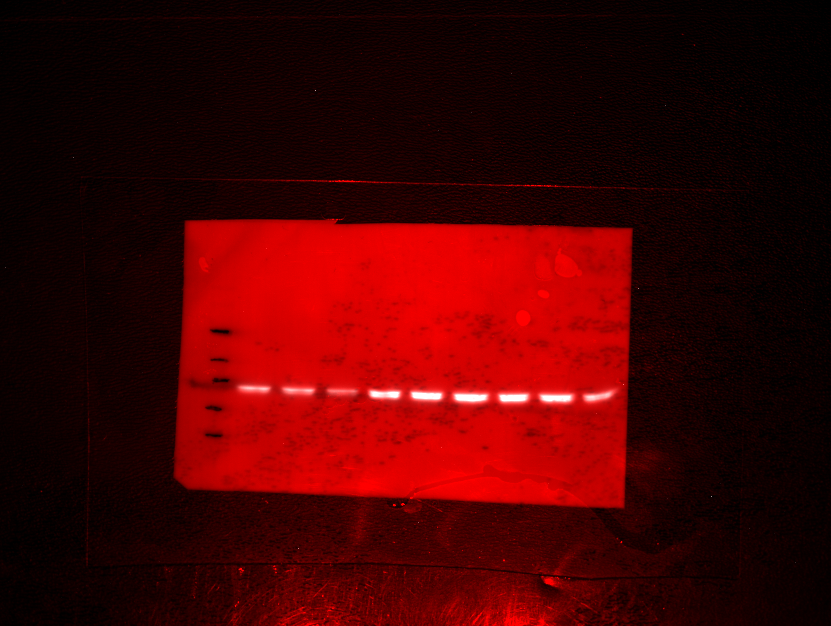

Supplement: Figure 2—source data 1. [file elife-70787-fig2-data1.zip › Figure 2/Figure 2C Tubulin.png]

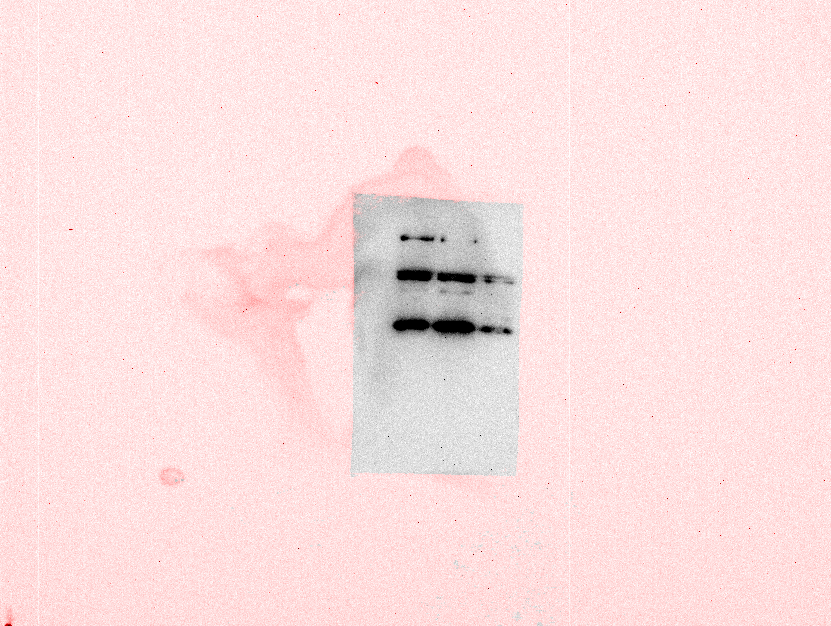

Supplement: Figure 2—source data 1. [file elife-70787-fig2-data1.zip › Figure 2/Figure 2D Hst4 input.png]

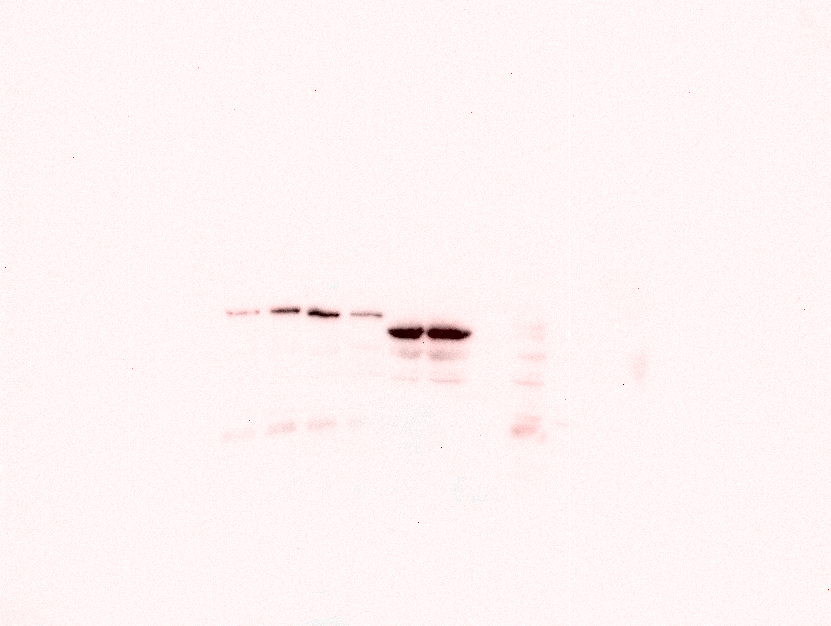

Supplement: Figure 2—source data 1. [file elife-70787-fig2-data1.zip › Figure 2/Figure 2E GFP-Hst4.png]

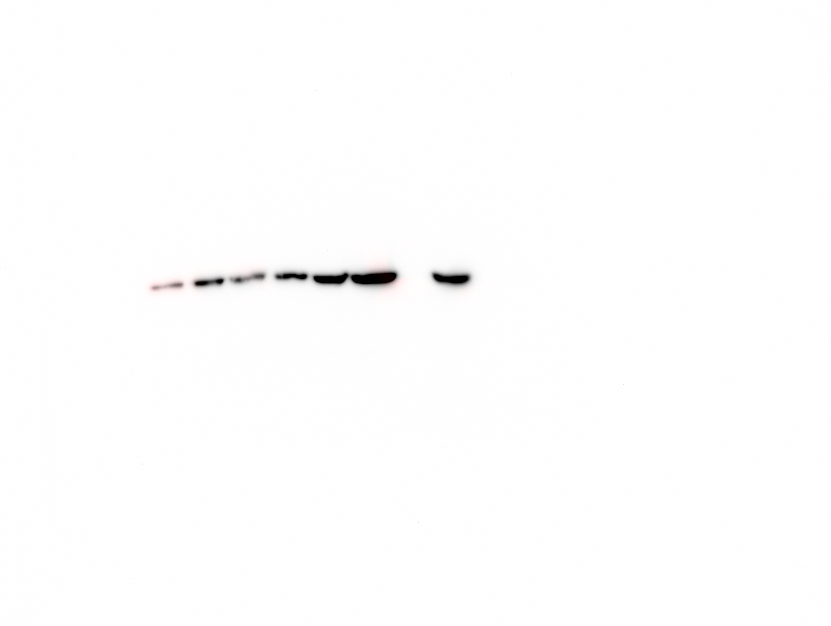

Supplement: Figure 2—source data 1. [file elife-70787-fig2-data1.zip › Figure 2/Figure 2E Tubulin.png]

Figure 2-figure supplement 1-source data 1

Figure 2-figure supplement 1D

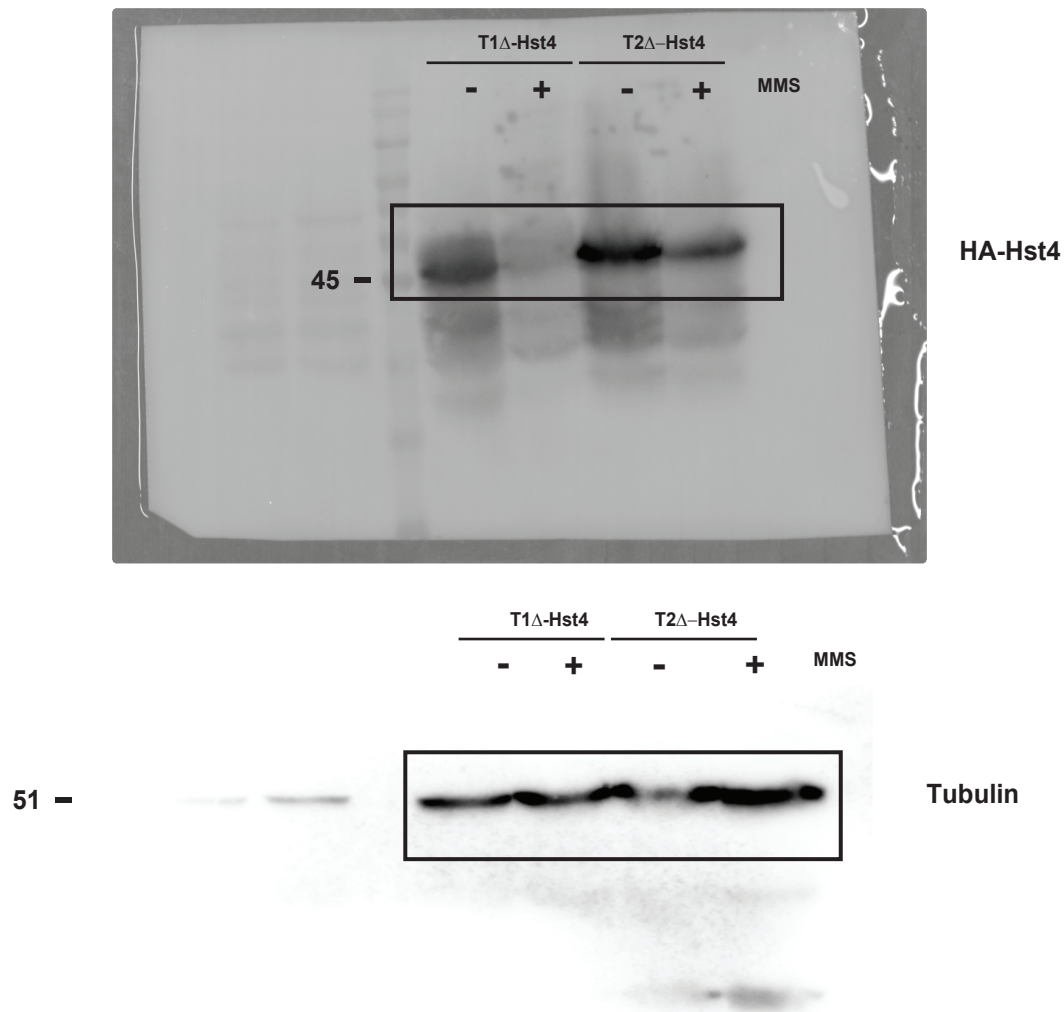

Supplement: Figure 2—figure supplement 1—source data 1. [file elife-70787-fig2-figsupp1-data1.pdf]

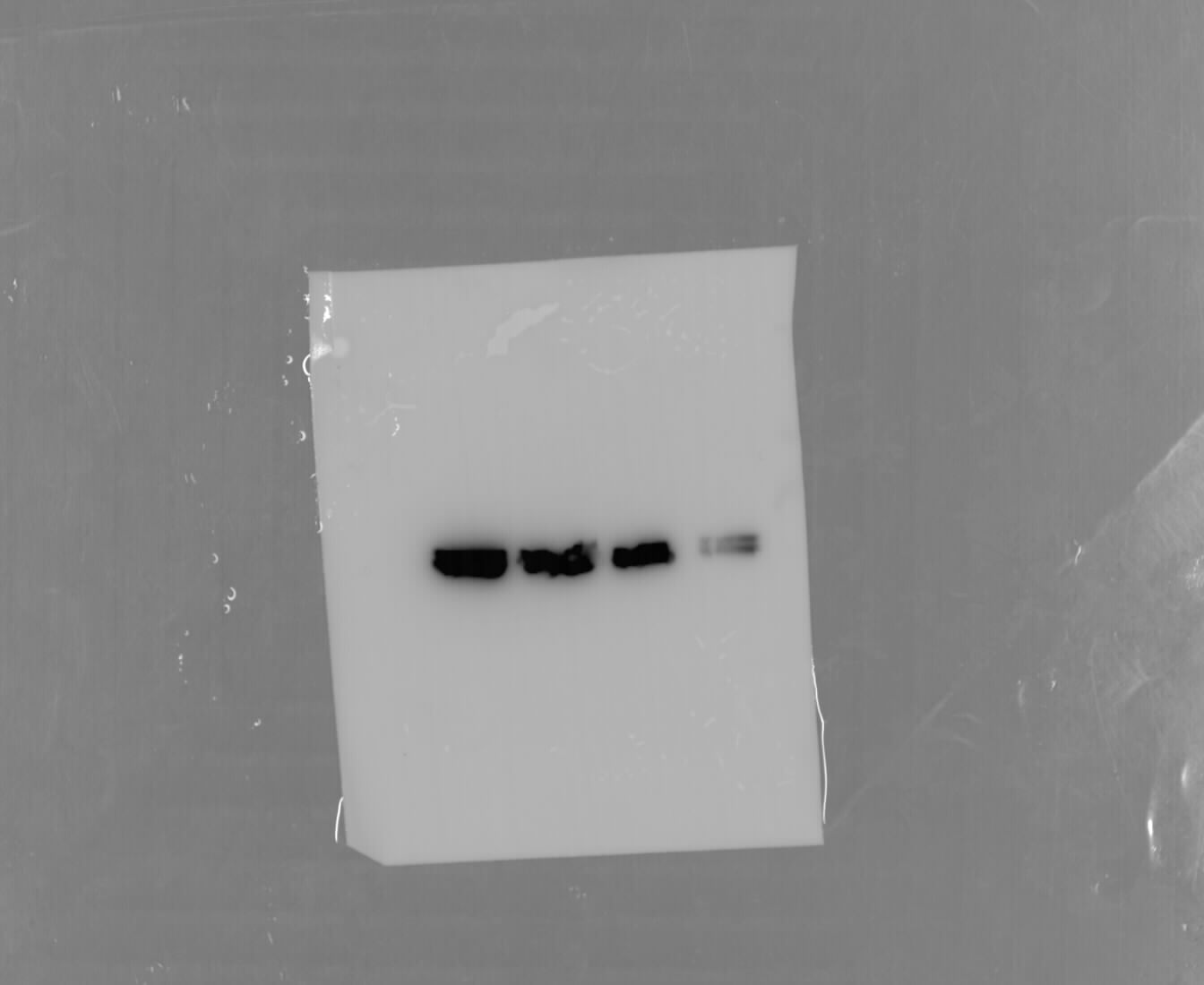

Supplement: Figure 3—source data 1. [file elife-70787-fig3-data1.zip › Figure 3A-G/Figure 3C Hst4 input.JPG]

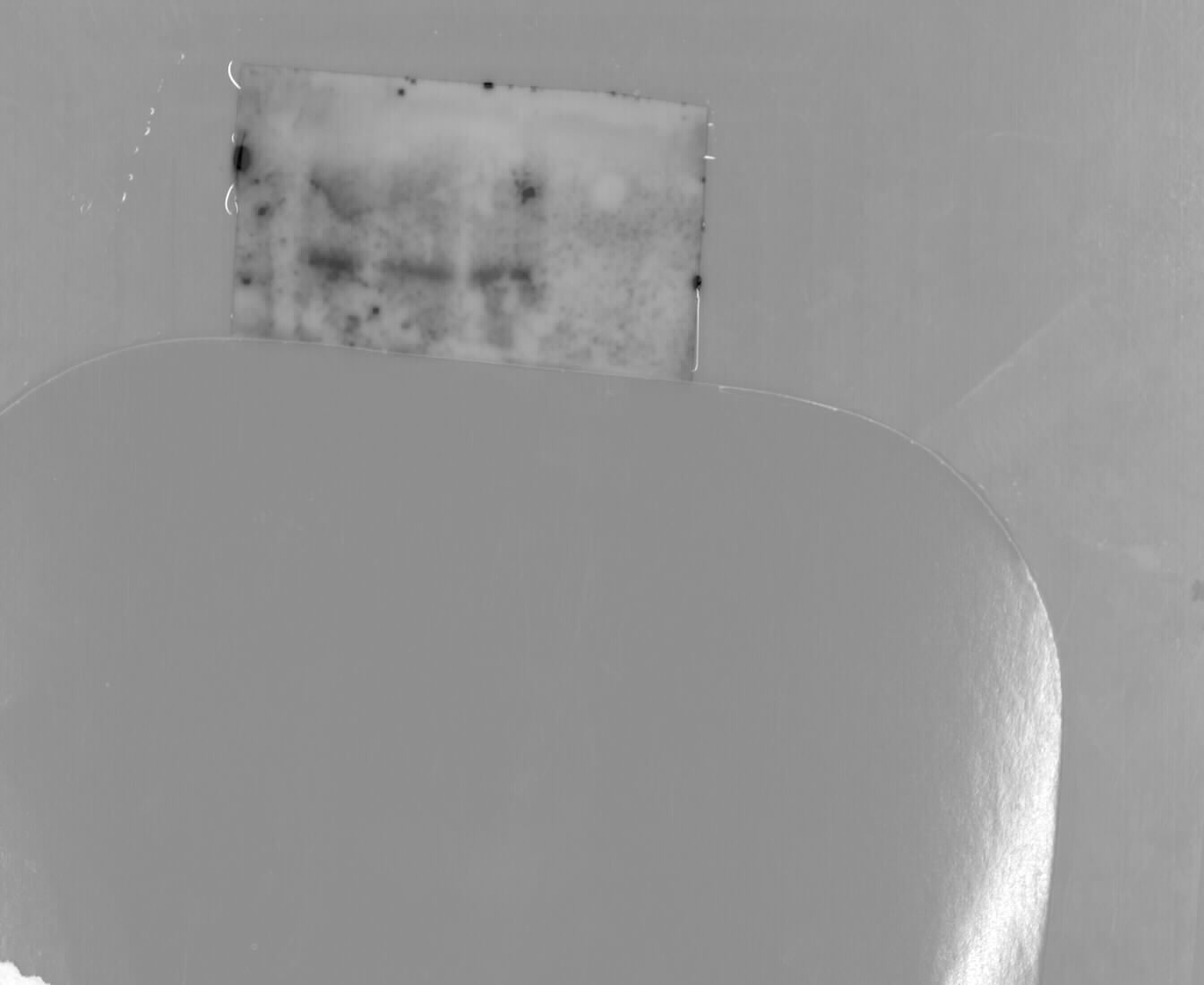

Supplement: Figure 3—source data 1. [file elife-70787-fig3-data1.zip › Figure 3A-G/Figure 3C Hst4 IP.JPG]

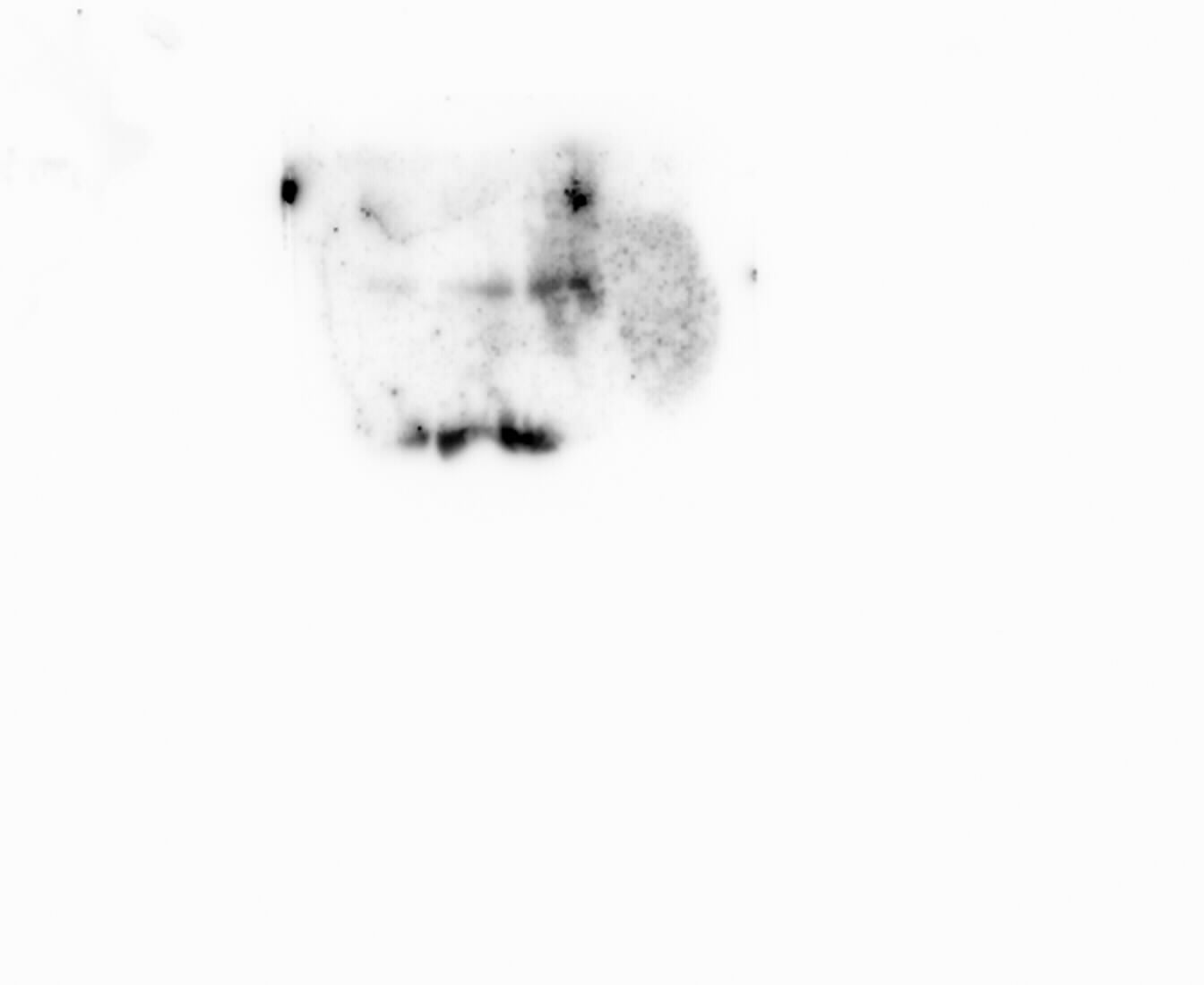

Supplement: Figure 3—source data 1. [file elife-70787-fig3-data1.zip › Figure 3A-G/Figure 3C p-serine.JPG]

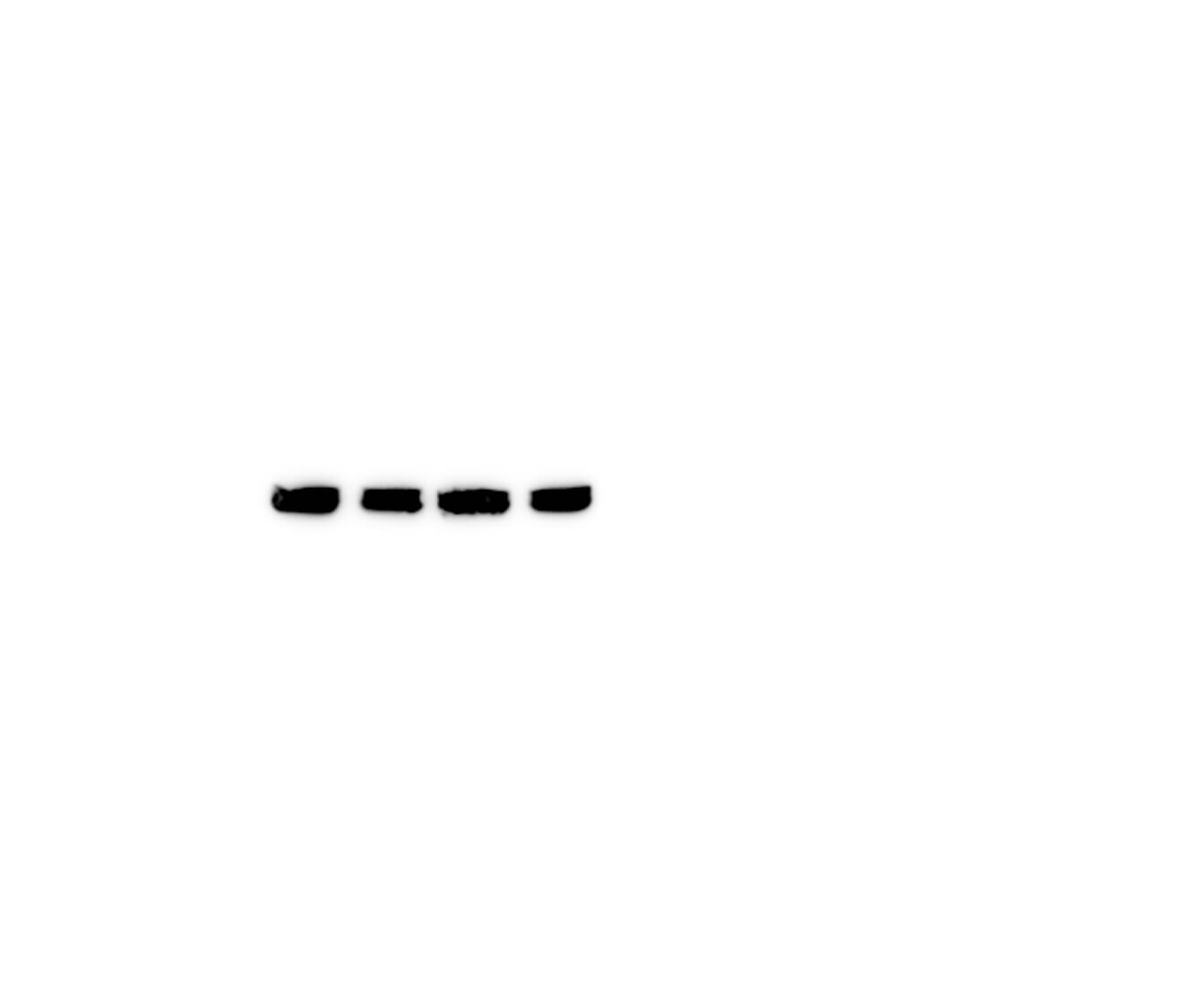

Supplement: Figure 3—source data 1. [file elife-70787-fig3-data1.zip › Figure 3A-G/Figure 3C Tubulin.JPG]

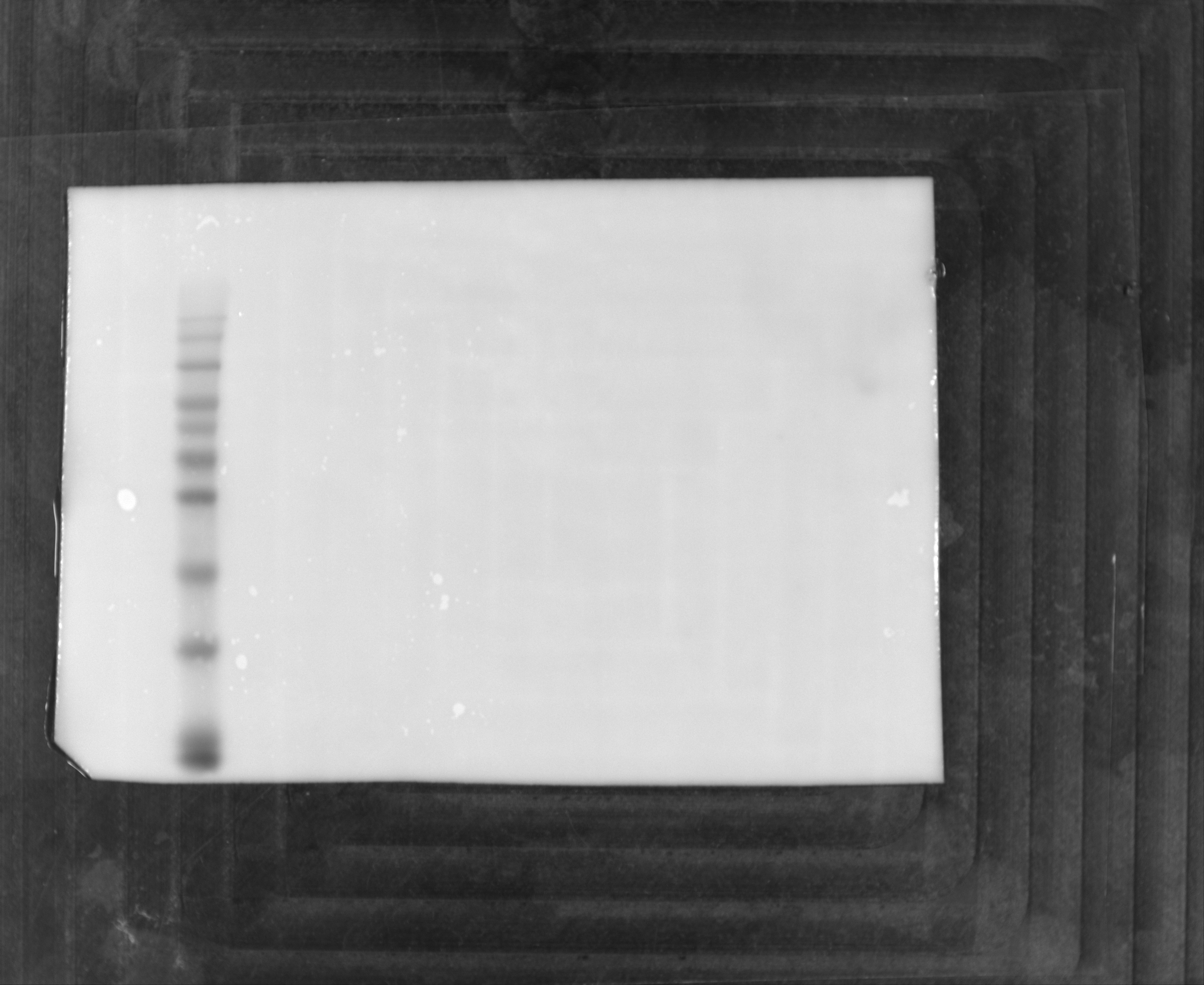

Supplement: Figure 3—source data 1. [file elife-70787-fig3-data1.zip › Figure 3A-G/Figure 3D Hst4 marker image.Tif]

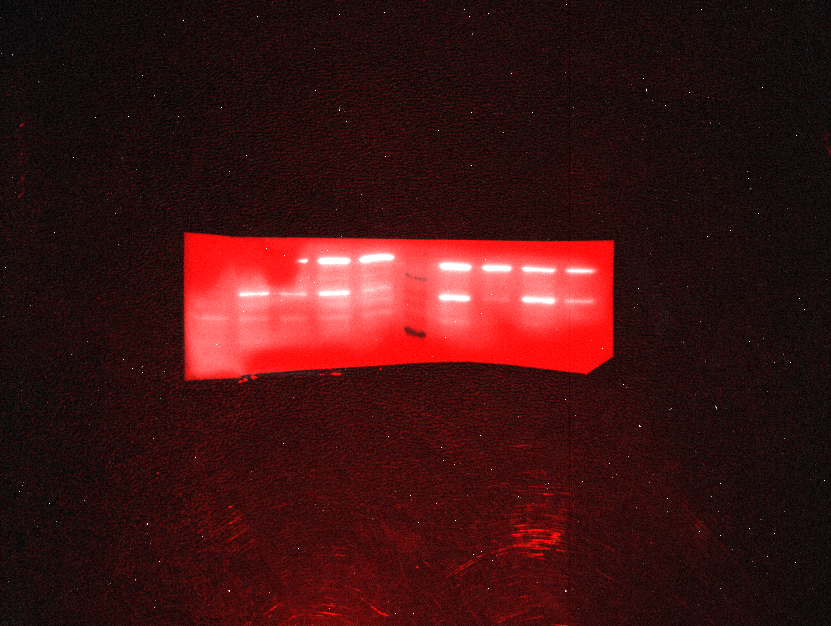

Supplement: Figure 3—source data 2. [file elife-70787-fig3-data2.zip › Figure 3_2/Figure 3-Supplemental 1B Hst4.png]

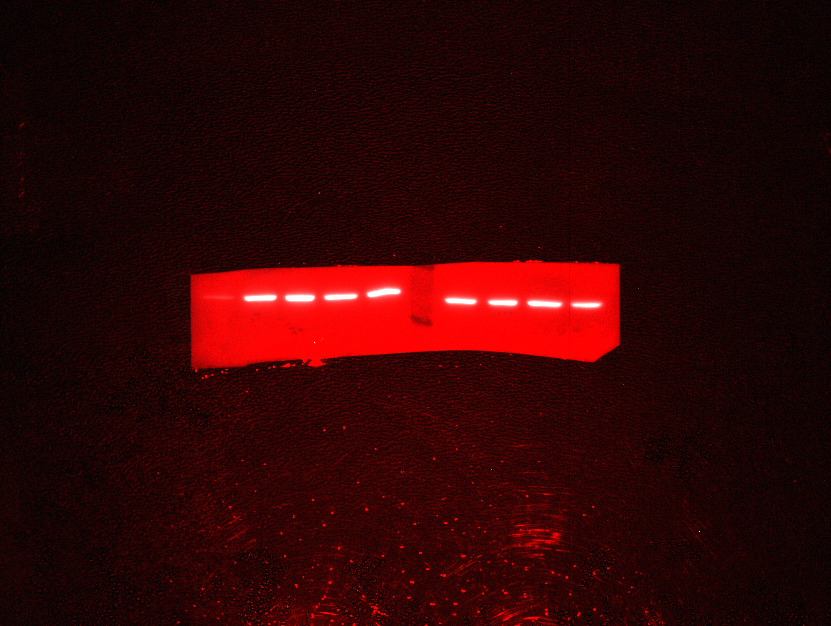

Supplement: Figure 3—source data 2. [file elife-70787-fig3-data2.zip › Figure 3_2/Figure 3-Supplemental 1B Tubulin.png]

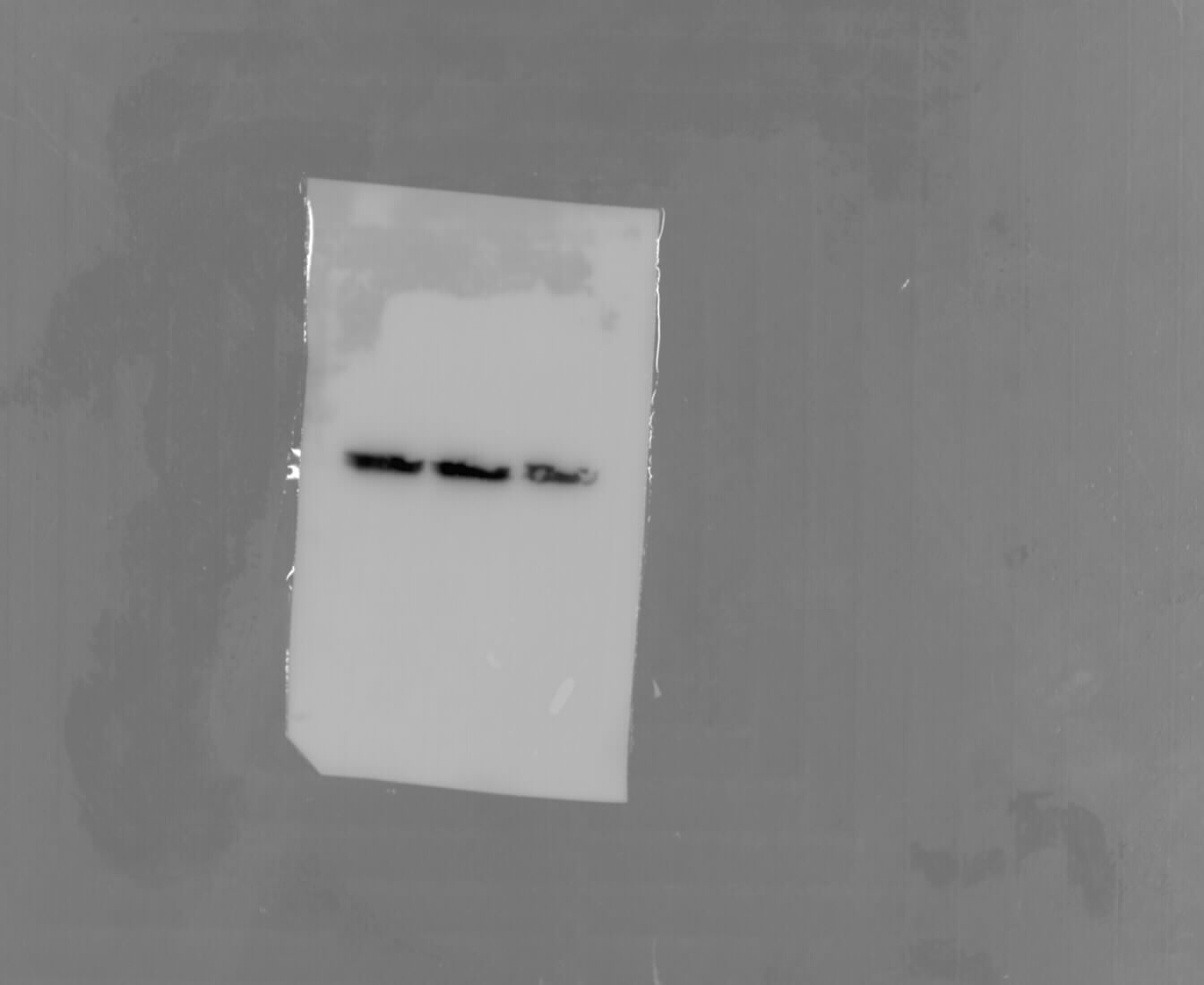

Supplement: Figure 3—source data 2. [file elife-70787-fig3-data2.zip › Figure 3_2/Figure 3H Hst4 input.JPG]

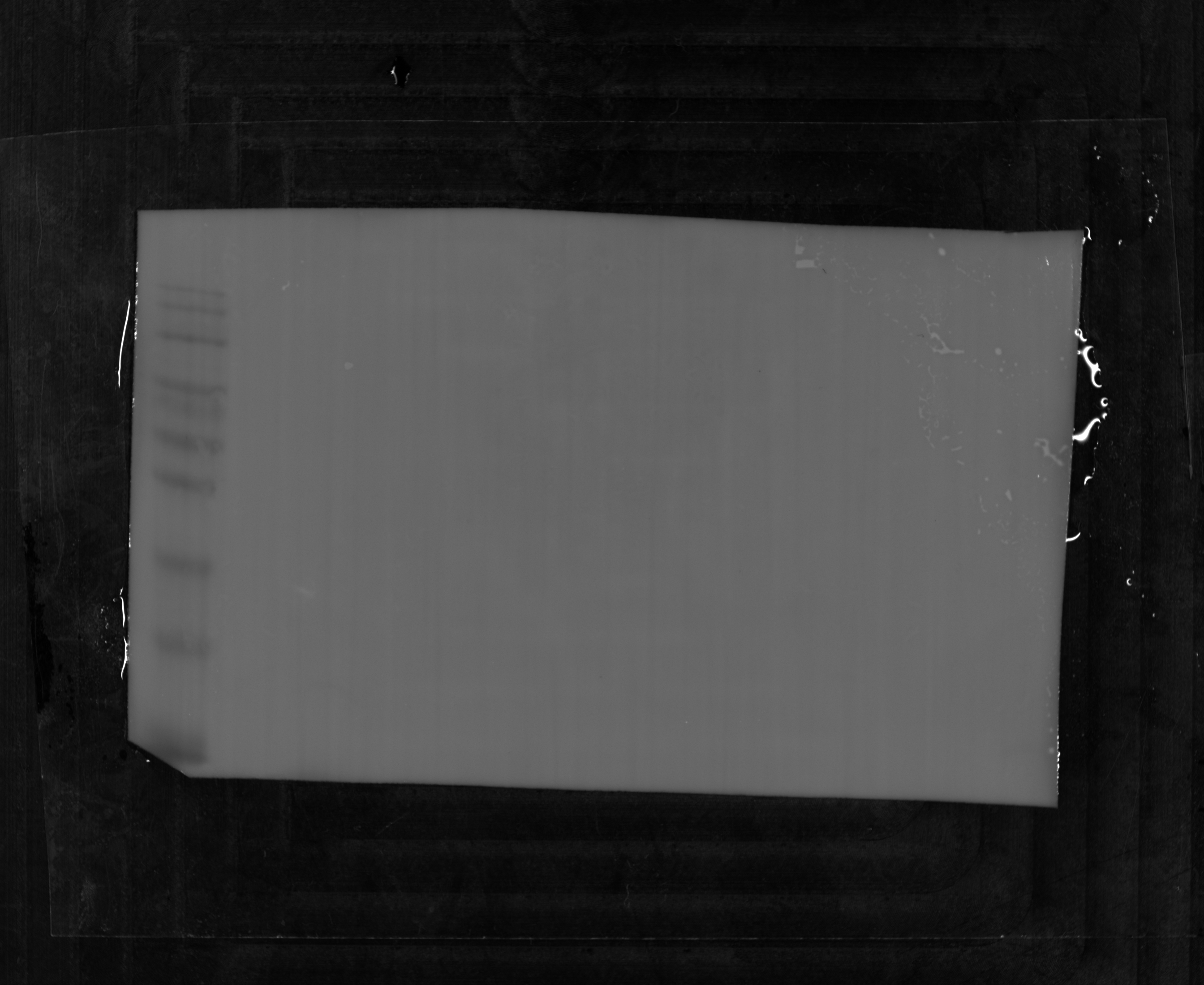

Supplement: Figure 4—source data 1. [file elife-70787-fig4-data1.zip › Figure 4/Figure 4C Marker.Tif]

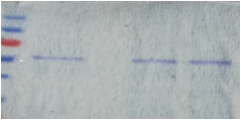

Supplement: Figure 4—source data 1. [file elife-70787-fig4-data1.zip › Figure 4/Figure 4E Purified Hst4 FL 4SA.tif]

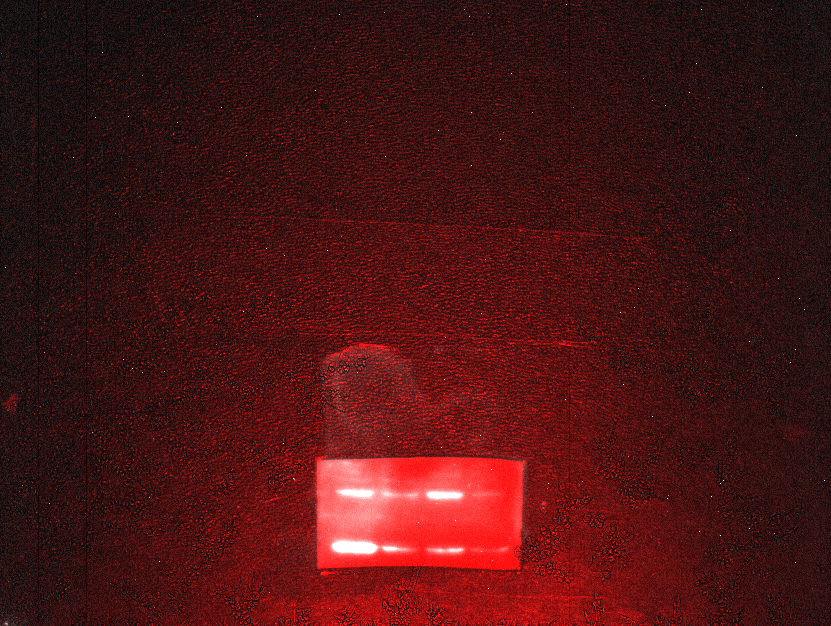

Supplement: Figure 5—source data 1. [file elife-70787-fig5-data1.zip › Figure 5/Figure 5-Supplemental 1C Hst4.png]

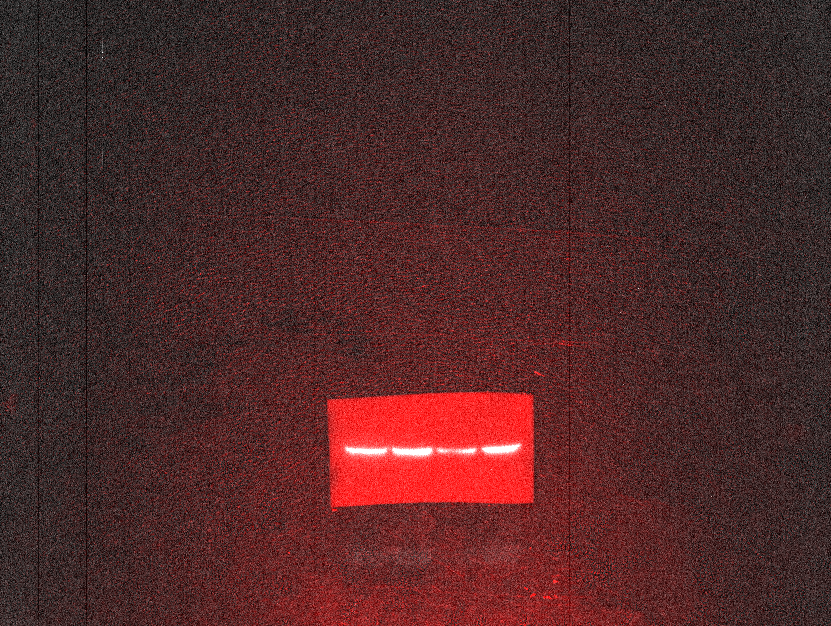

Supplement: Figure 5—source data 1. [file elife-70787-fig5-data1.zip › Figure 5/Figure 5-Supplemental 1C Tubulin.png]

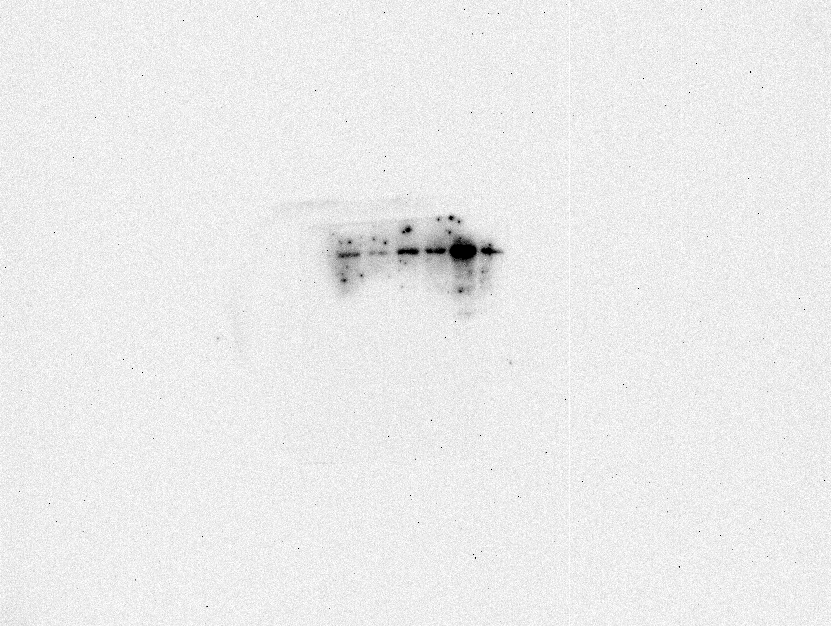

Supplement: Figure 5—source data 1. [file elife-70787-fig5-data1.zip › Figure 5/Figure 5A Hst4.png]

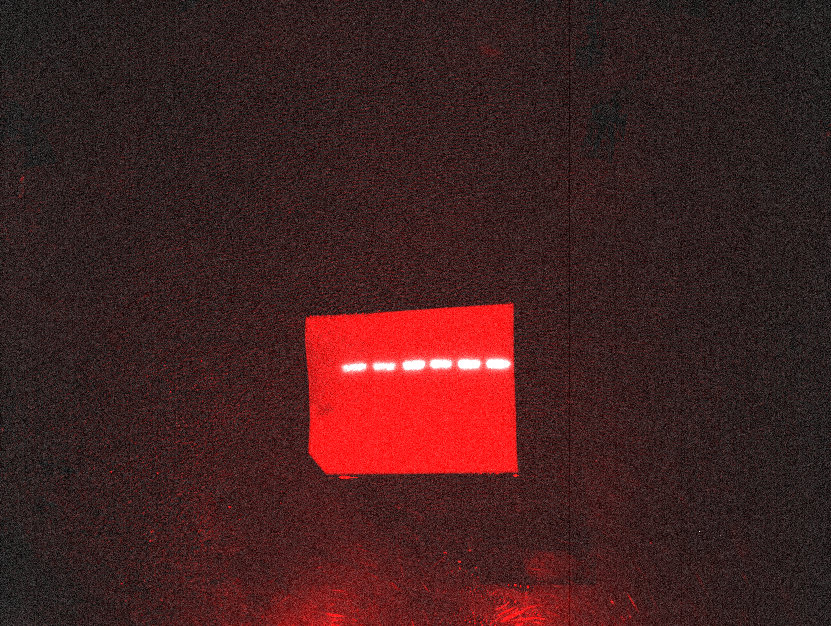

Supplement: Figure 5—source data 1. [file elife-70787-fig5-data1.zip › Figure 5/Figure 5A Tubulin.png]

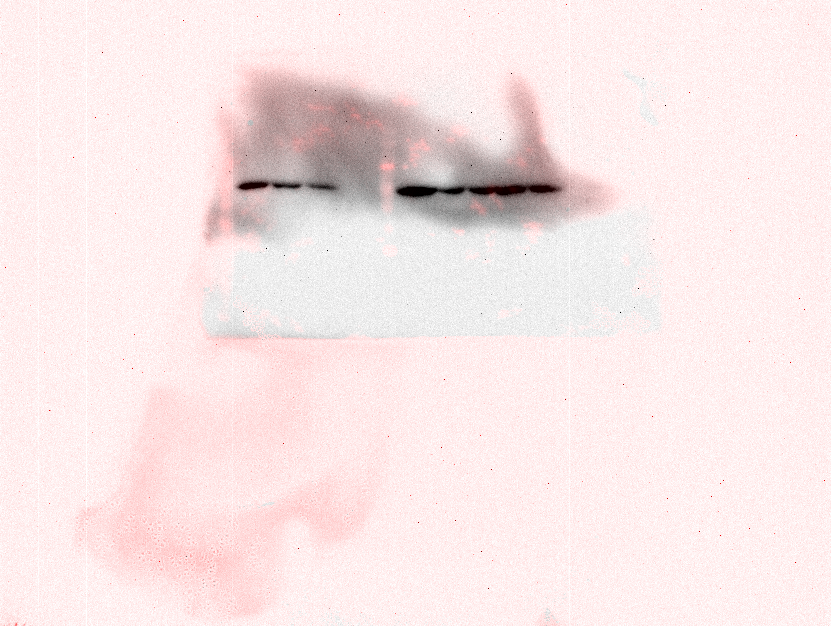

Supplement: Figure 5—source data 1. [file elife-70787-fig5-data1.zip › Figure 5/Figure 5B Hst4.png]

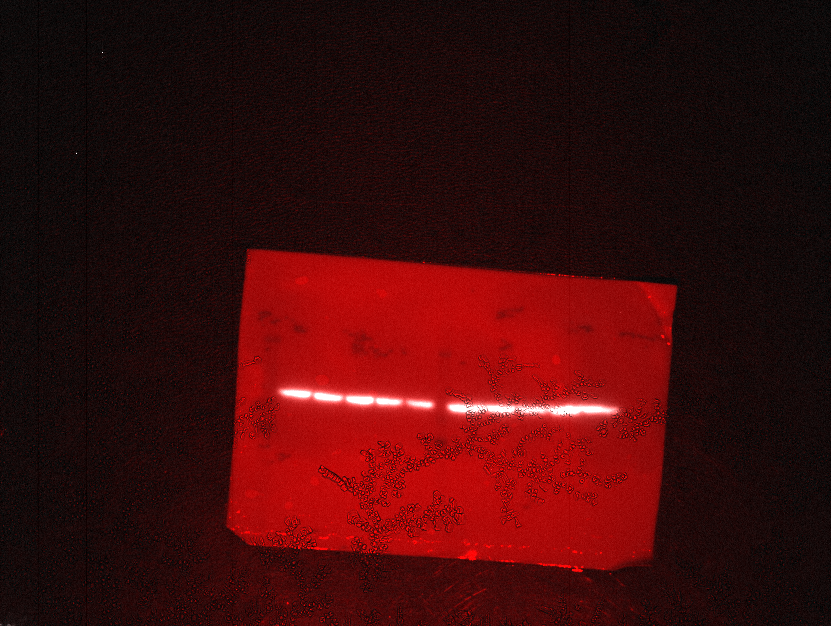

Supplement: Figure 5—source data 1. [file elife-70787-fig5-data1.zip › Figure 5/Figure 5B Tubulin.png]

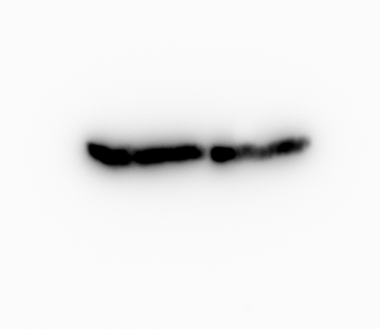

Supplement: Figure 5—source data 1. [file elife-70787-fig5-data1.zip › Figure 5/Figure 5E TAP HST4 INPUT.Tif]

Figure 5-Figure Supplement 1C

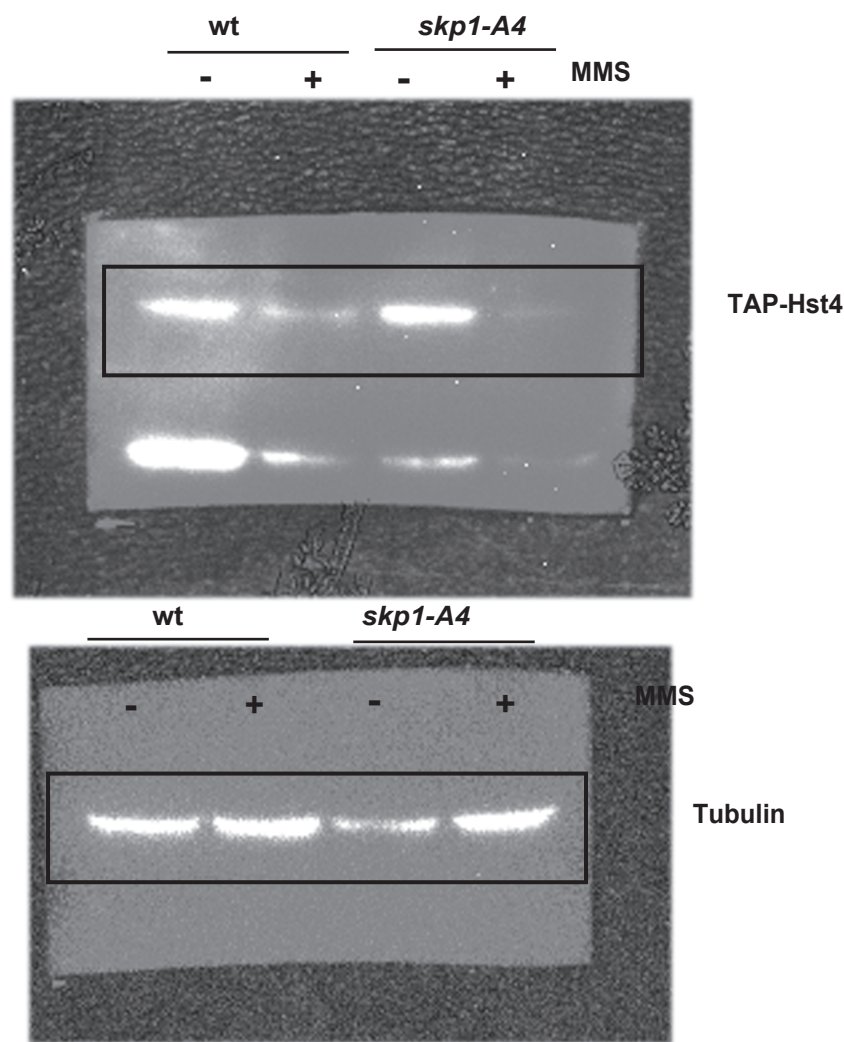

Supplement: Figure 5—figure supplement 1—source data 1. [file elife-70787-fig5-figsupp1-data1.pdf]

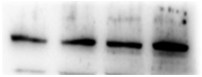

Supplement: Figure 7—source data 1. [file elife-70787-fig7-data1.zip › Figure 7/Figure 7C Mcl1.tif]

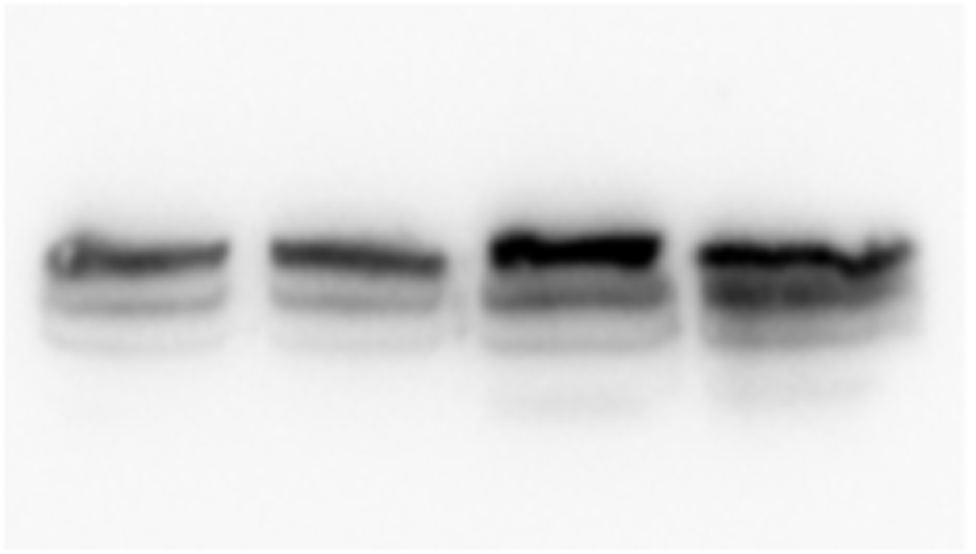

Supplement: Figure 7—source data 1. [file elife-70787-fig7-data1.zip › Figure 7/Figure 7C Swi1-FLAG.tif]

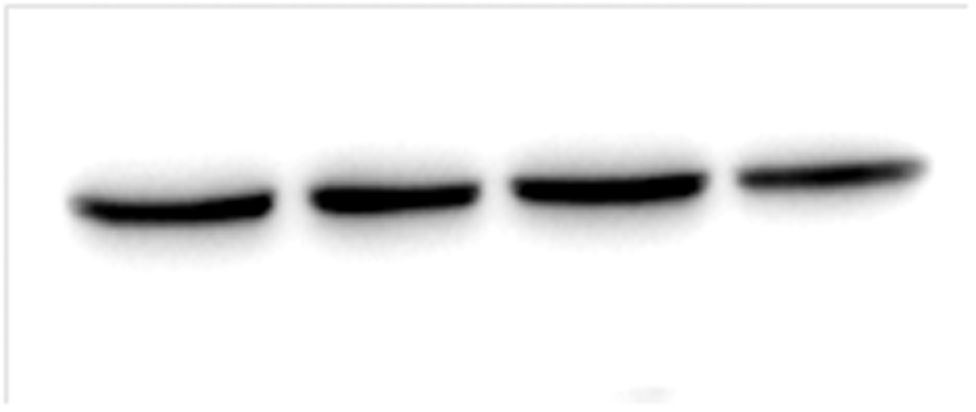

Supplement: Figure 7—source data 1. [file elife-70787-fig7-data1.zip › Figure 7/Figure 7C Tubulin.tif]

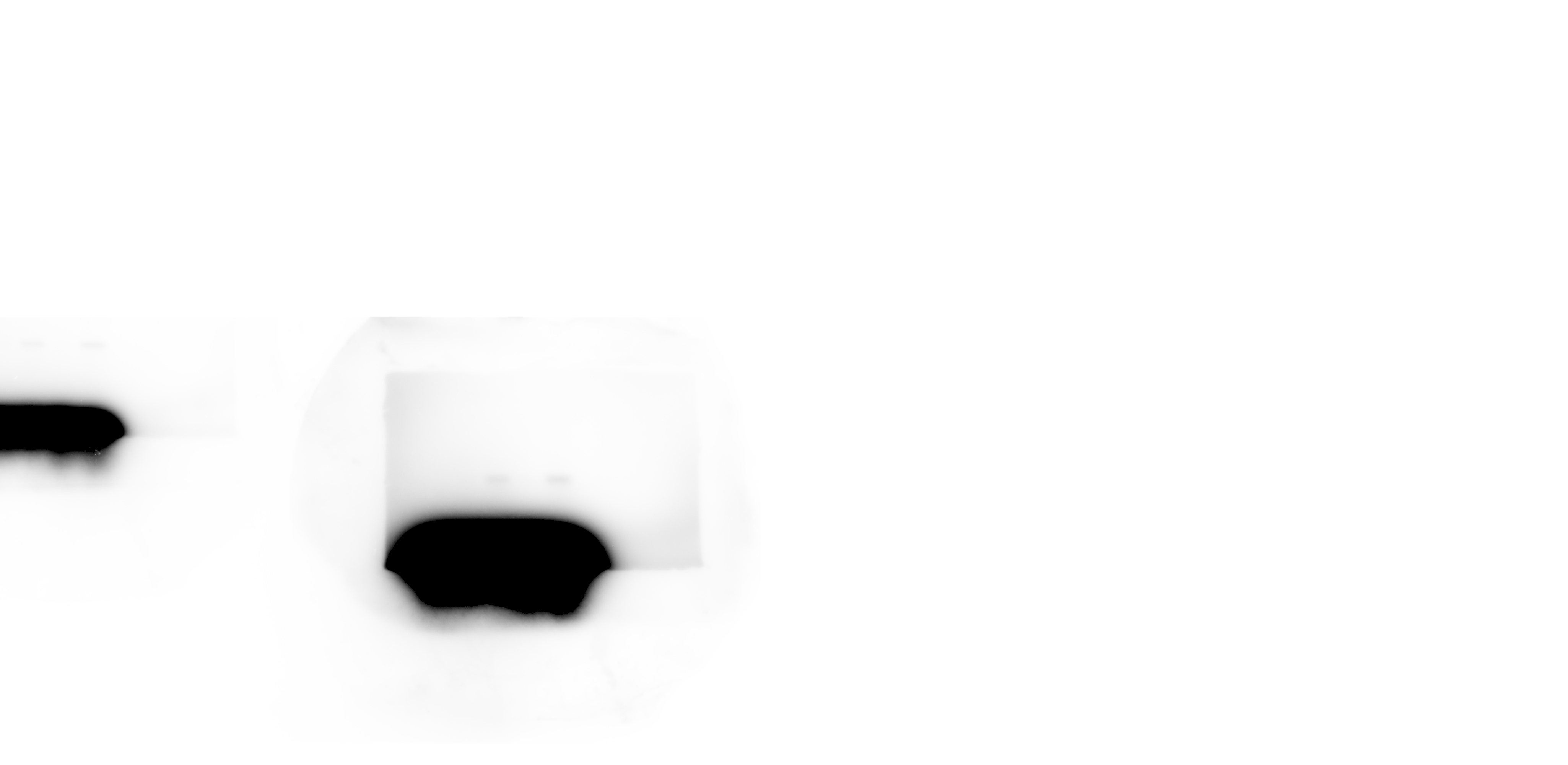

Supplement: Figure 7—source data 1. [file elife-70787-fig7-data1.zip › Figure 7/Figure 7F MBP-P.tif]

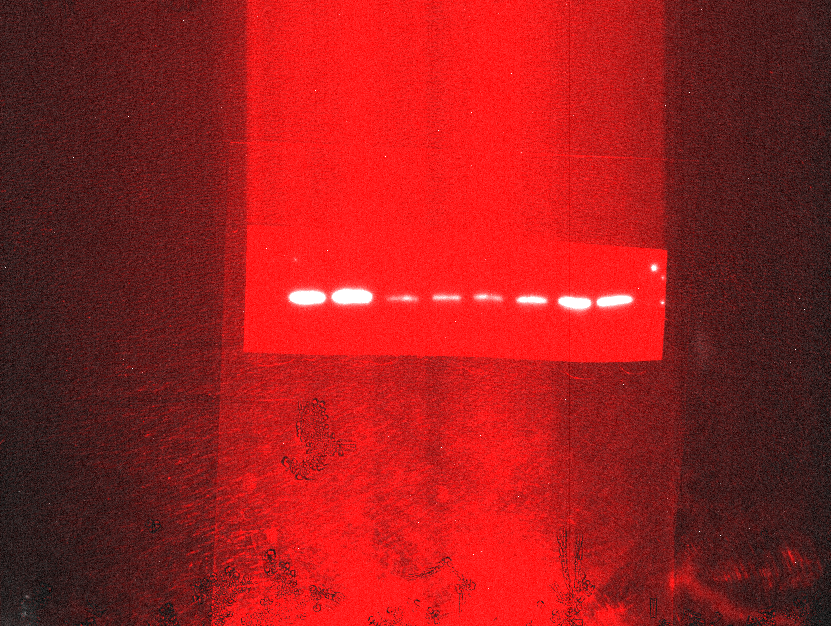

Supplement: Figure 8—source data 2. [file elife-70787-fig8-data2.zip › Figure 8_2/Figure 8 Supplement 1B H3K56ac.png]

Figure 8-figure supplement 1A

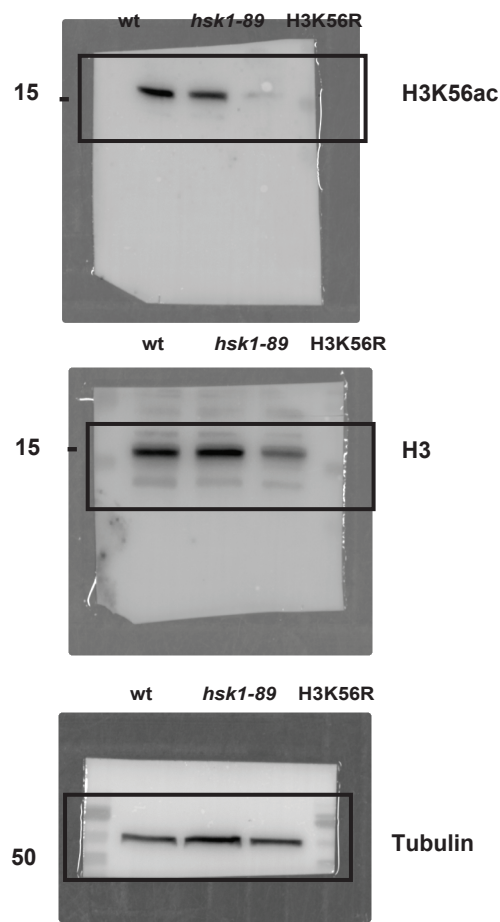

## Figure 8-figure supplement 1-source data 1

### Figure 8-figure supplement 1B

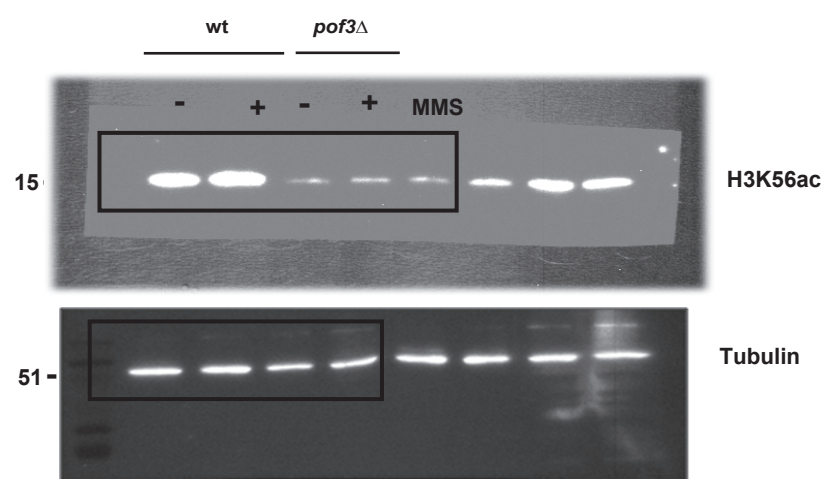

Supplement: Figure 8—figure supplement 1—source data 1. [file elife-70787-fig8-figsupp1-data1.pdf]
