# Supplementary material for: DDK/Hsk1 phosphorylates and targets fission yeast histone deacetylase Hst4 for degradation to stabilize stalled DNA replication forks
Source: eLife. 2021 Oct 5;10:e70787. doi: 10.7554/eLife.70787 (PMC8565929; doi:10.7554/eLife.70787)

Figure 4-figure supplement 1-source data 1

Figure 4-figure supplement 1A

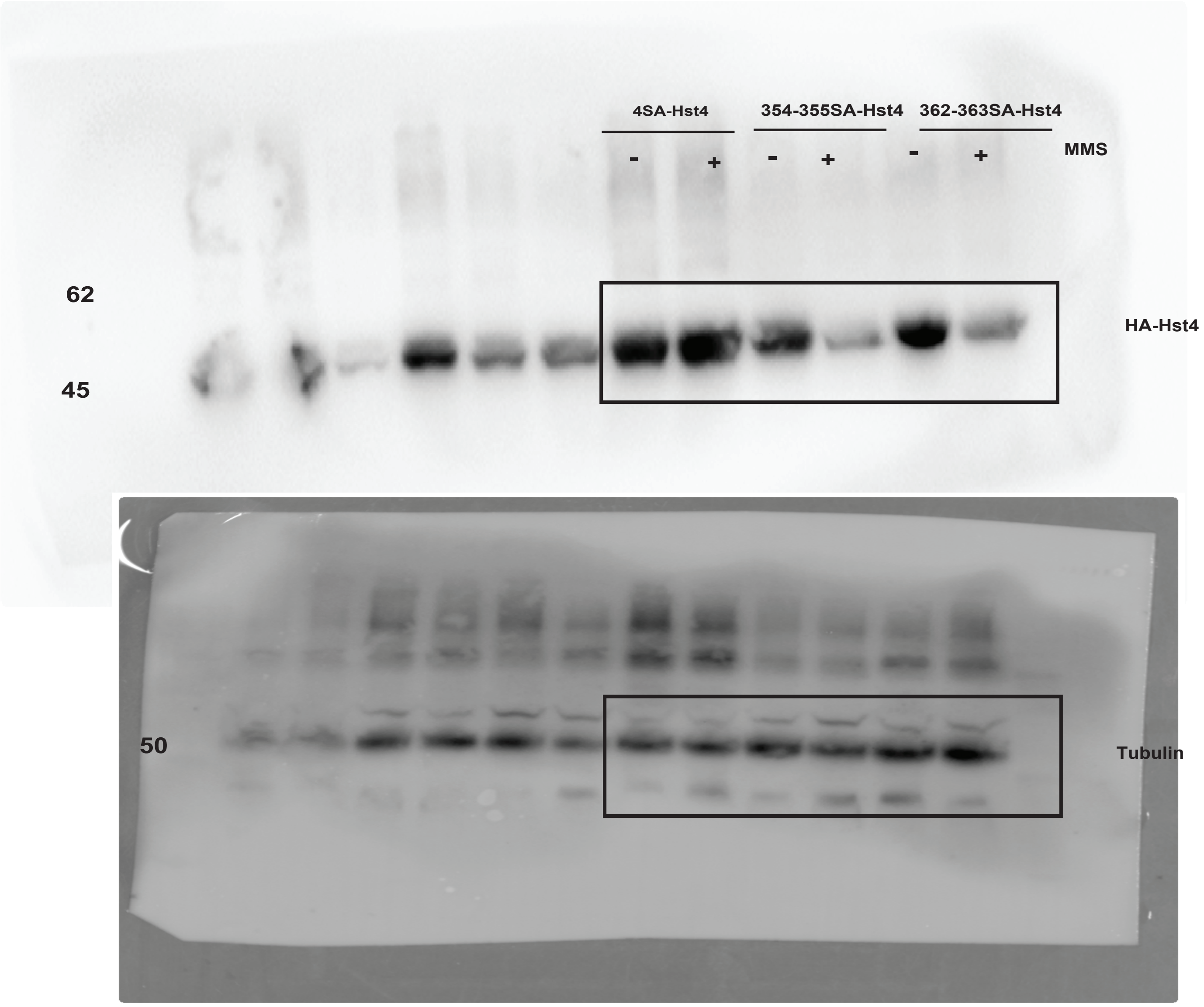

Figure 4-supplemental 1-source data 1

Figure 4-figure supplement 1B

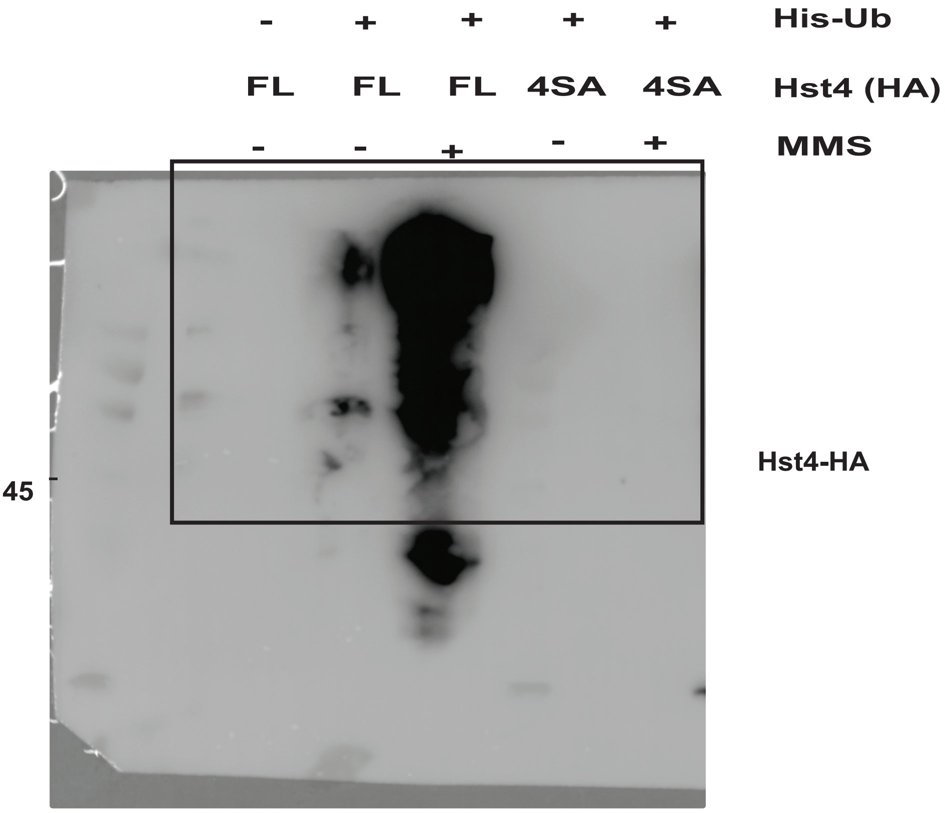

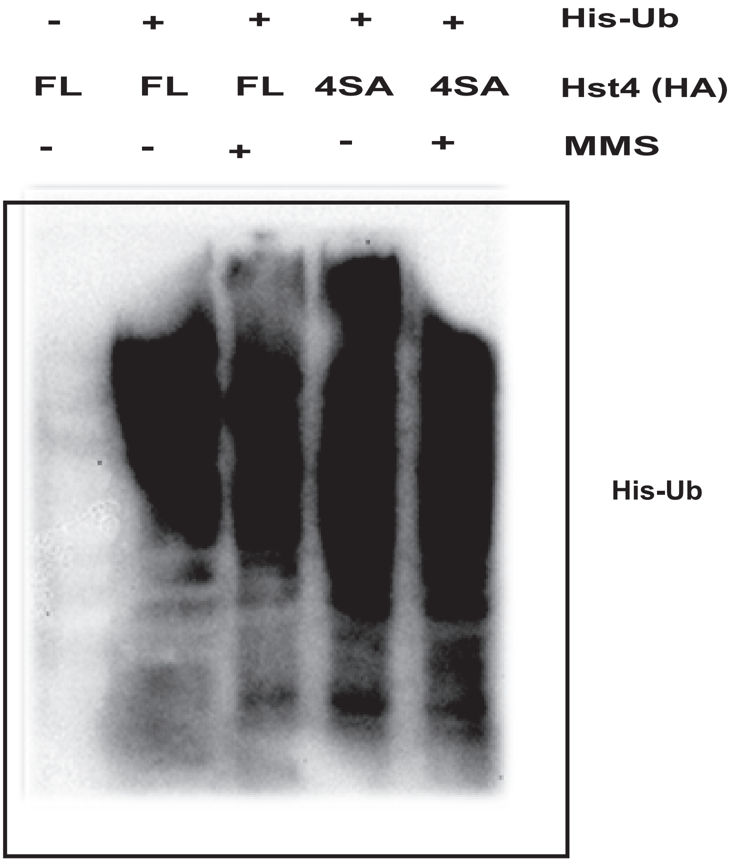

Supplement: Figure 4—figure supplement 1—source data 1. [file elife-70787-fig4-figsupp1-data1.pdf]
