# Supplementary material for: DDK/Hsk1 phosphorylates and targets fission yeast histone deacetylase Hst4 for degradation to stabilize stalled DNA replication forks
Source: eLife. 2021 Oct 5;10:e70787. doi: 10.7554/eLife.70787 (PMC8565929; doi:10.7554/eLife.70787)
Supplement: Source data 1. [file elife-70787-supp1.pdf]

Figure 1-source data 1

Figure 1A

MMS HU

\- + - +

62

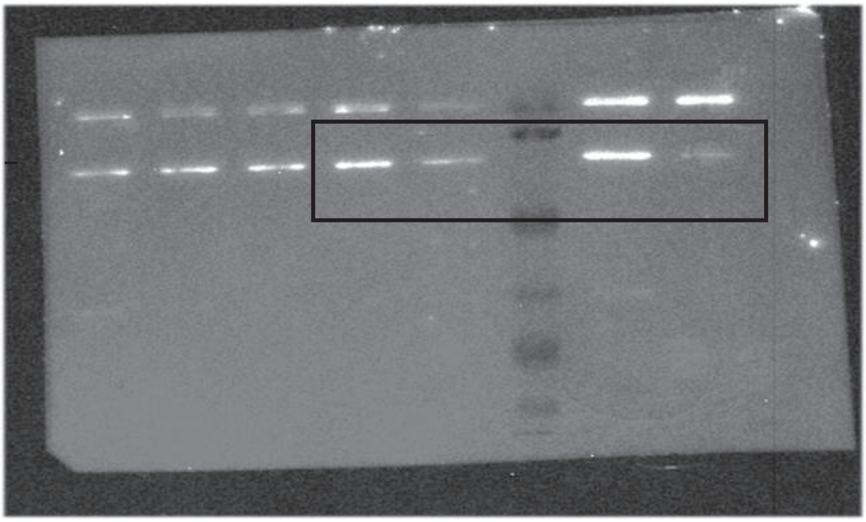

Hst4

50

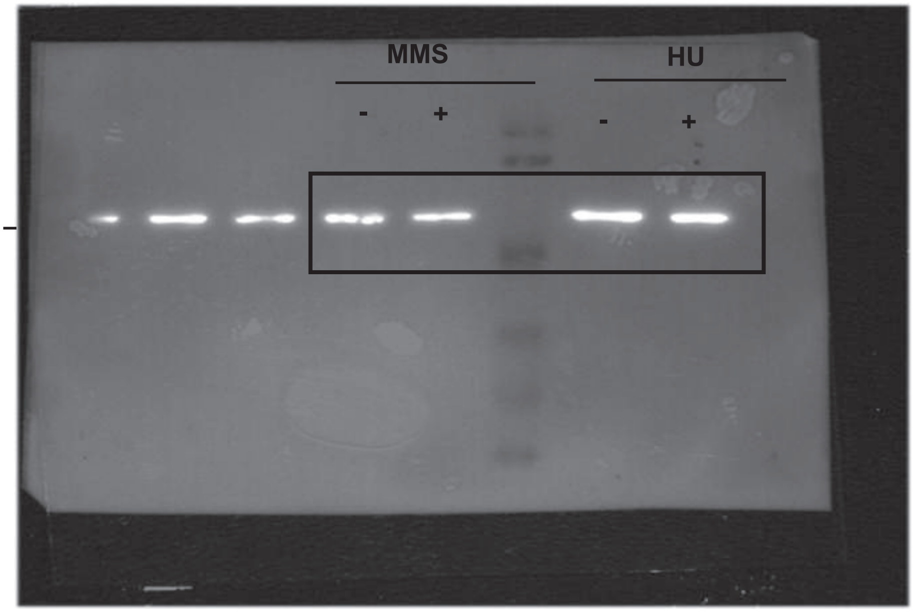

Tubulin

Figure 1-source data 1

Figure 1B

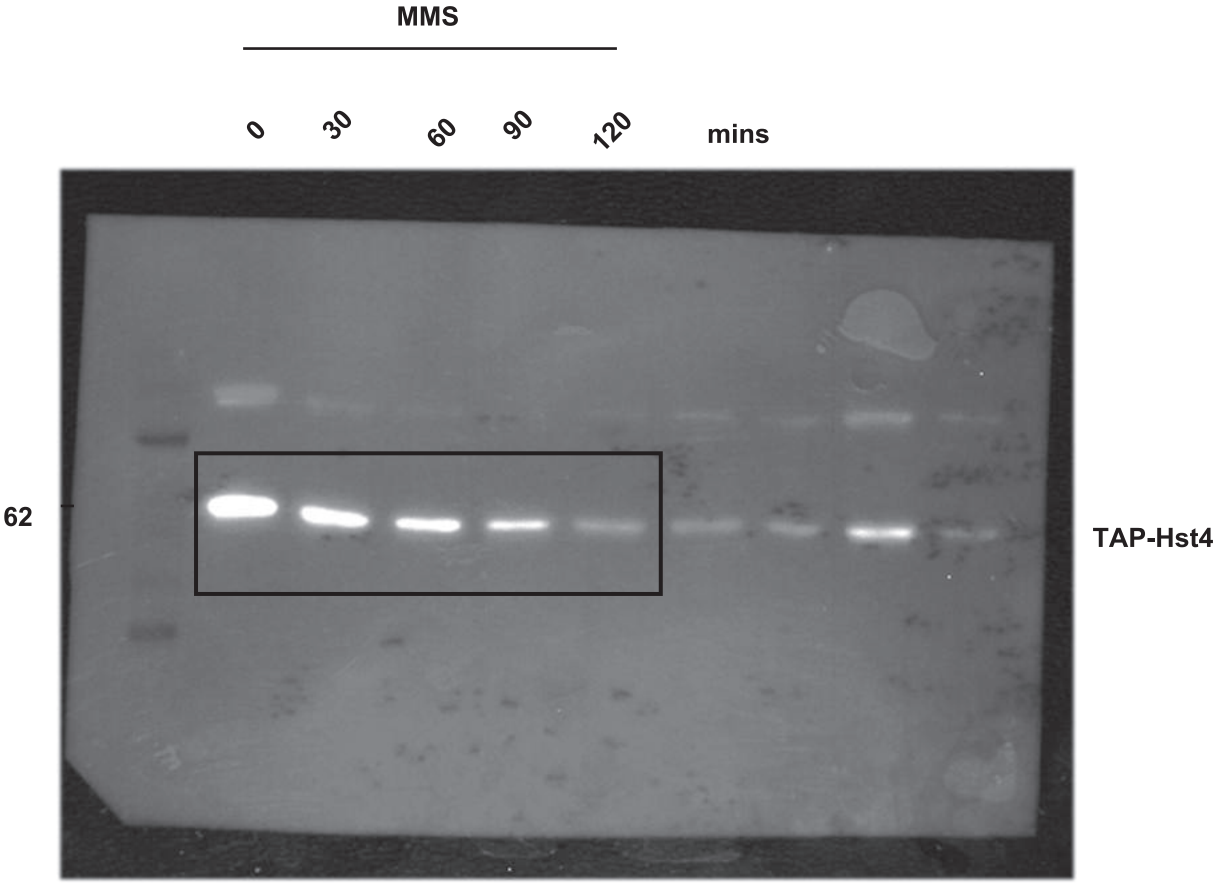

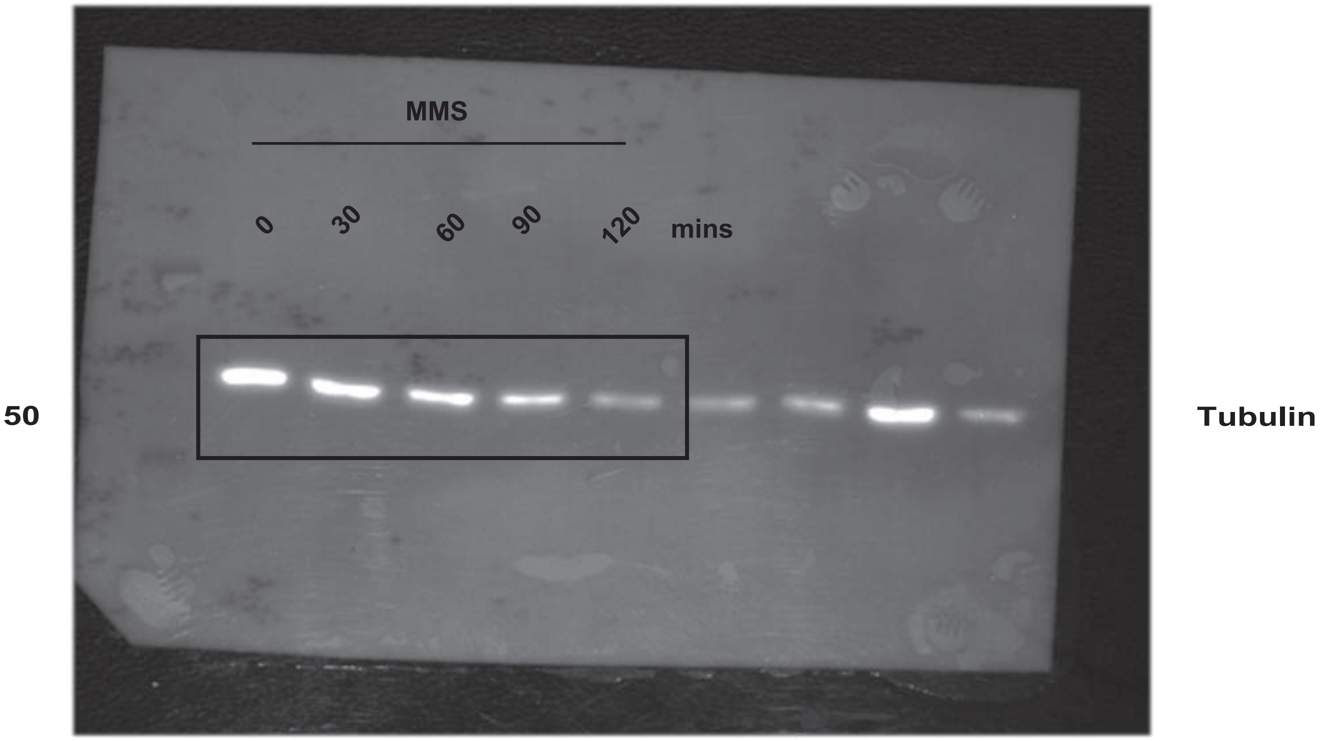

Figure 1-source data 1

Figure 1C

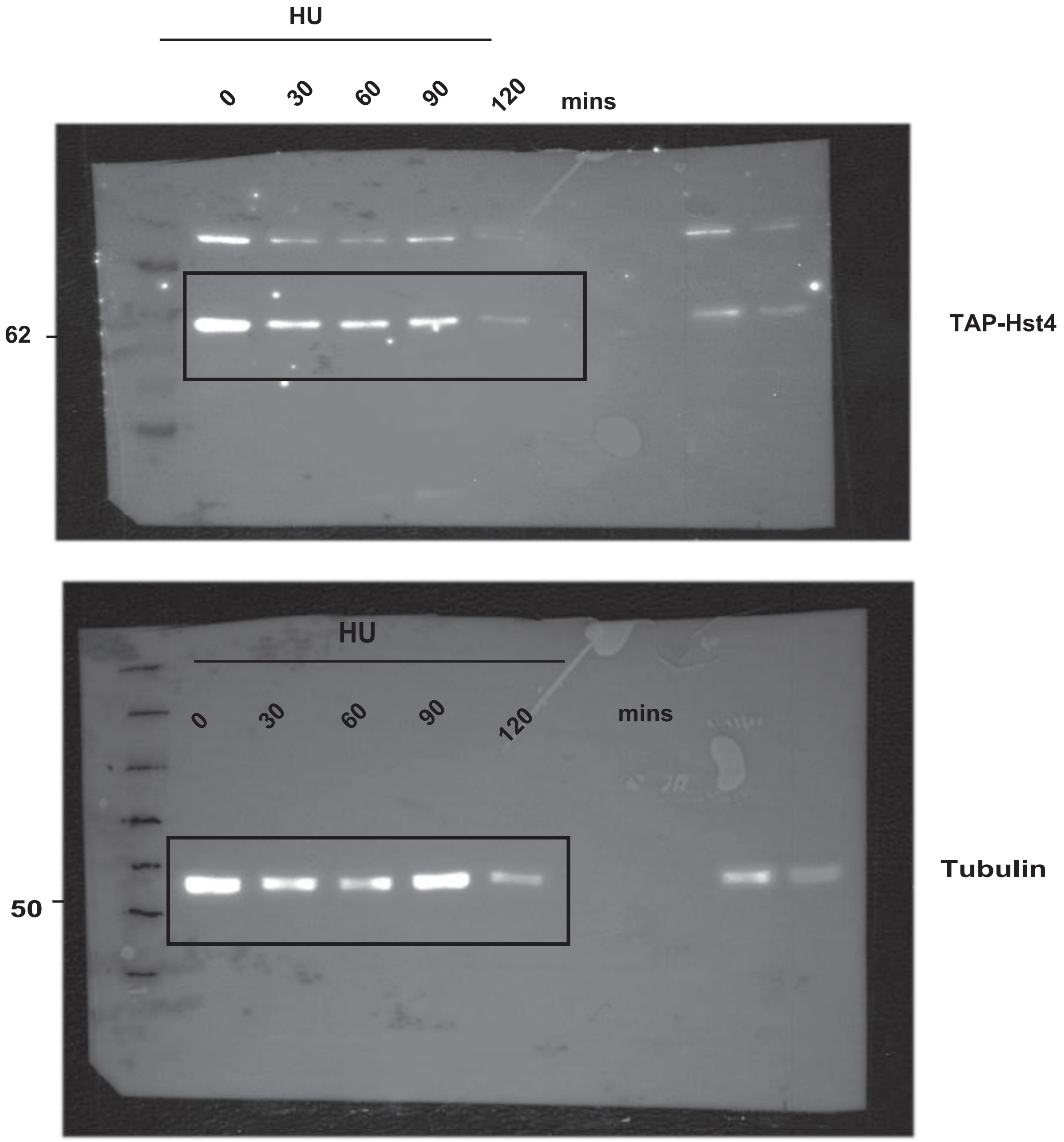

Figure 1-source data 1

Figure 1D

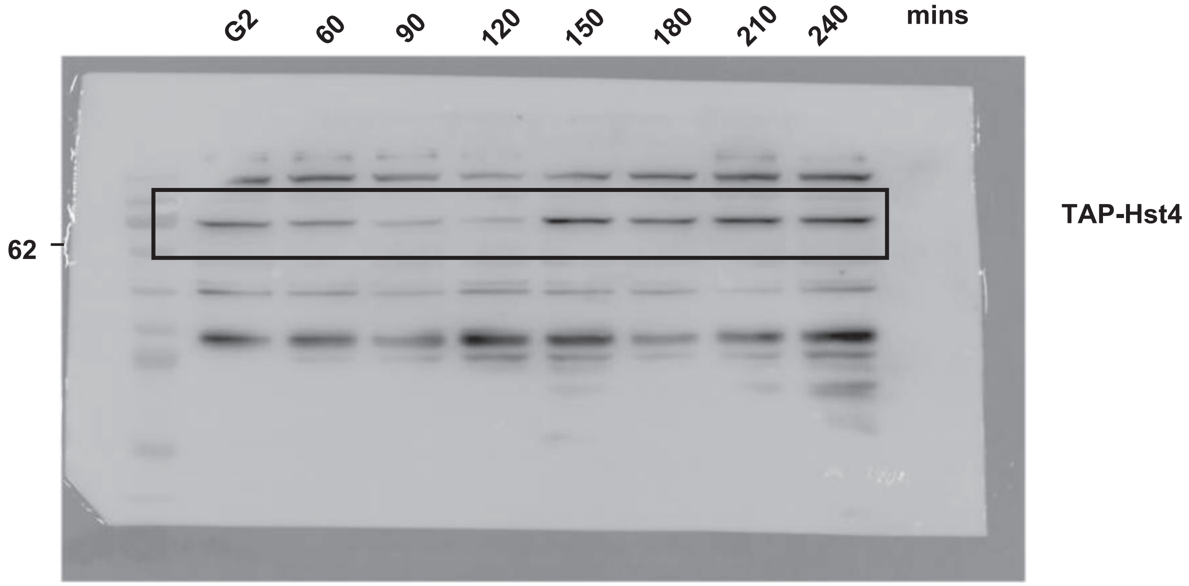

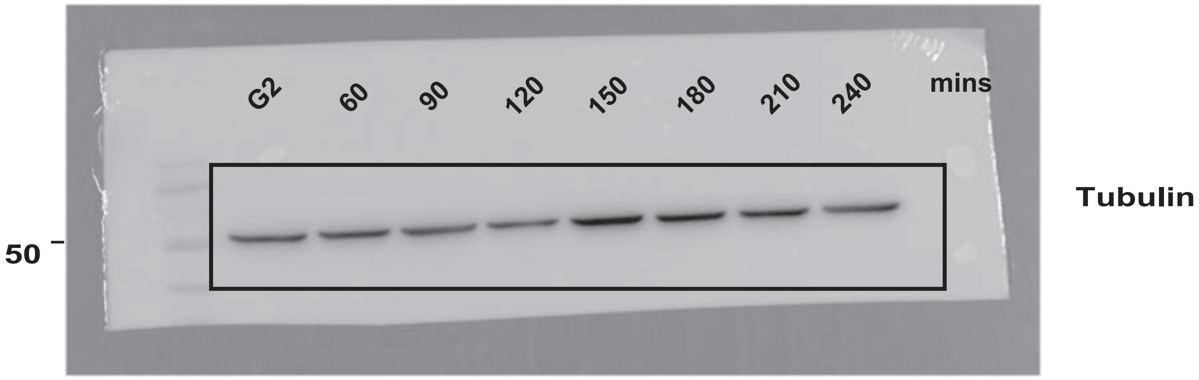

Figure 1-source data 1

Figure 1E

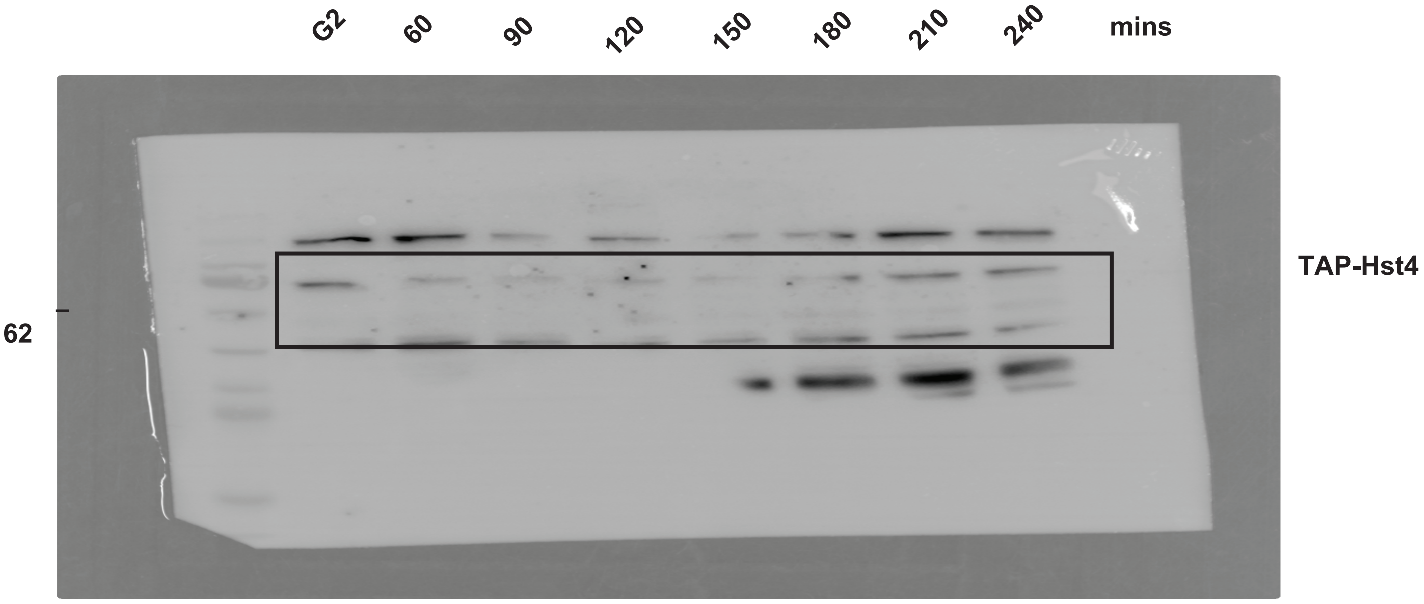

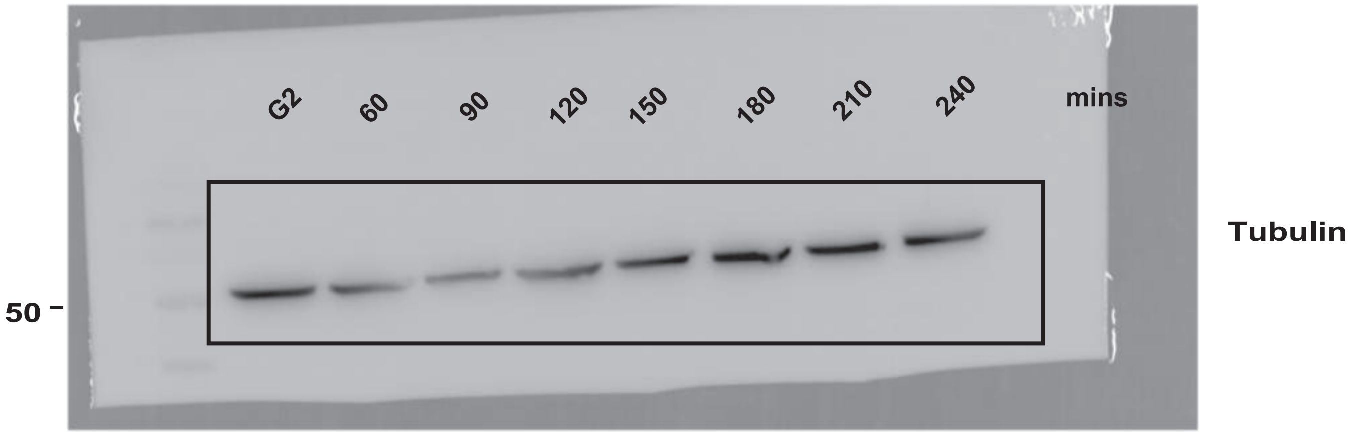

Figure 1-source data 1

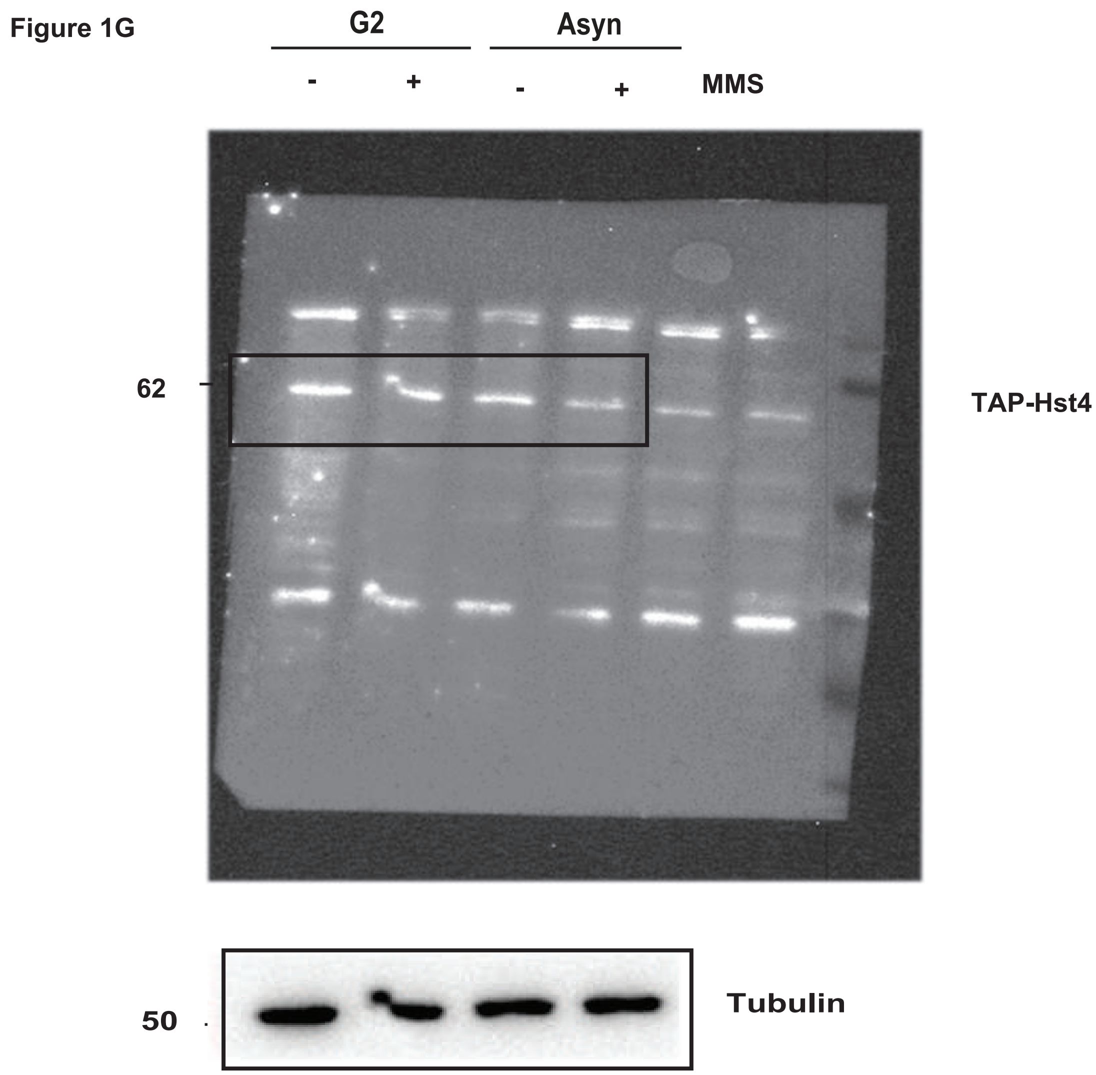

Figure 2-source data 1

Figure 2A

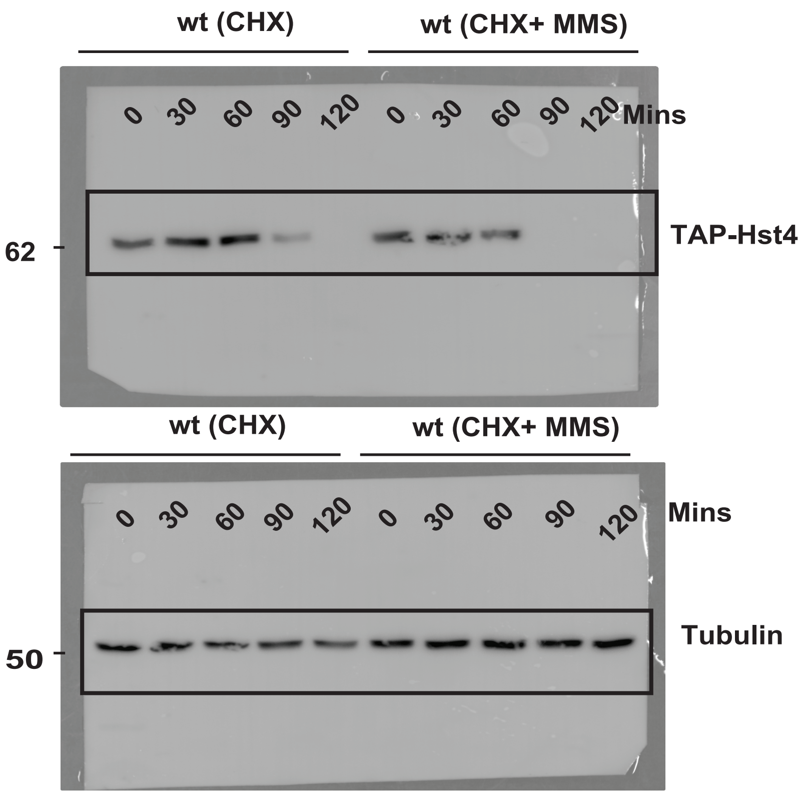

Figure 2-source data 1

Figure 2B

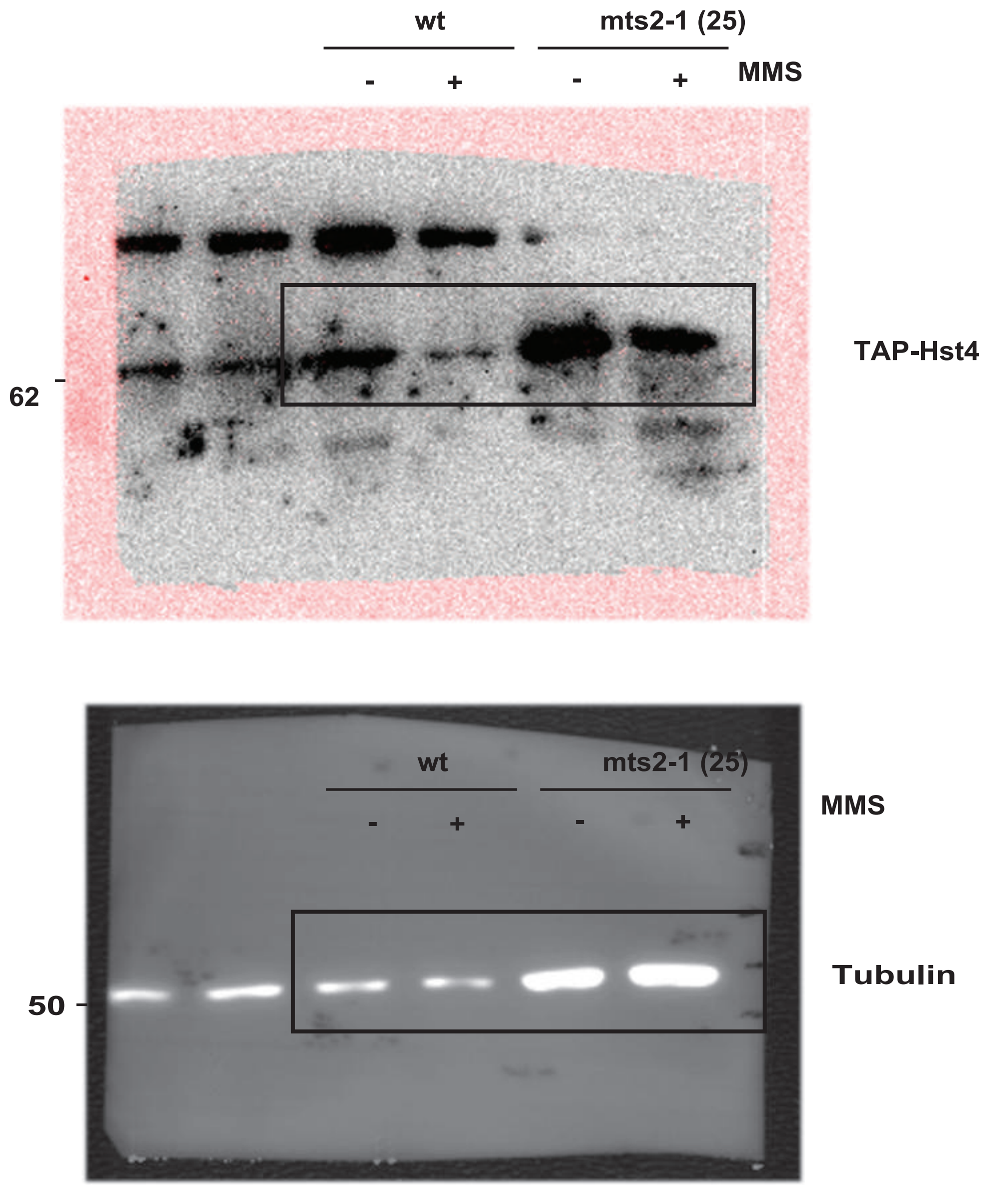

Figure 2-source data 1

Figure 2C

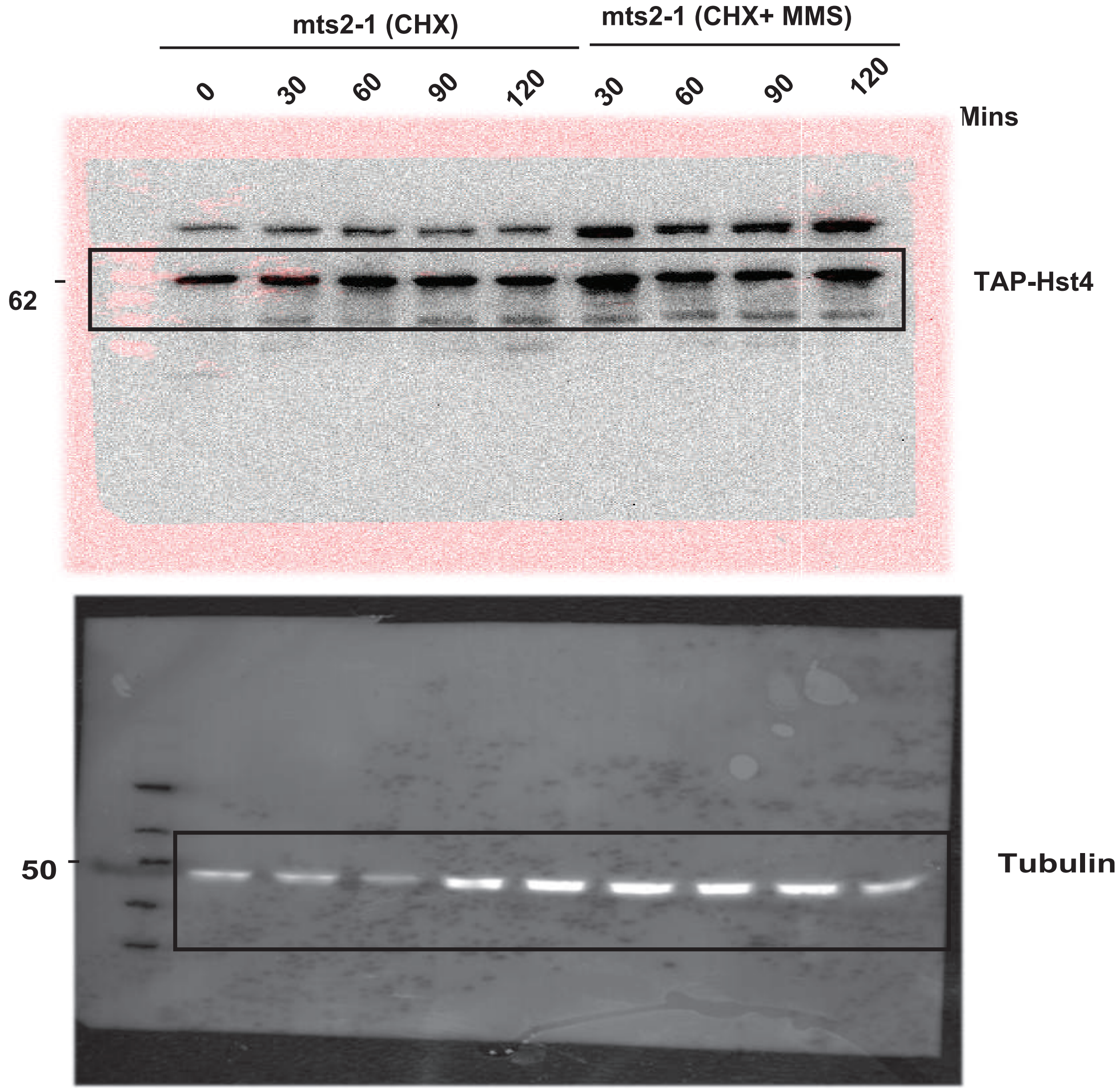

Figure 2-source data 1

Figure 2D

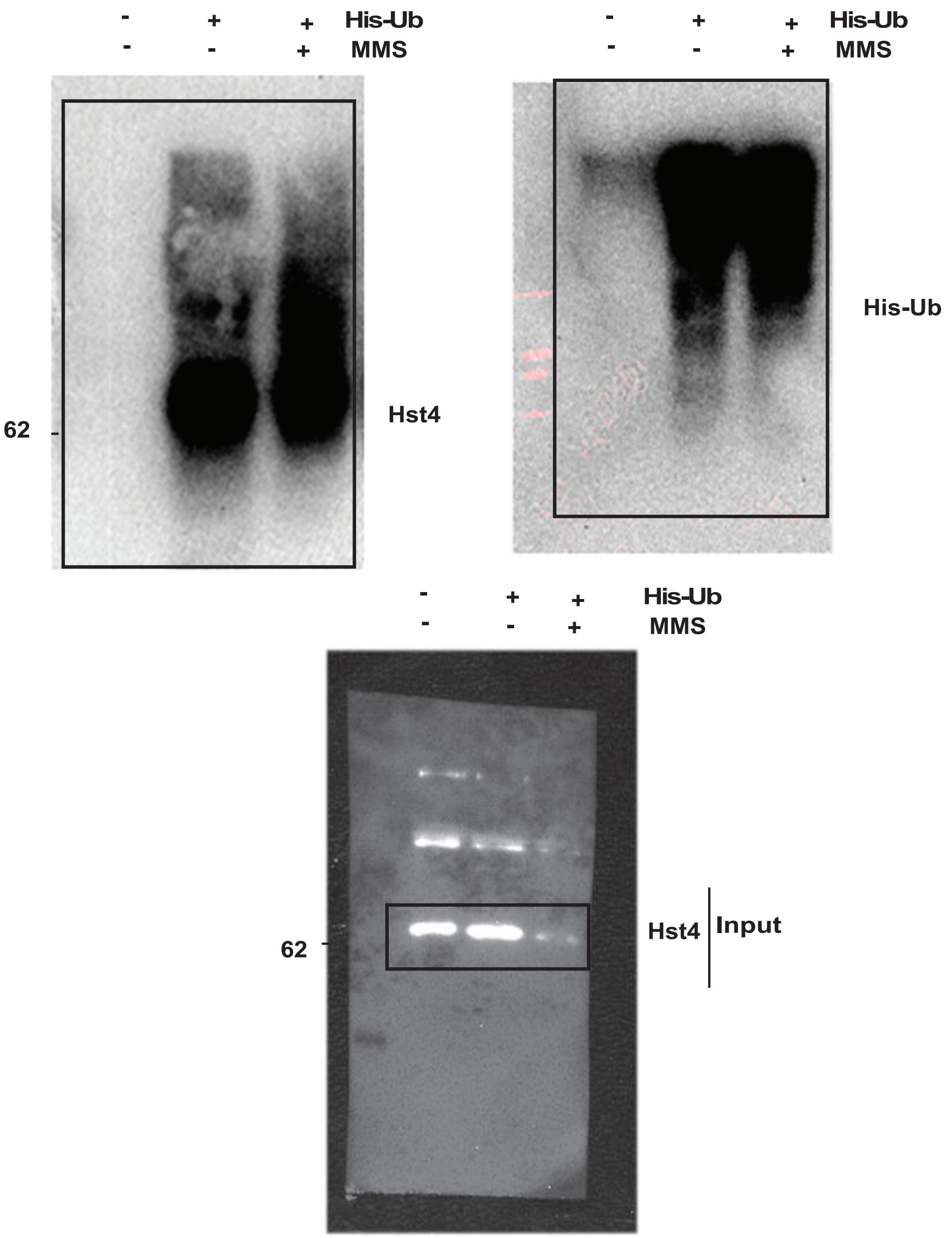

Figure 2-source data 1

Figure 2E

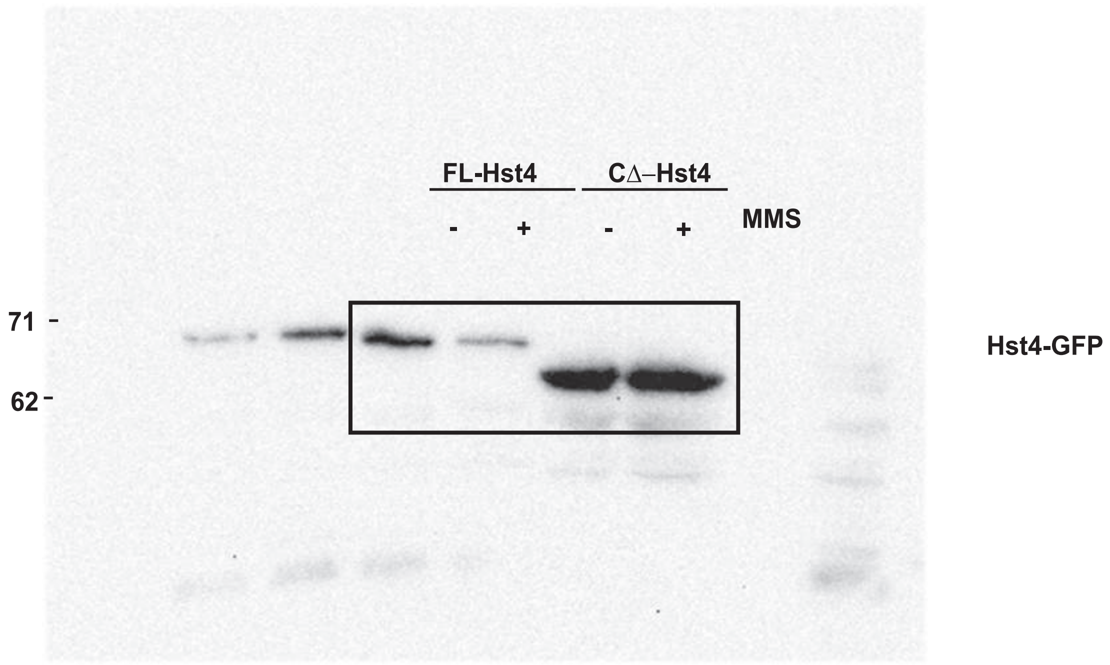

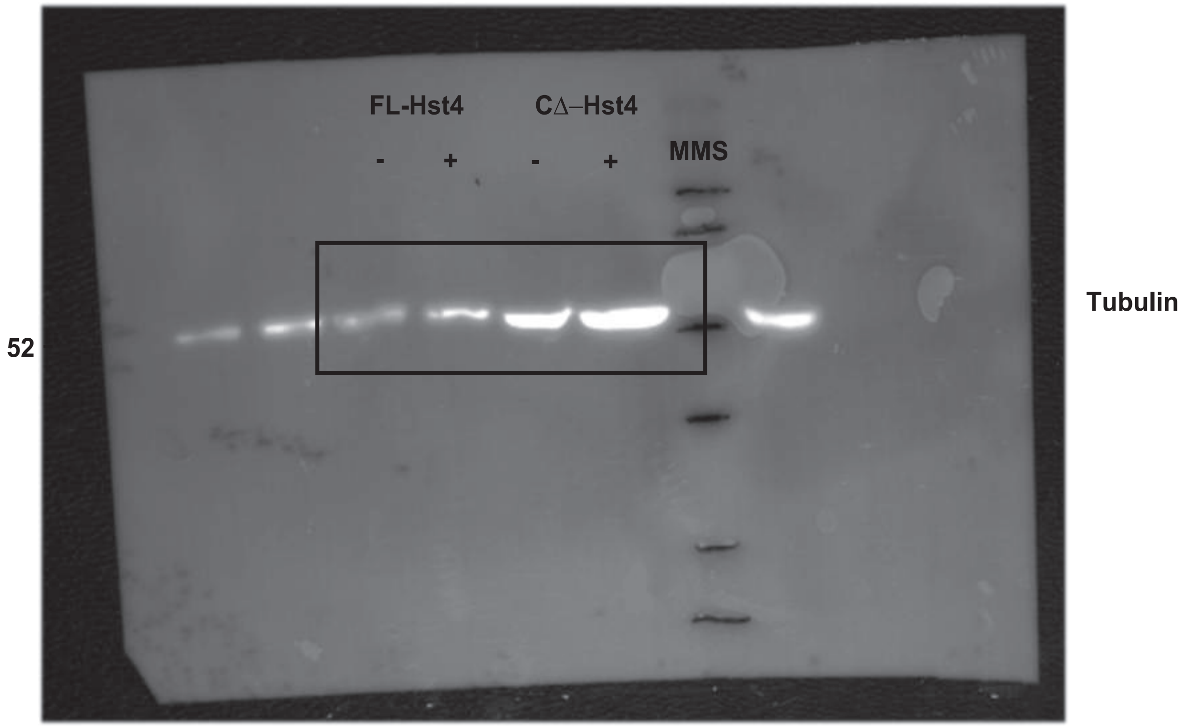

Figure 2-figure supplement 1-source data 1

Figure 2-figure supplement 1D

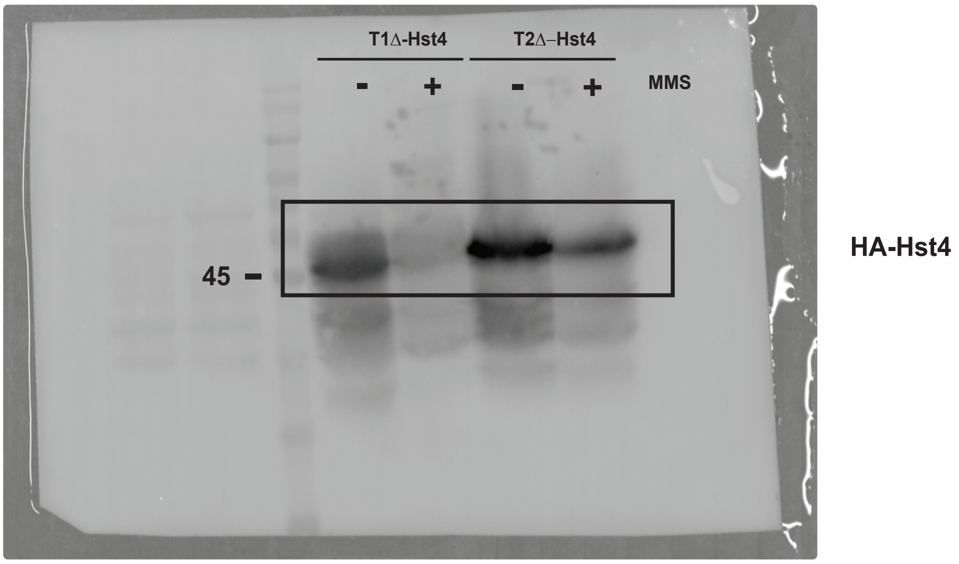

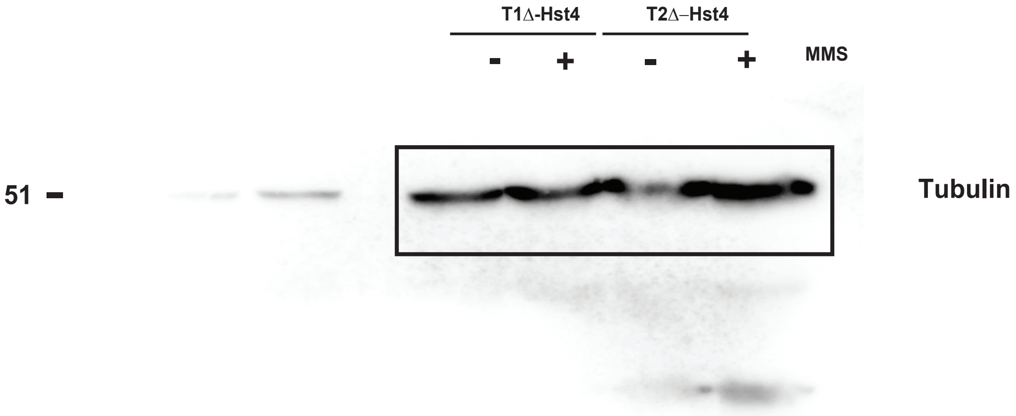

Figure 3-source data 1

Figure 3B

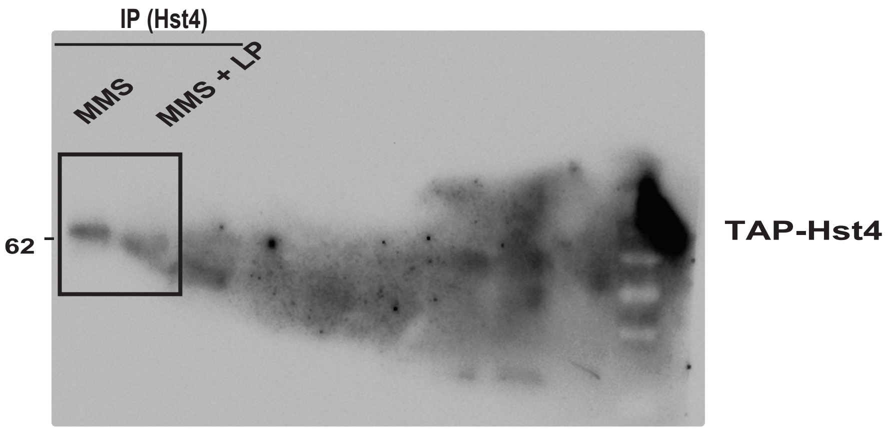

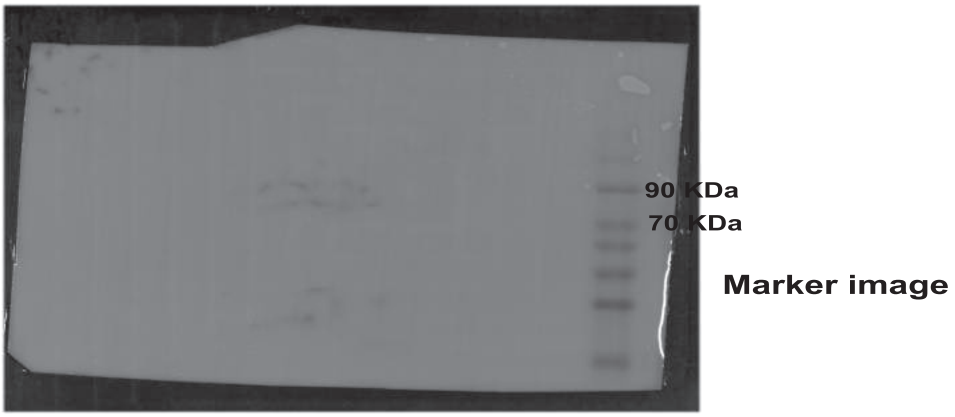

Figure 3-source data 1

Figure 3C

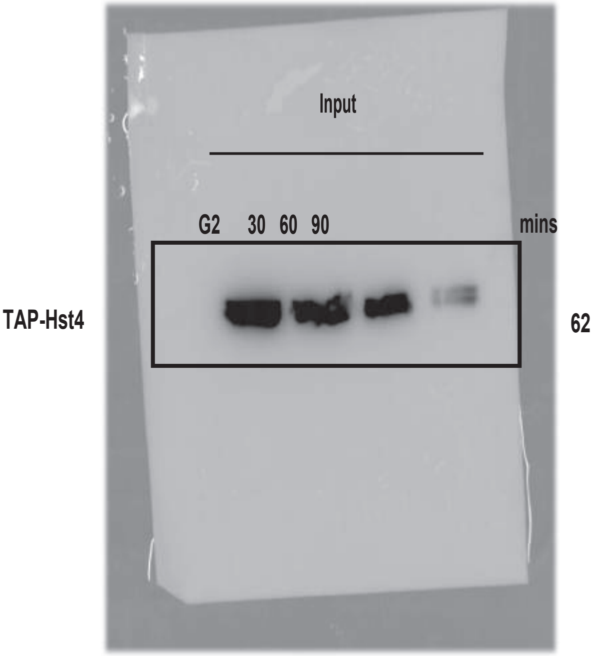

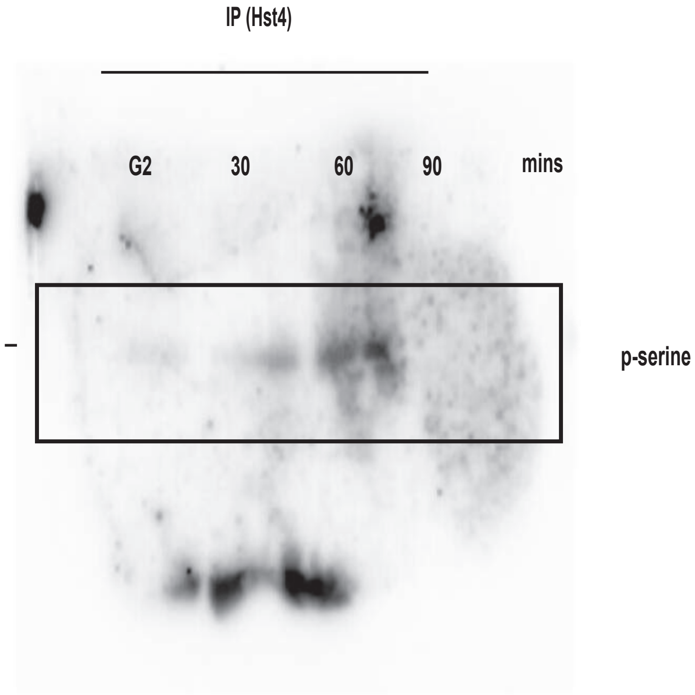

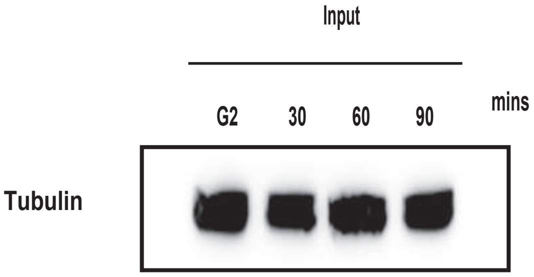

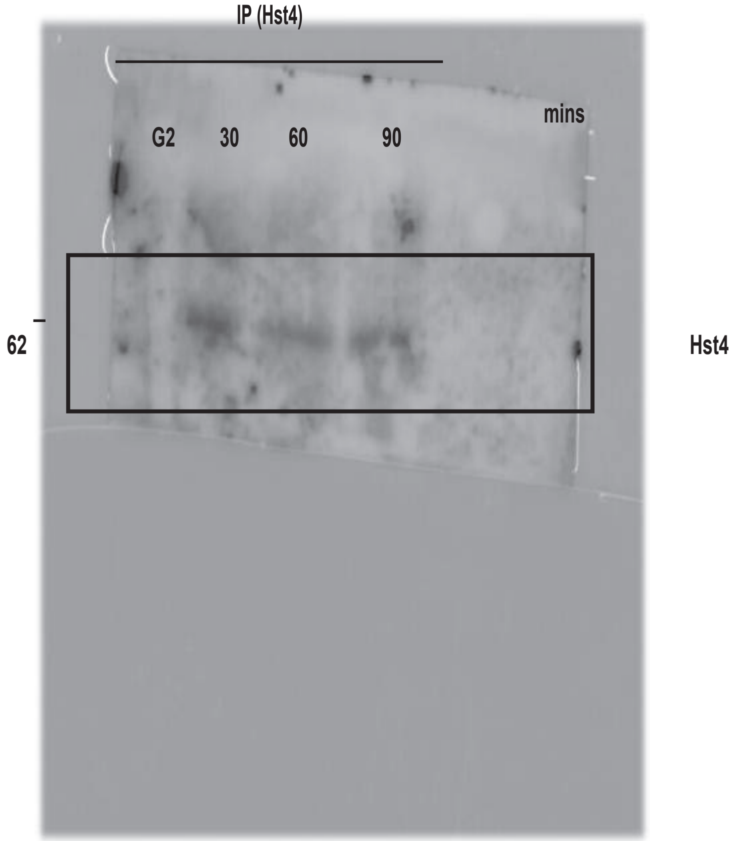

Figure 3-source data 1

Figure 3D

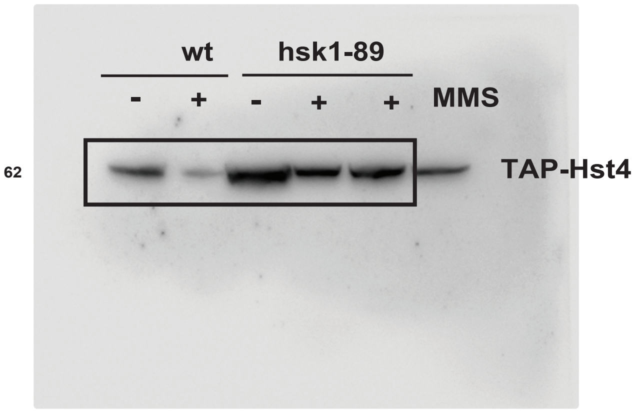

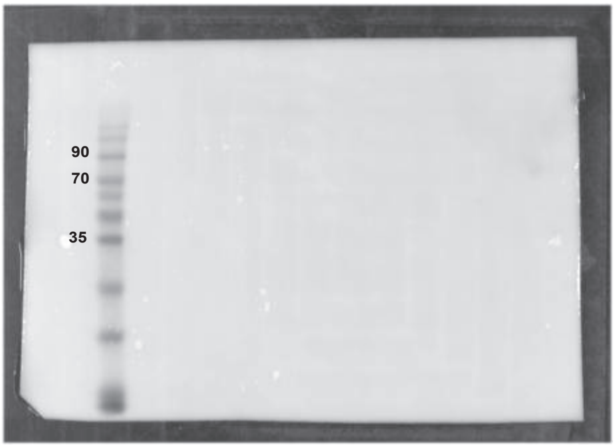

Marker image

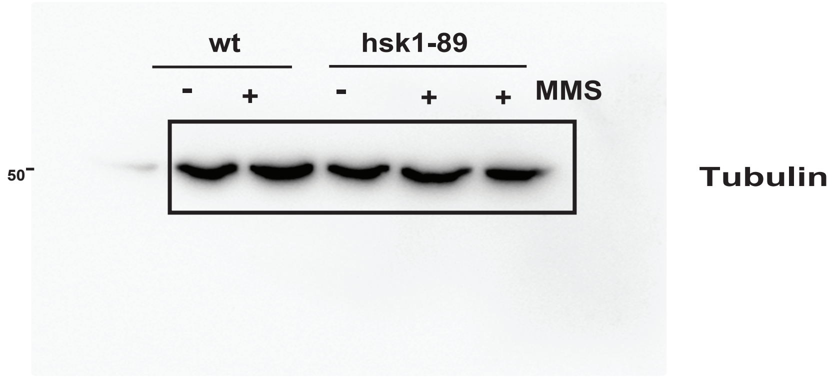

Figure 3-source data 1

Figure 3E

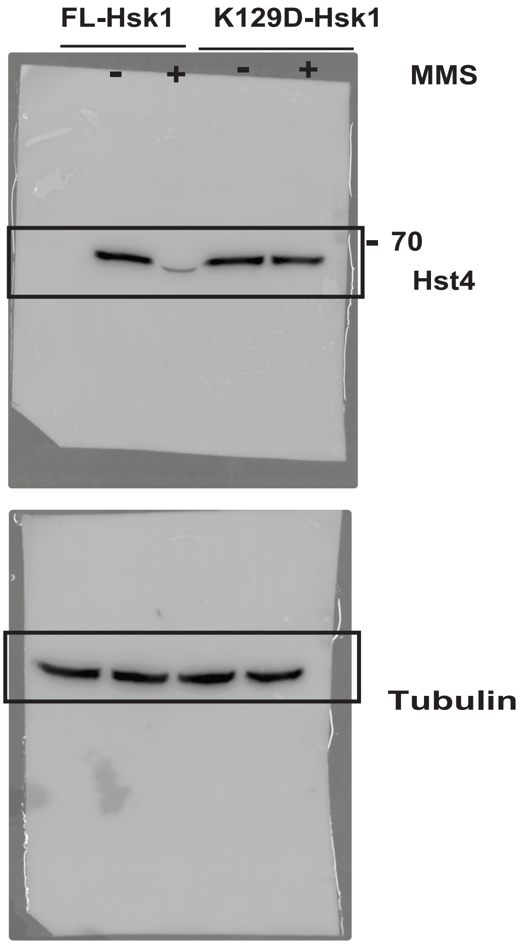

Figure 3-source data 1

Figure 3F

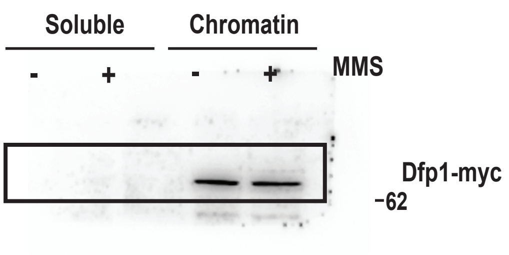

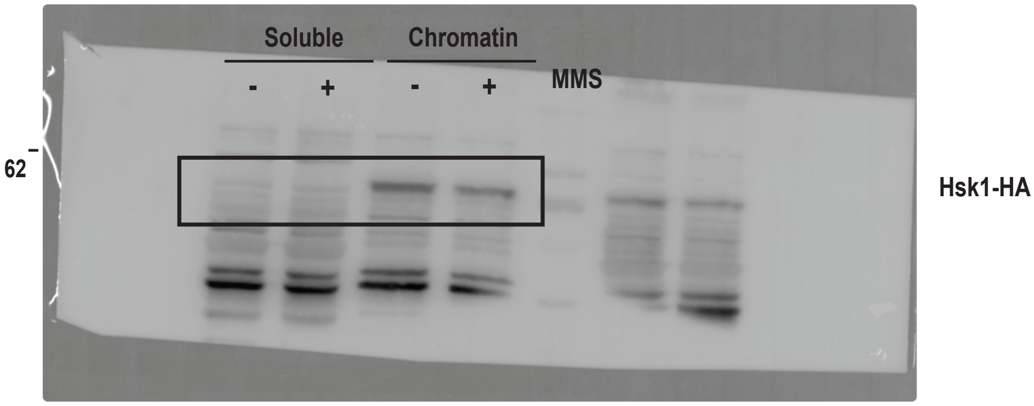

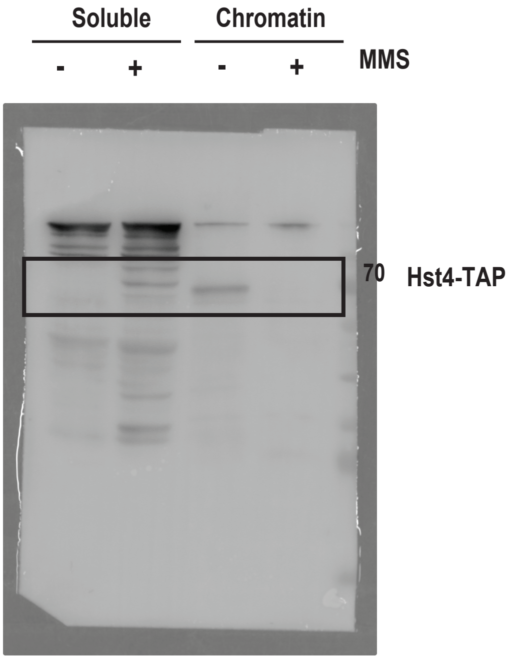

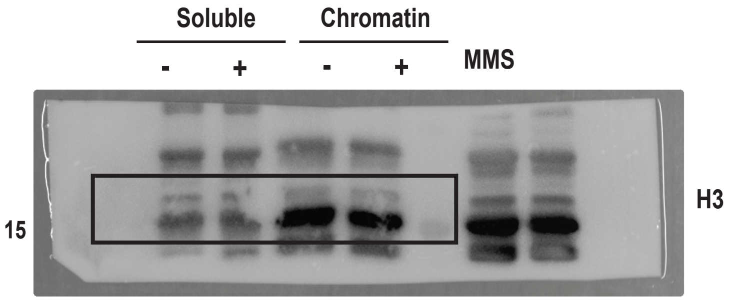

Figure 3-source data 1

Figure 3G

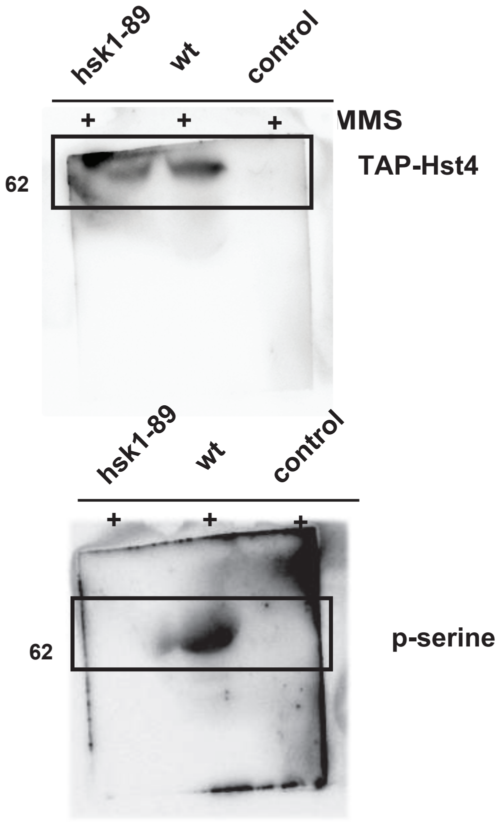

Figure 3-source data 1

Figure 3H

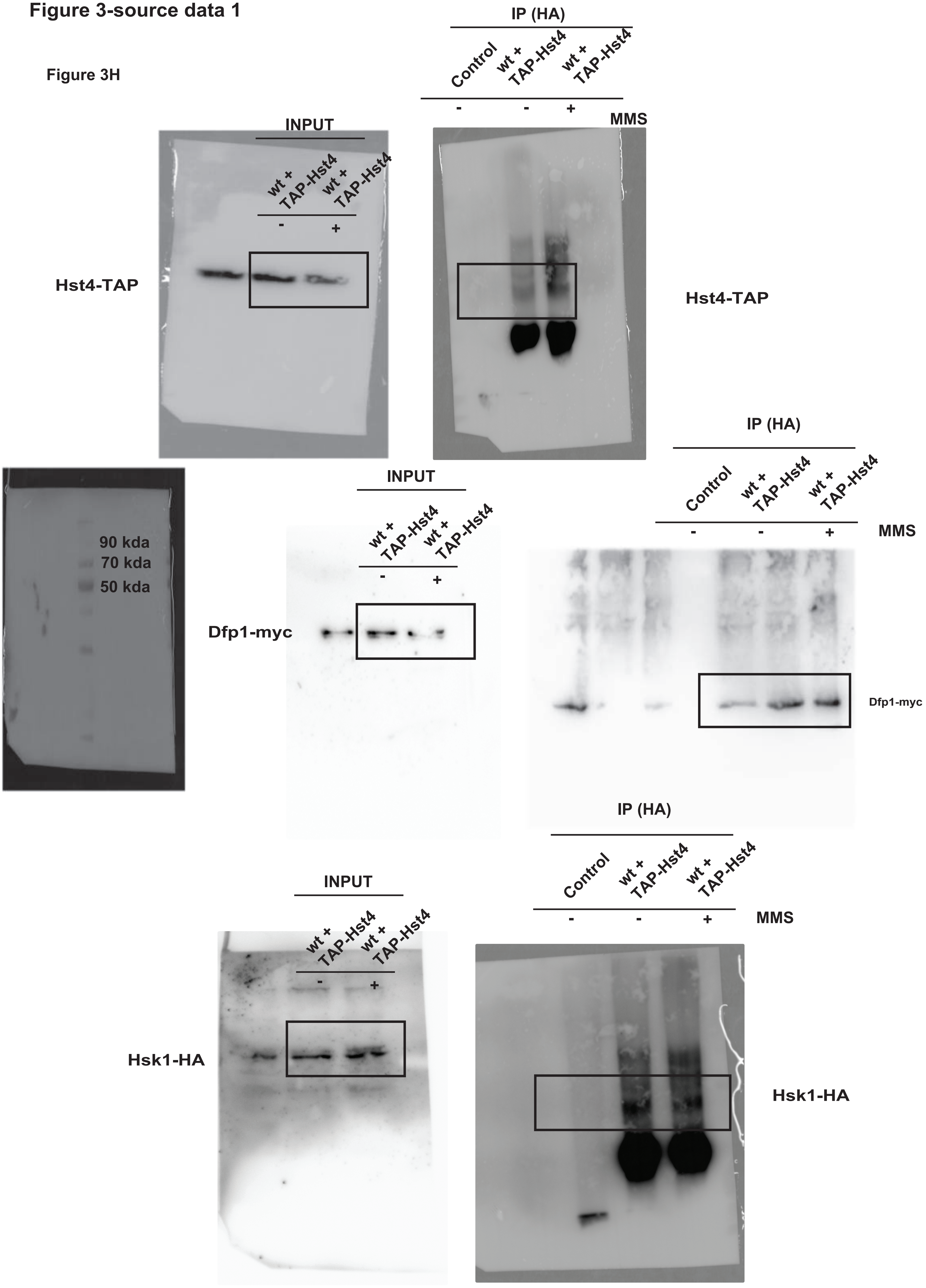

Figure 3-source data 1

Figure 3I

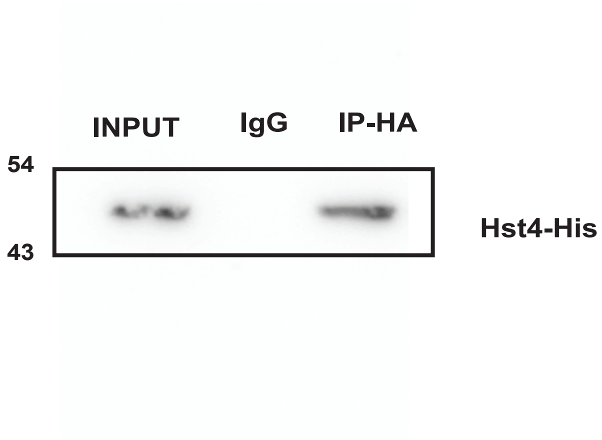

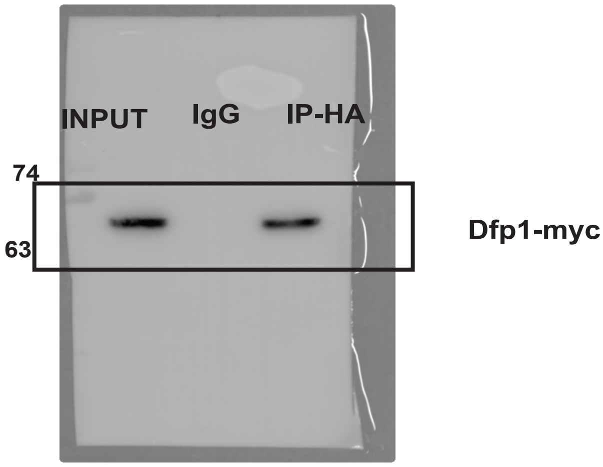

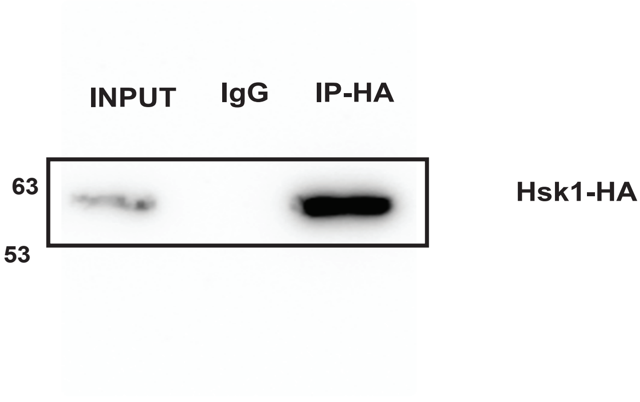

Figure 3-source data 1

Figure 3J

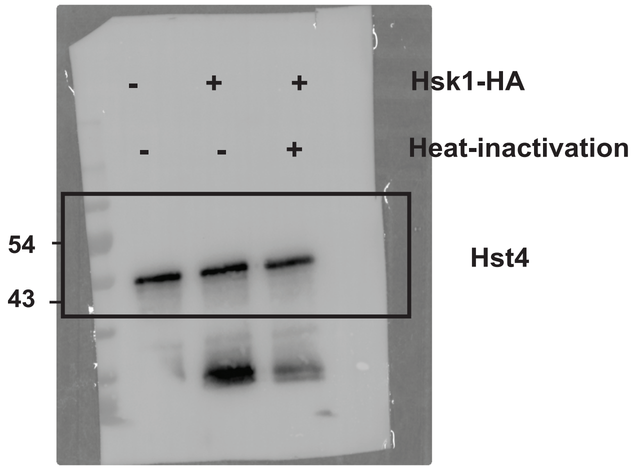

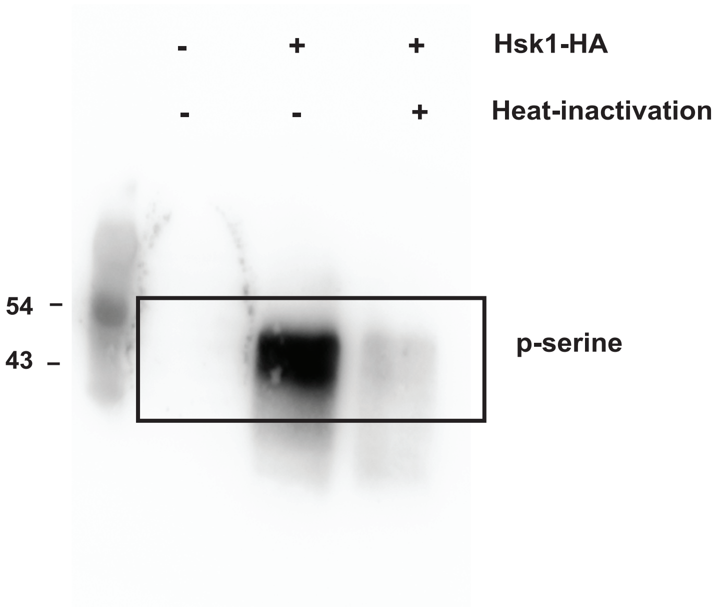

Figure 3-figure supplement 1-source data 1

Figure 3-Figure Supplement 1A

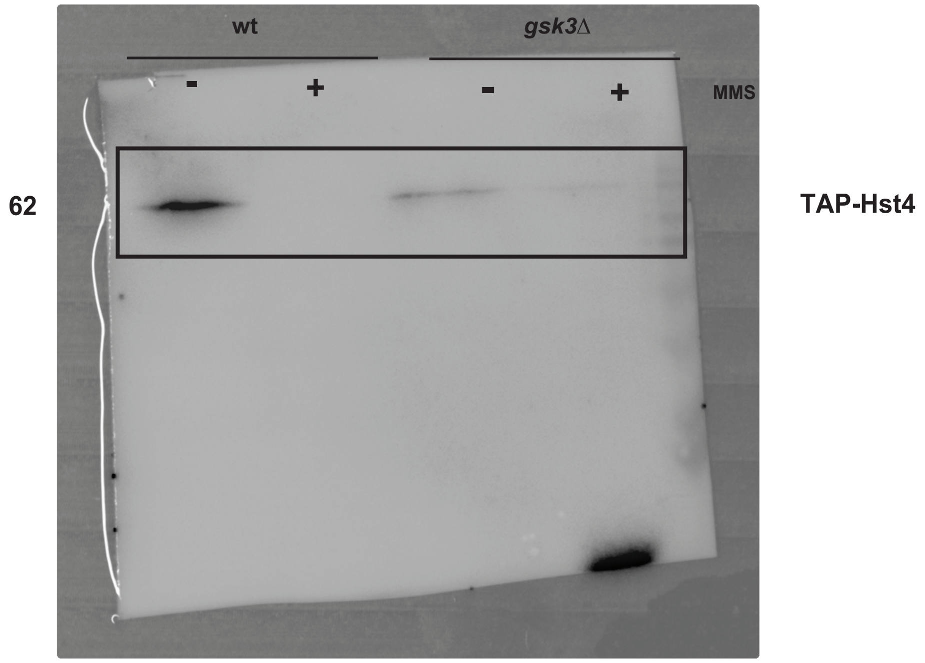

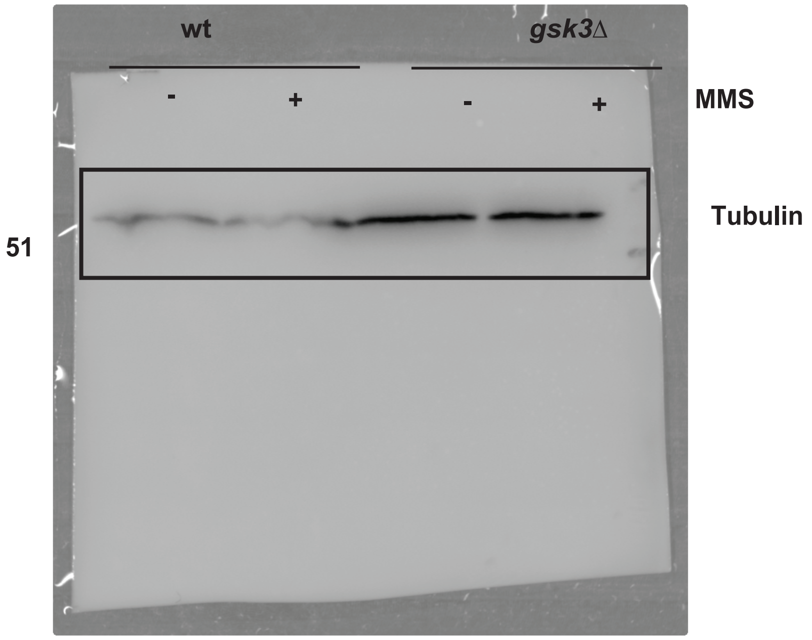

Figure 3-figure supplement 1-source data 1

Figure 3-Figure Supplement 1B

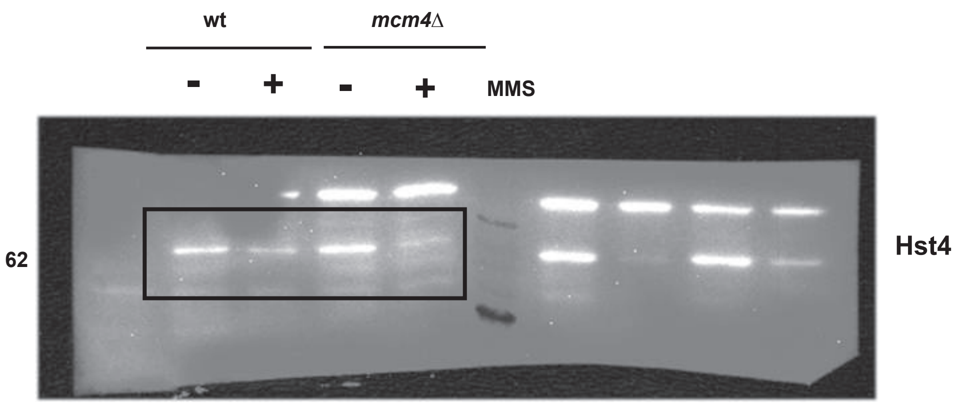

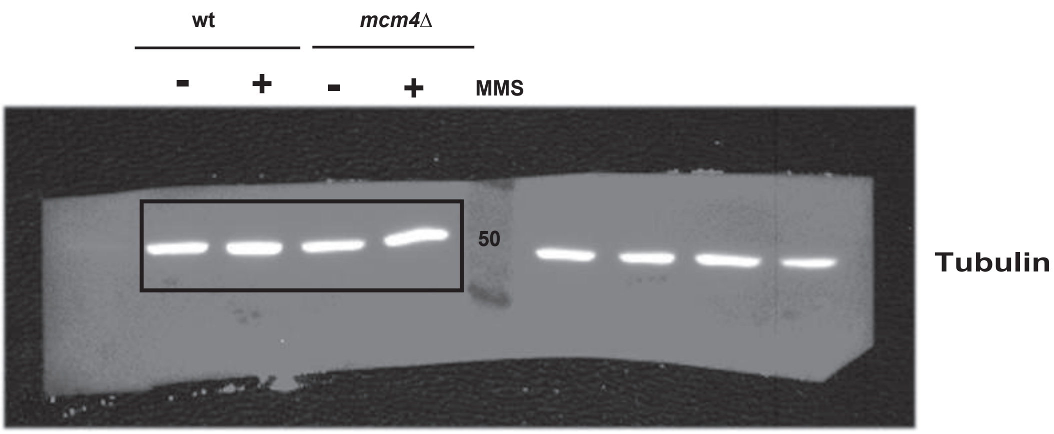

Figure 3-figure supplement 1-source data 1

Figure 3-Figure Supplement 1C

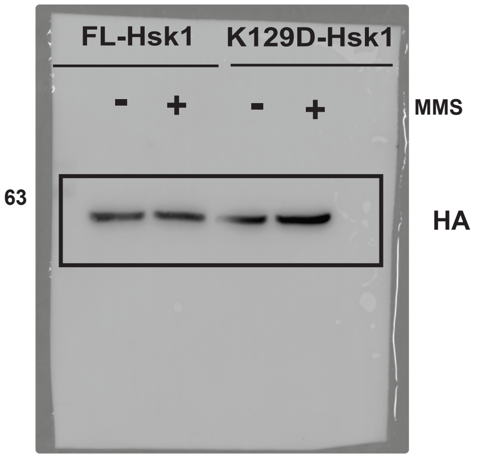

Figure 4-source data 1

Figure 4C

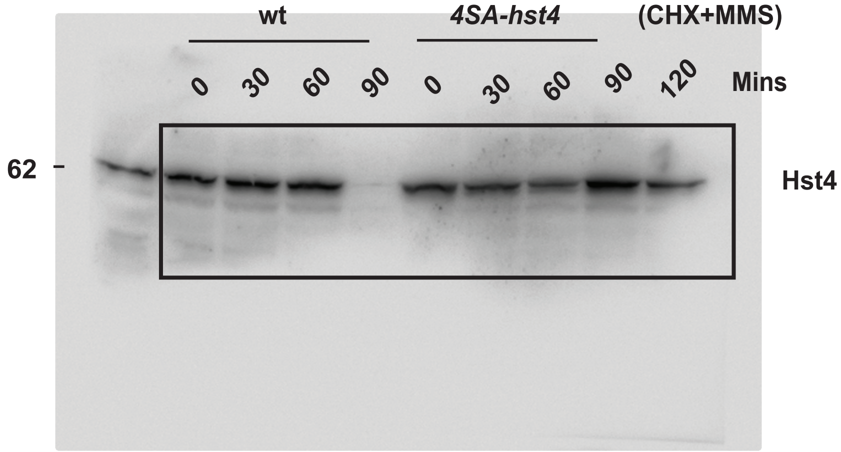

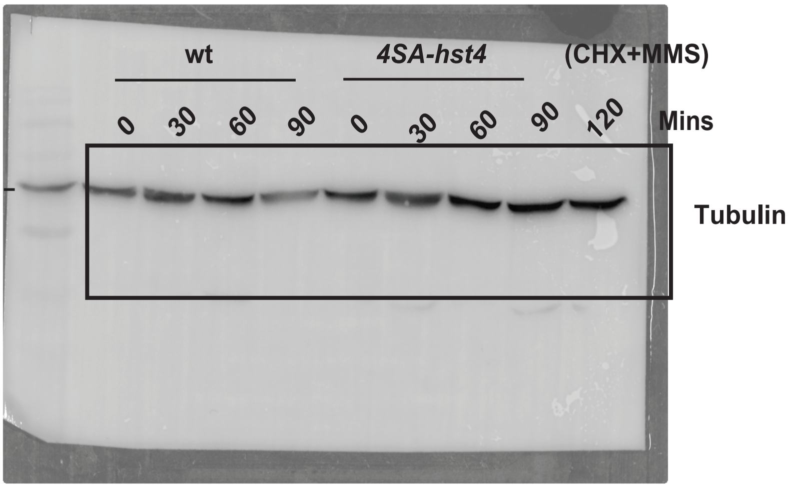

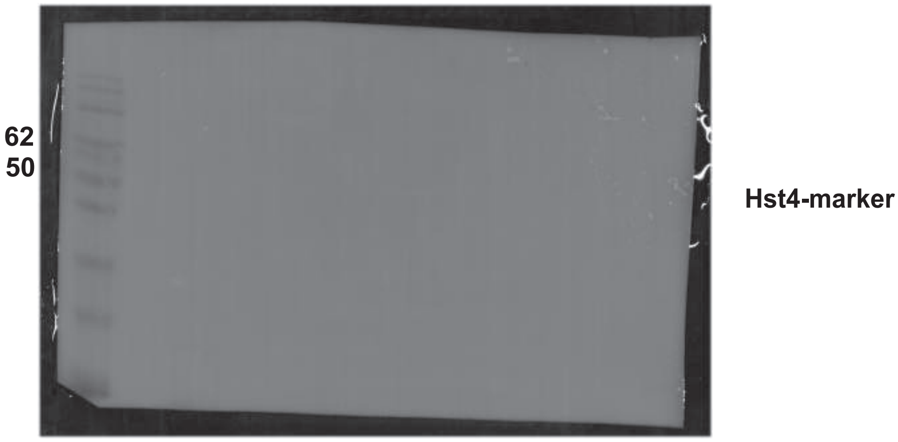

Figure 4-source data 1

Figure 4D

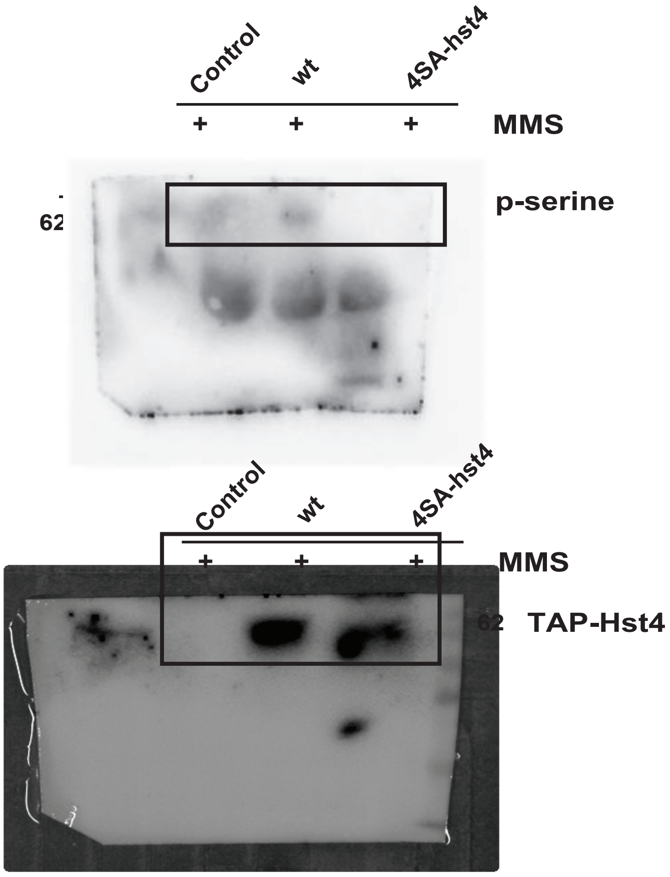

Figure 4-source data 1

Figure 4E

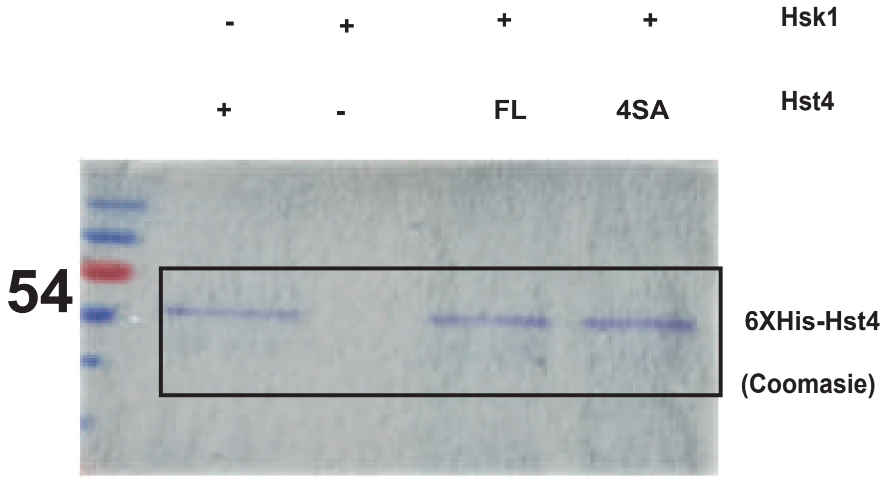

Figure 4-figure supplement 1-source data 1

Figure 4-figure supplement 1A

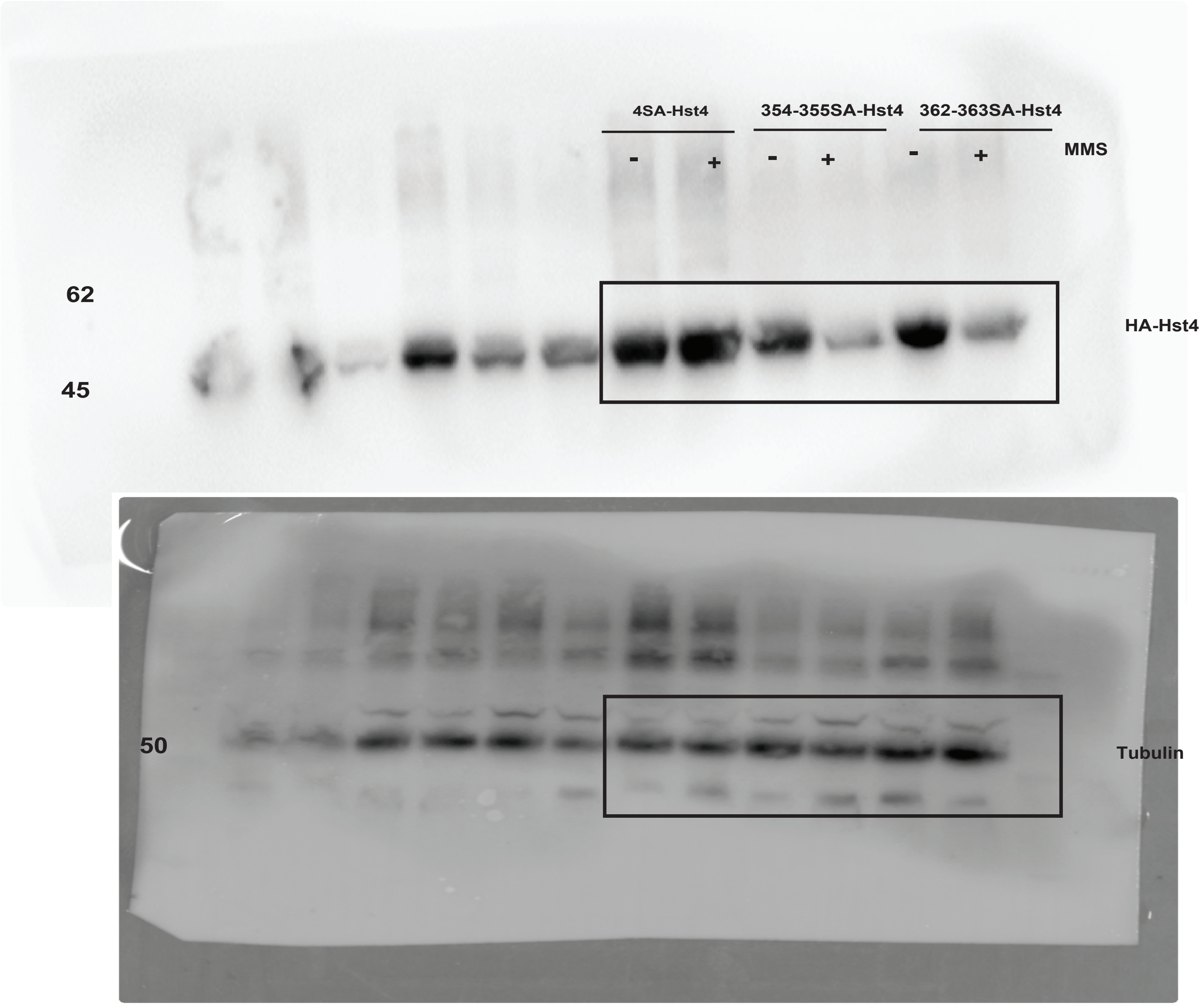

Figure 4-supplemental 1-source data 1

Figure 4-figure supplement 1B

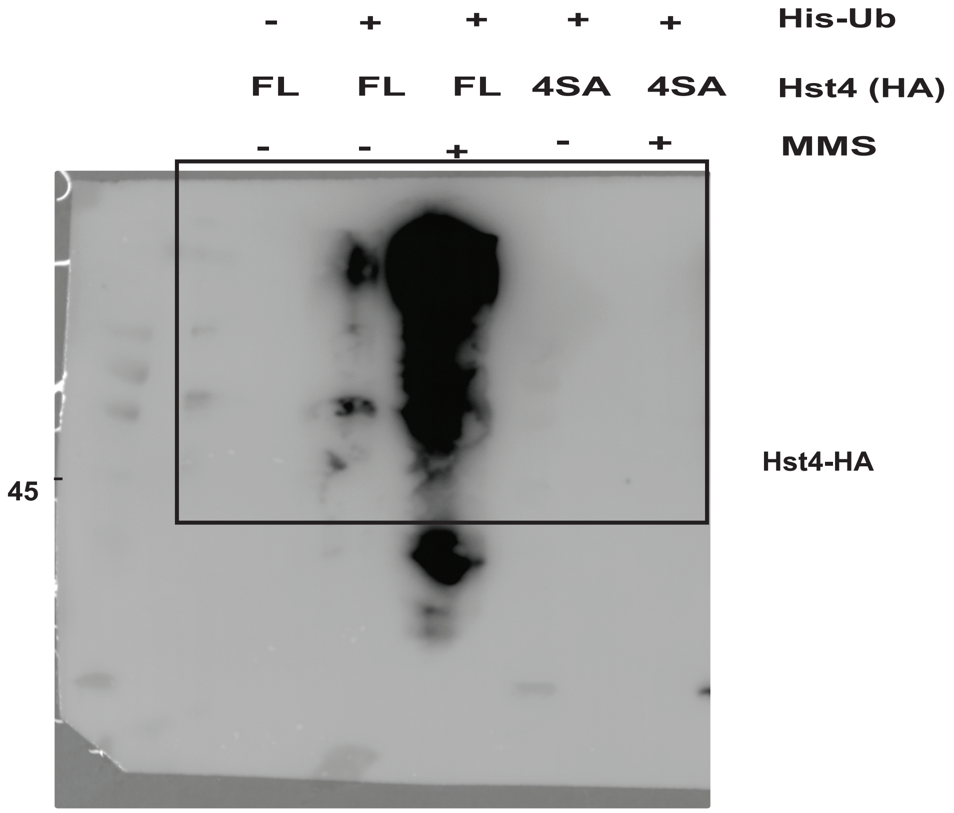

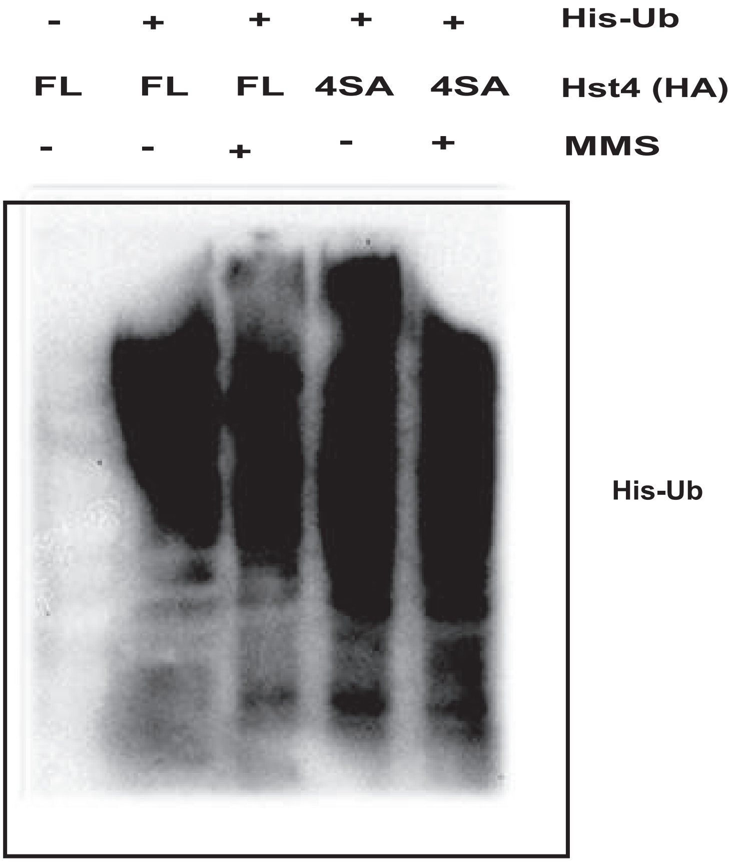

Figure 5-source data 1

Figure 5A

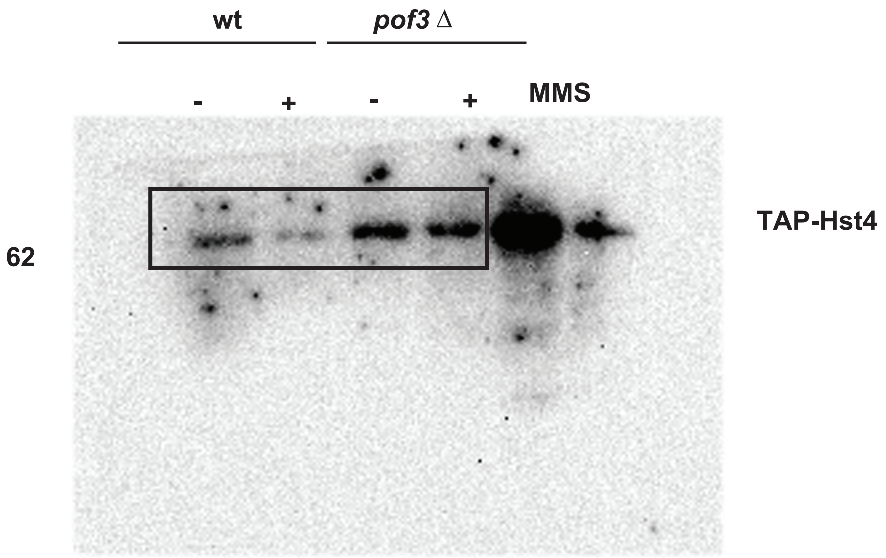

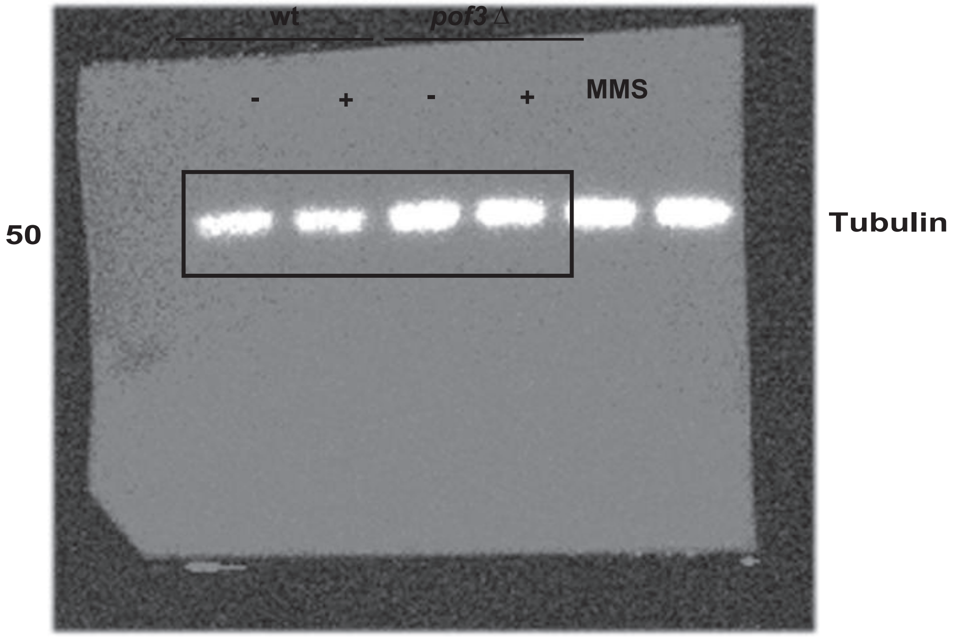

Figure 5-source data 1

Figure 5B

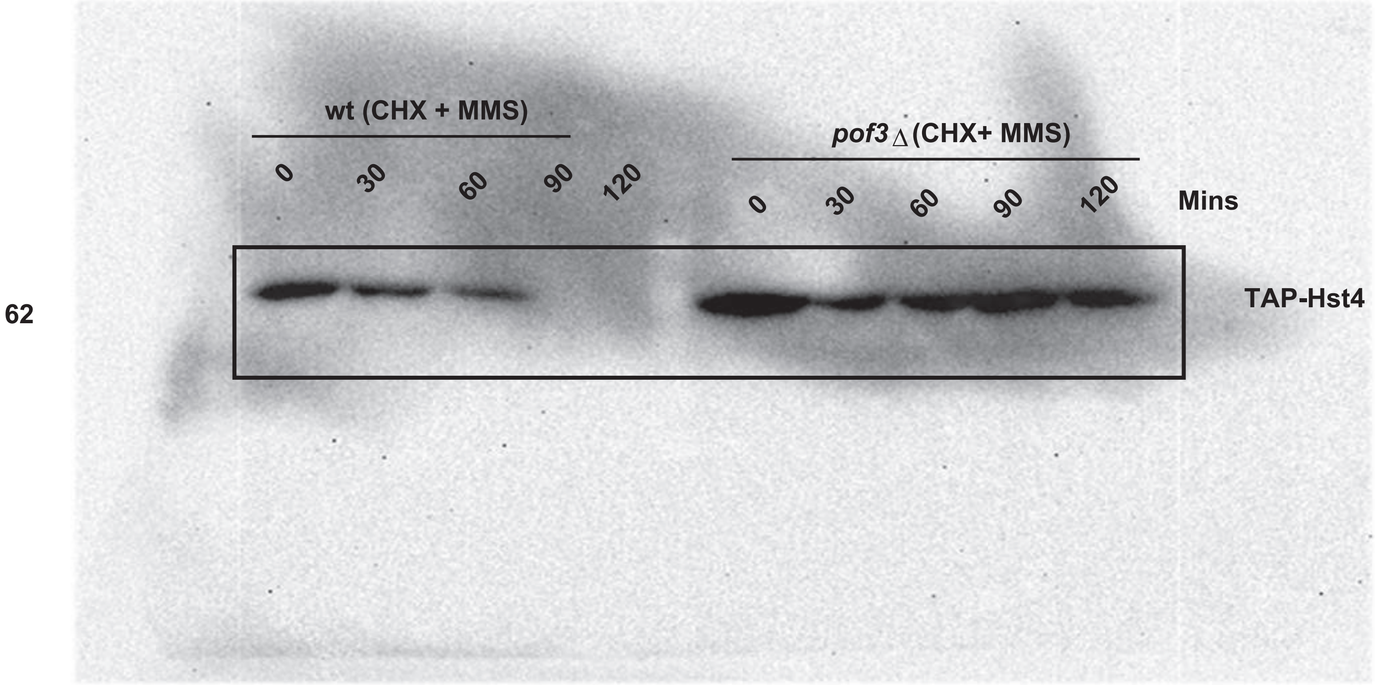

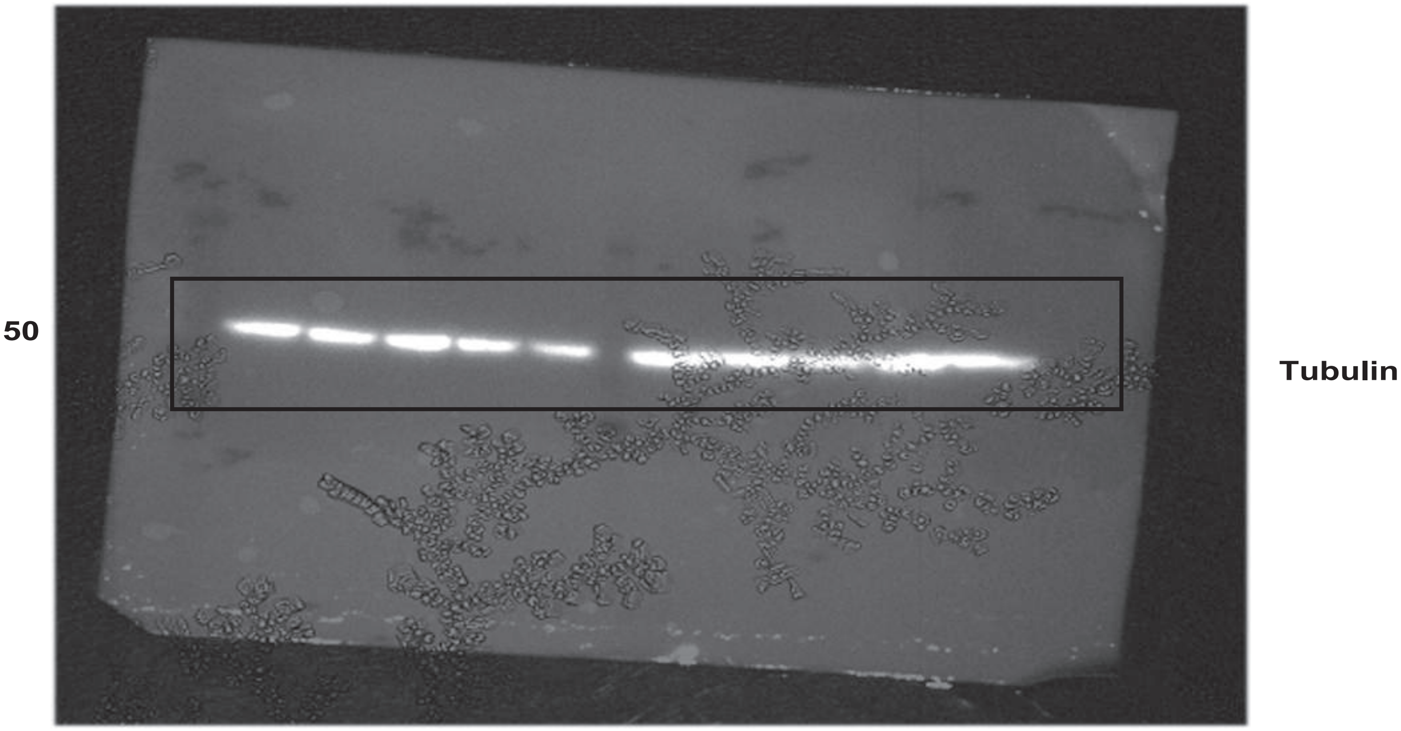

Figure 5-source data 1

Figure 5C

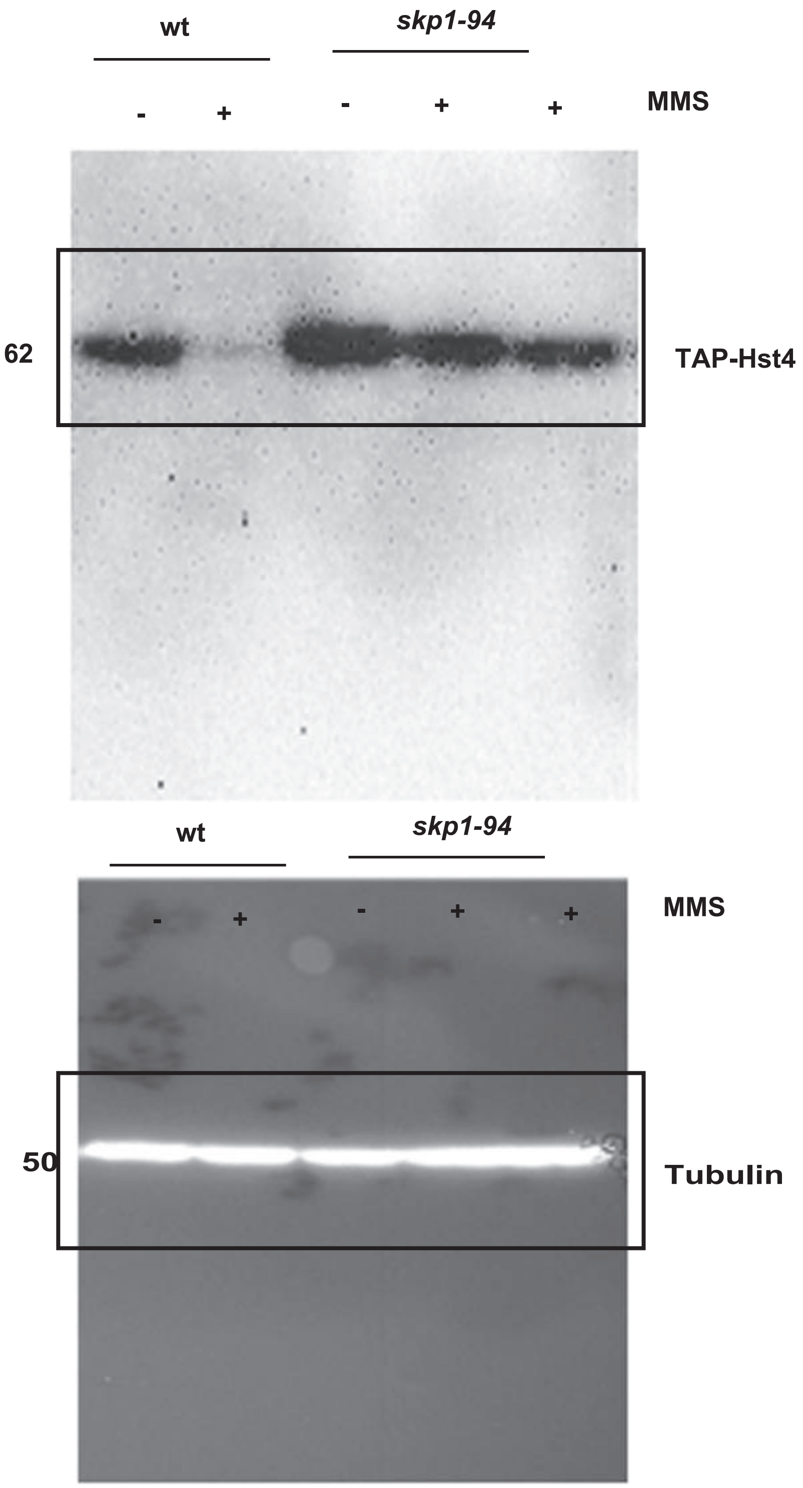

Figure 5-source data 1

Figure 5E

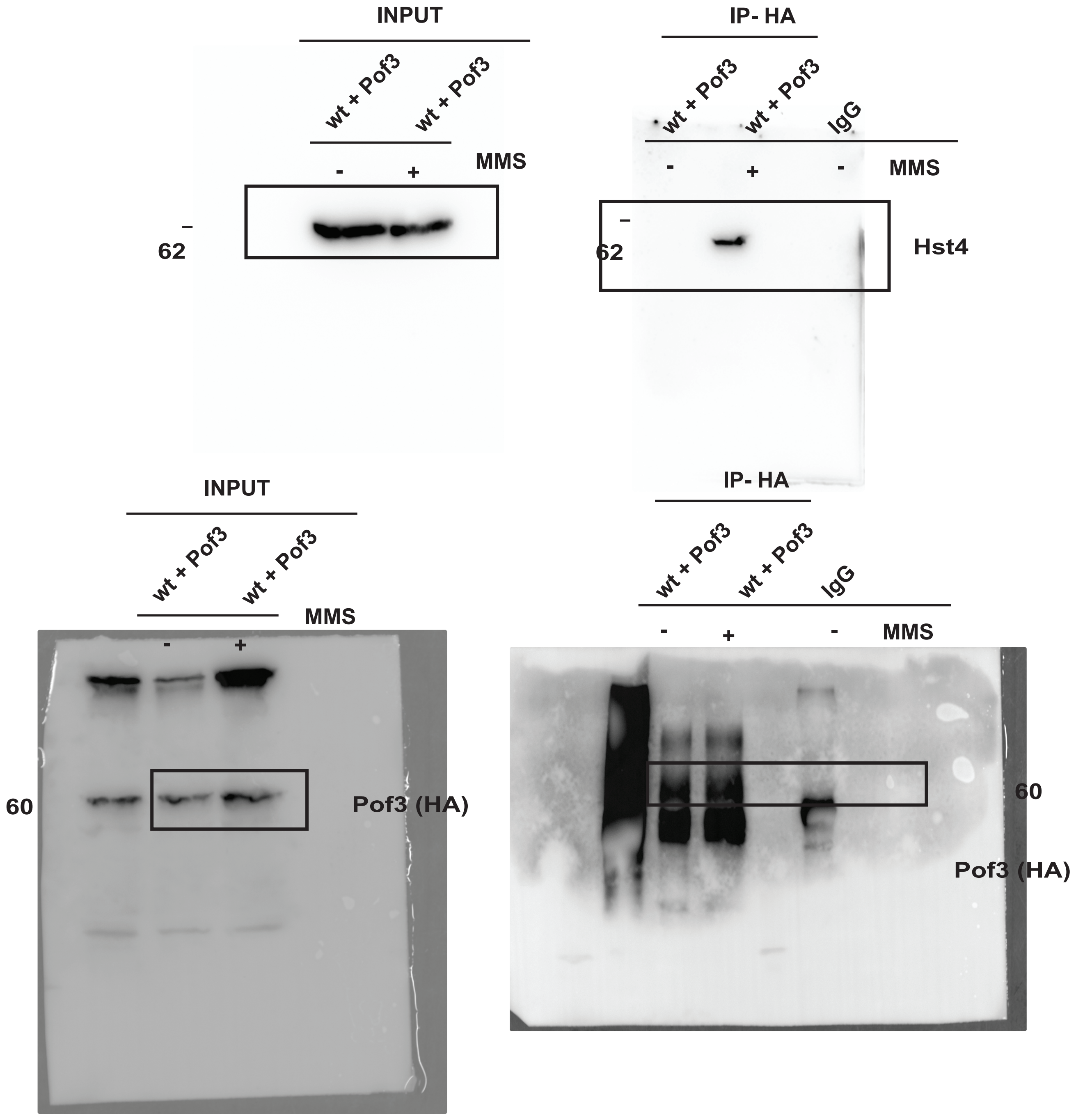

Figure 5-figure supplement 1-source data 1

Figure 5-Figure Supplement 1C

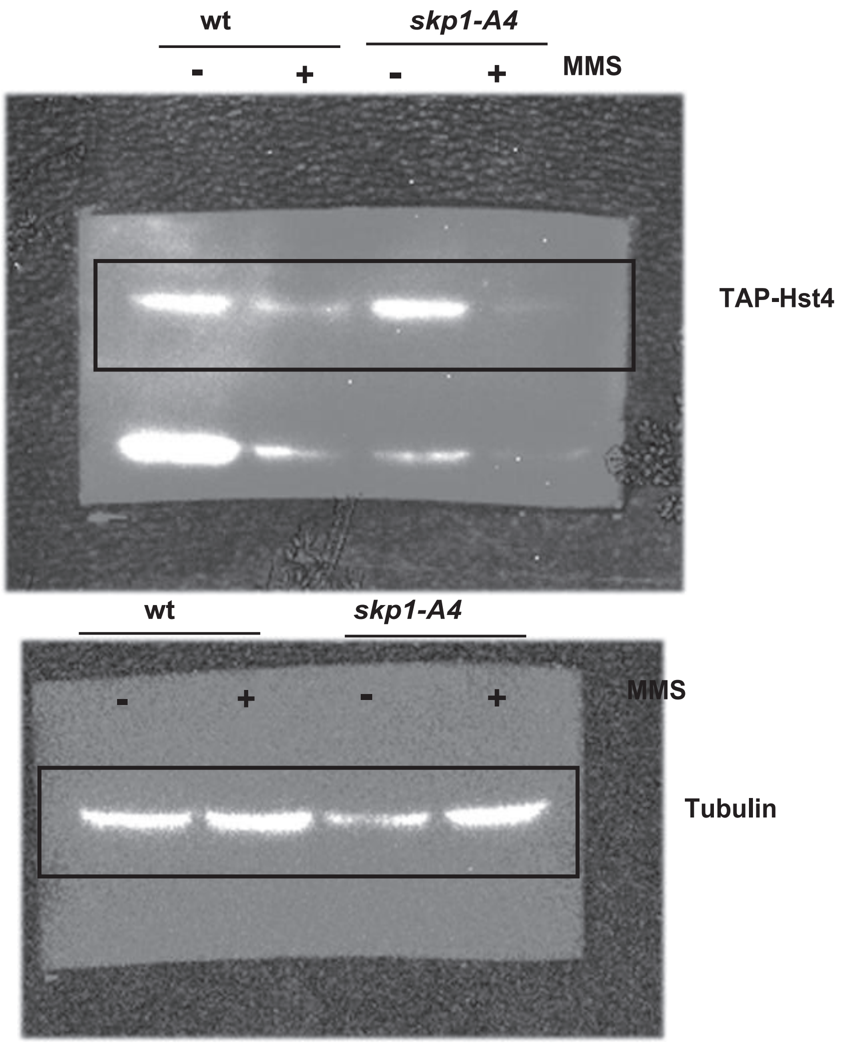

Figure 6-source data 1

Figure 6C

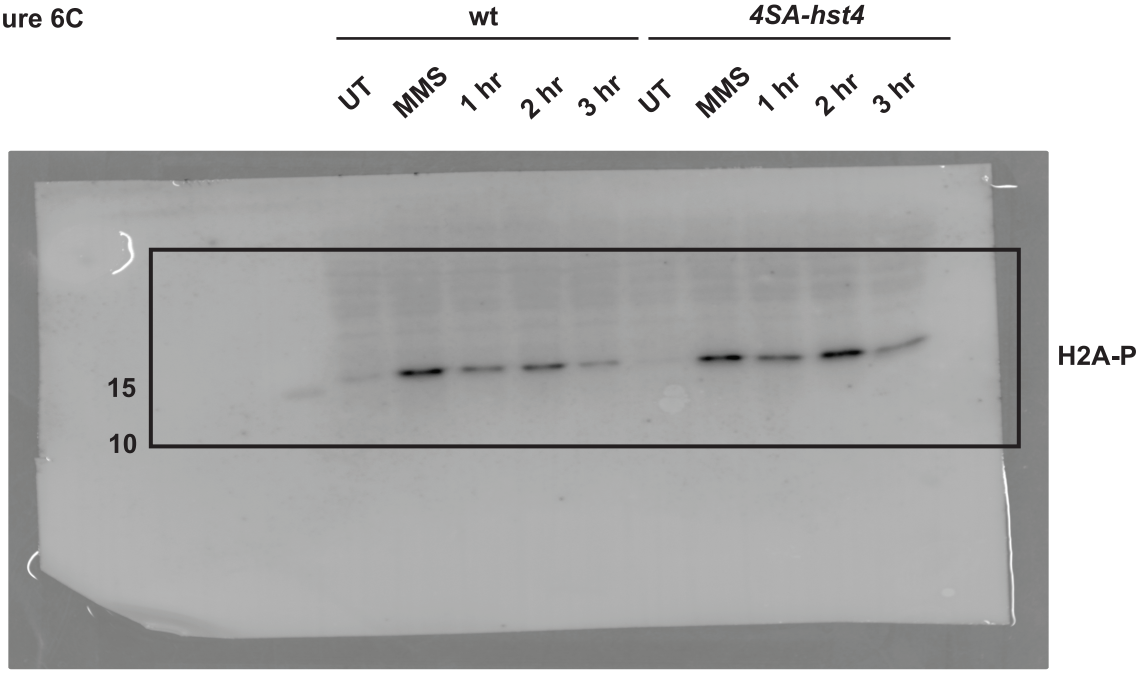

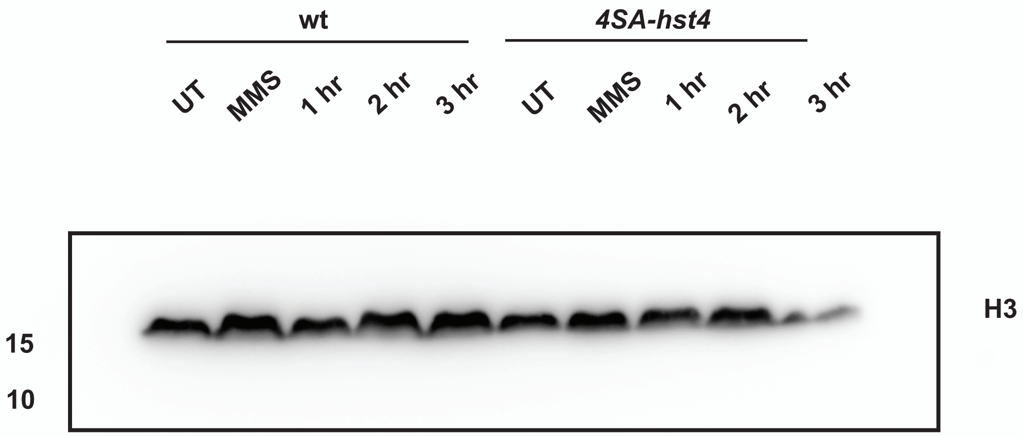

Figure 7-source data 1

Figure 7B

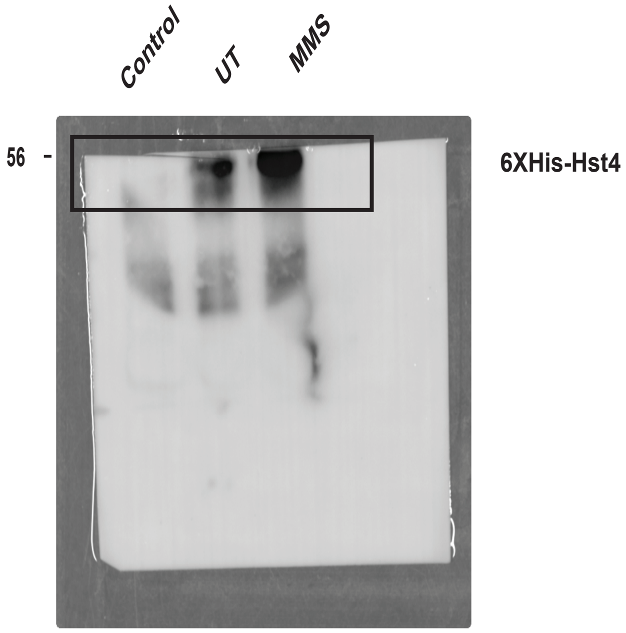

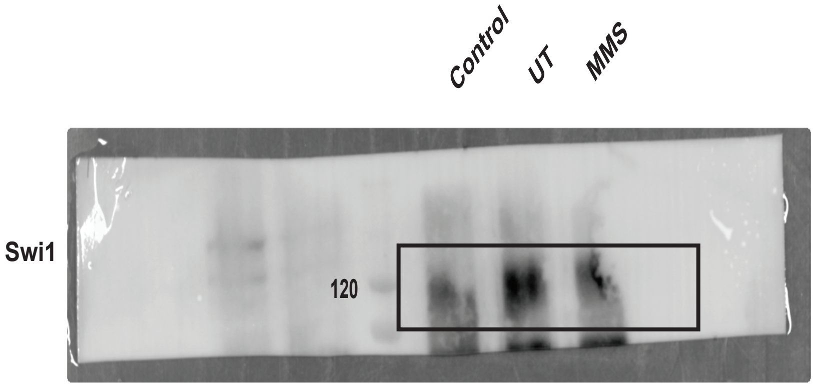

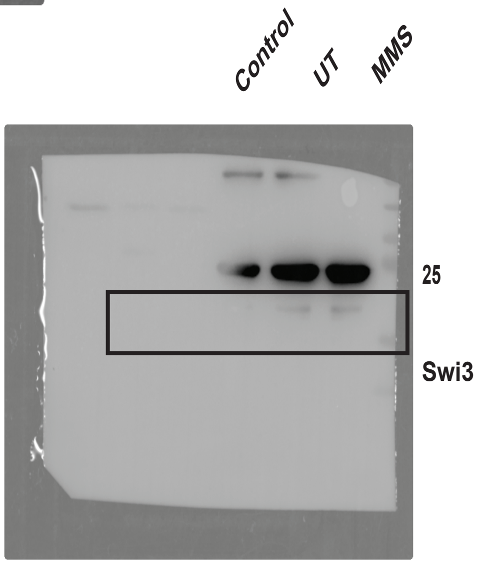

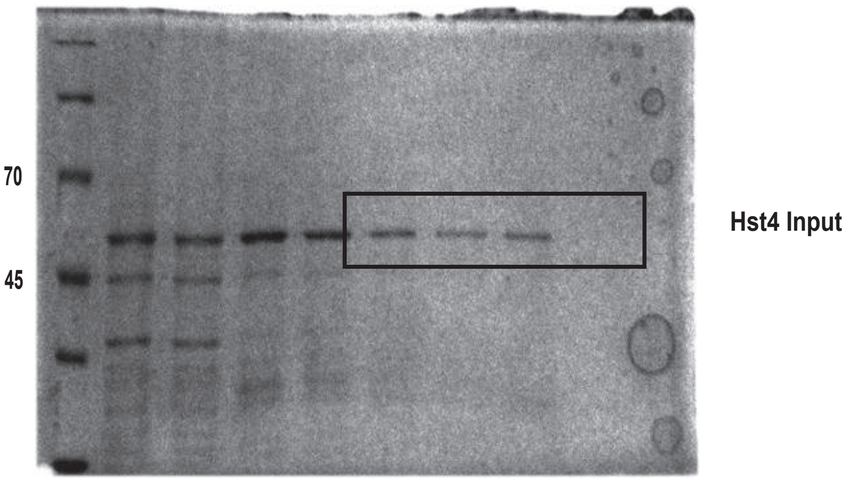

Purification profile of Hst4

Figure 7-source data 1

Figure 7C

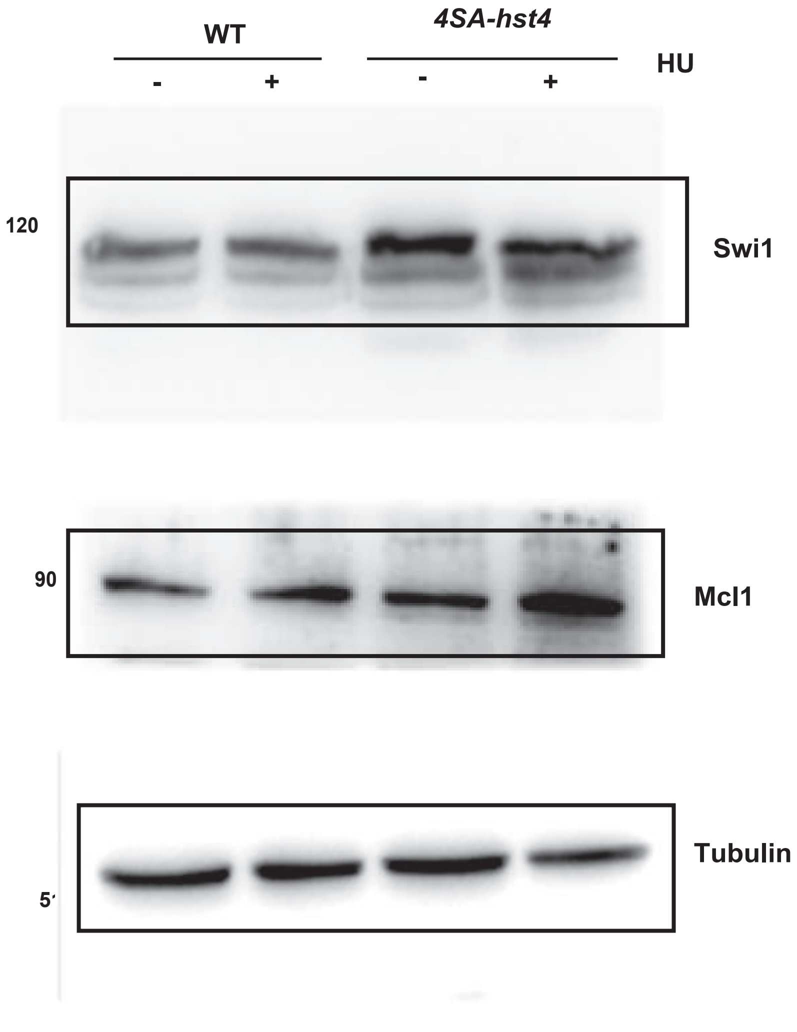

Figure 7-source data 1

Figure 7D

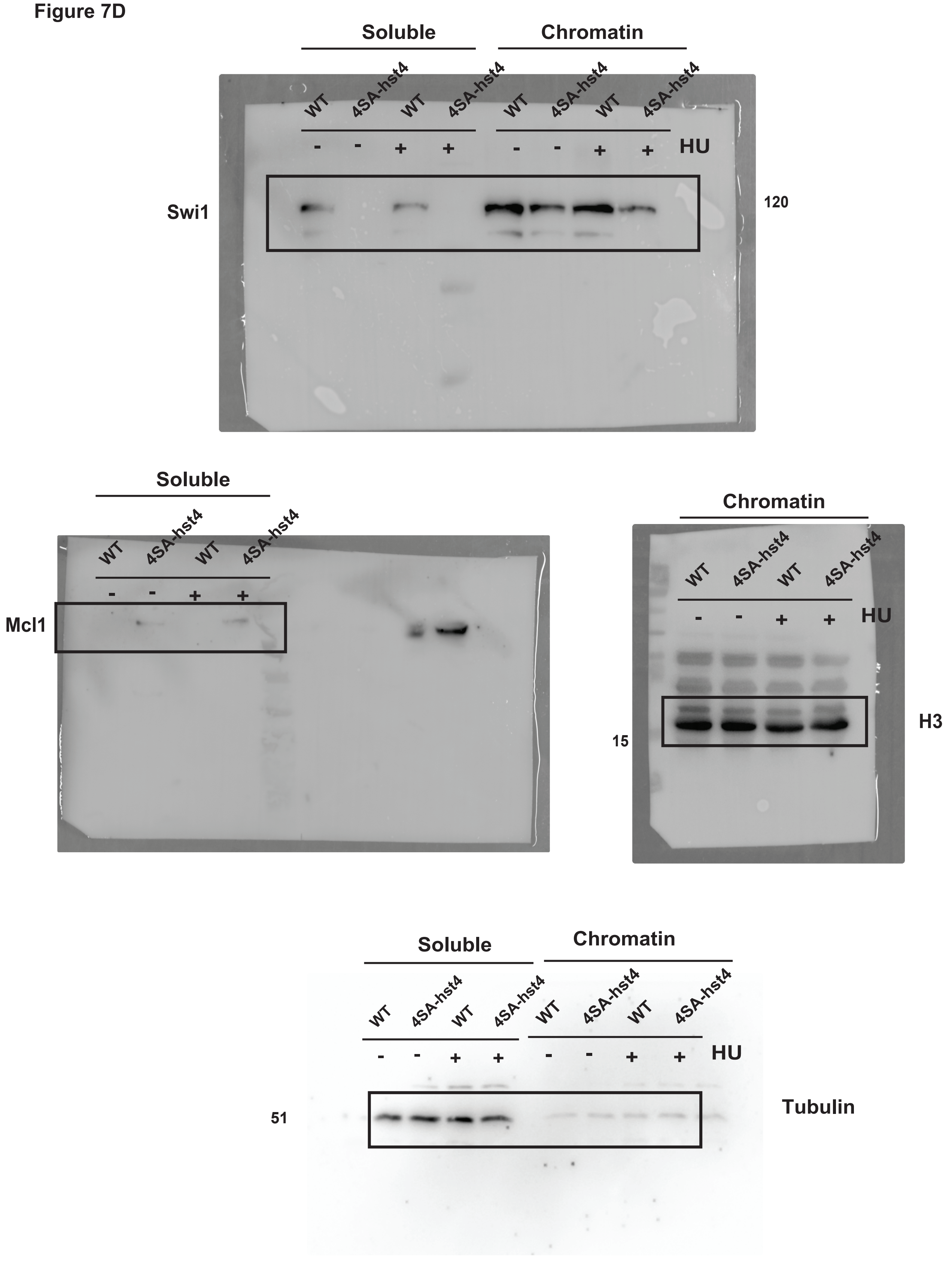

Figure 7-source data 1

Figure 7F

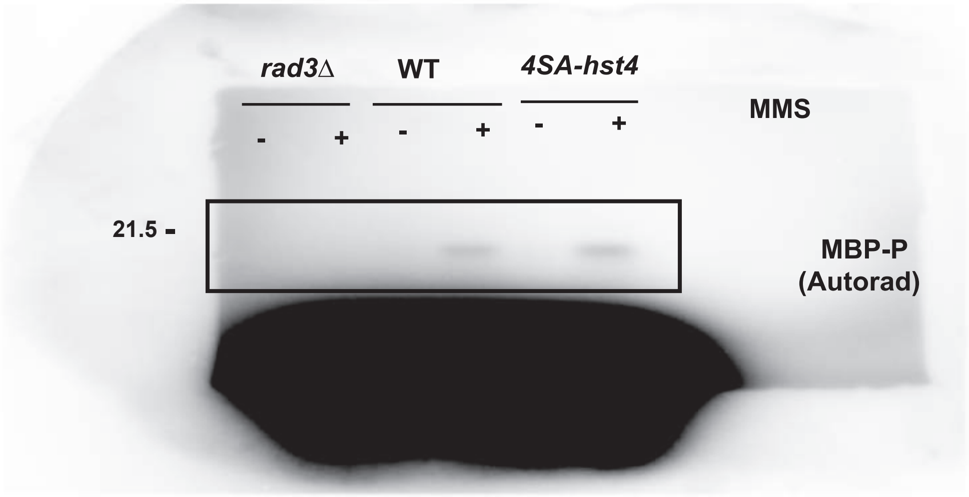

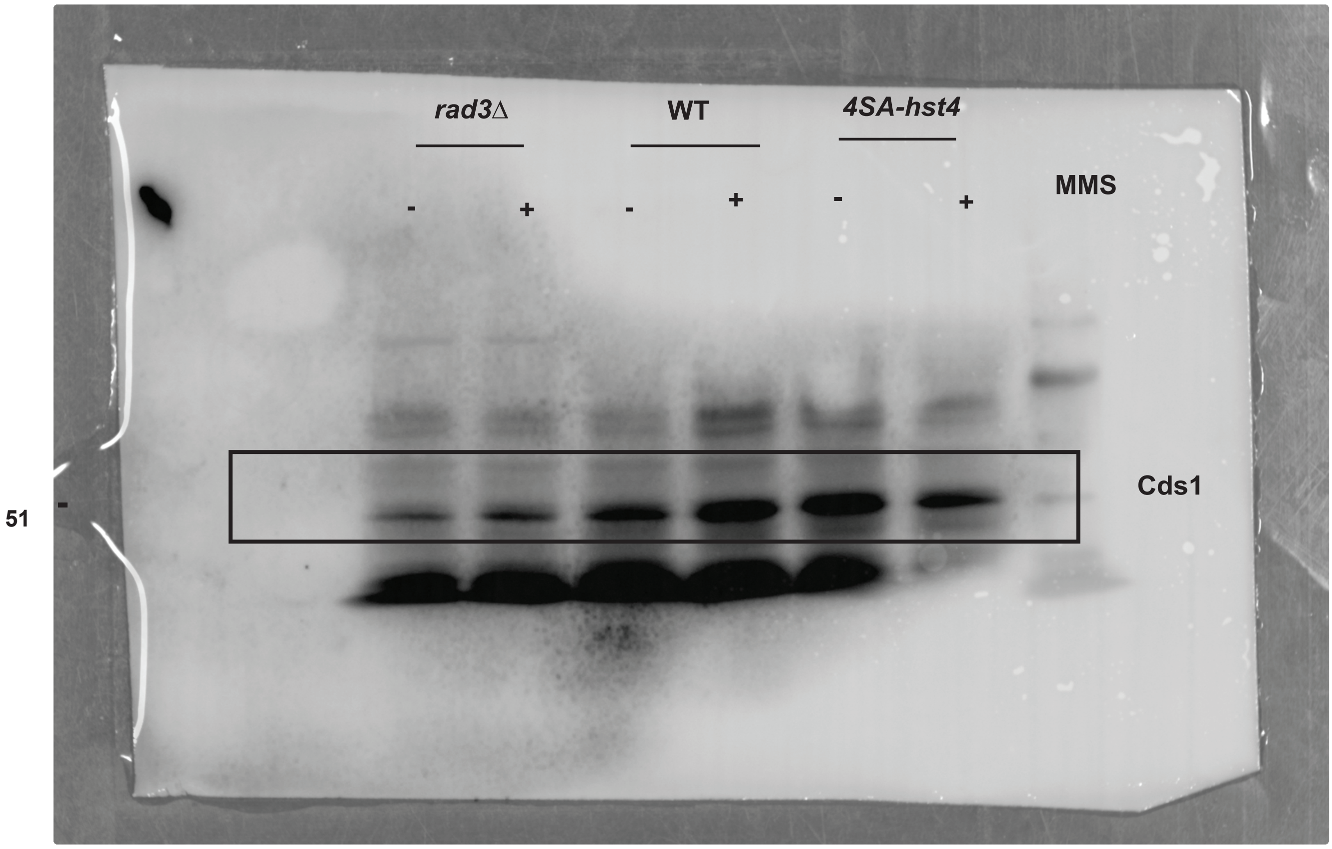

Figure 7-figure supplement 1-source data 1

Figure 7-figure supplement 1D

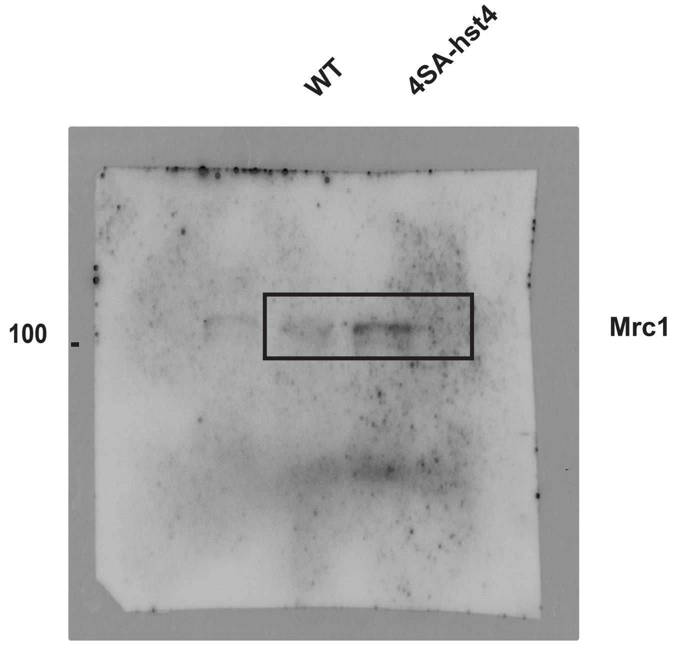

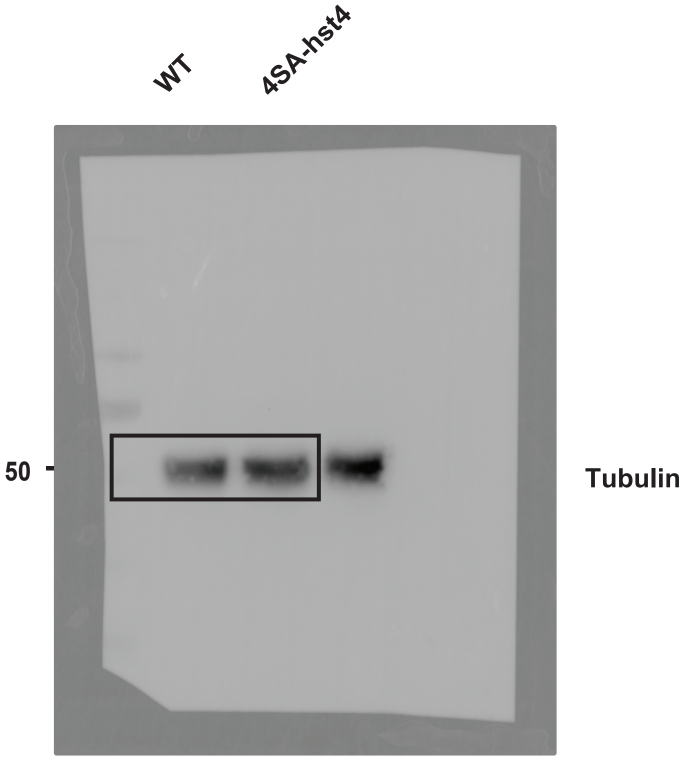

Figure 7-figure supplement 1-source data 1

Figure 7-figure supplement 1E

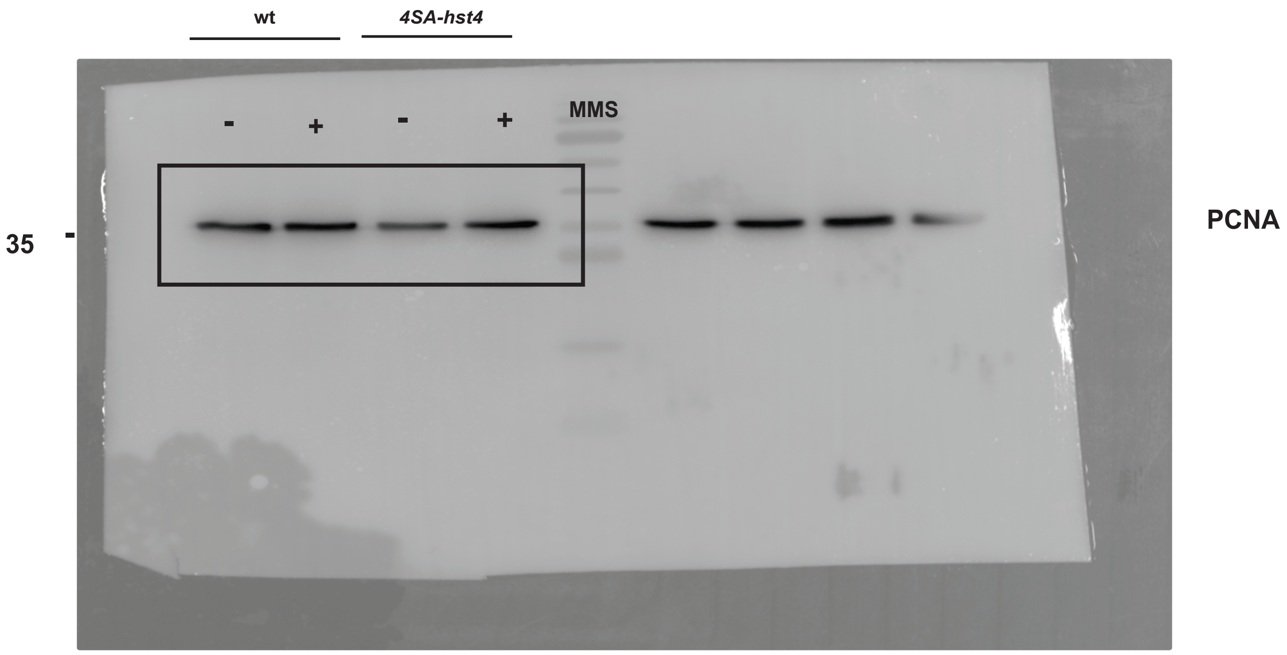

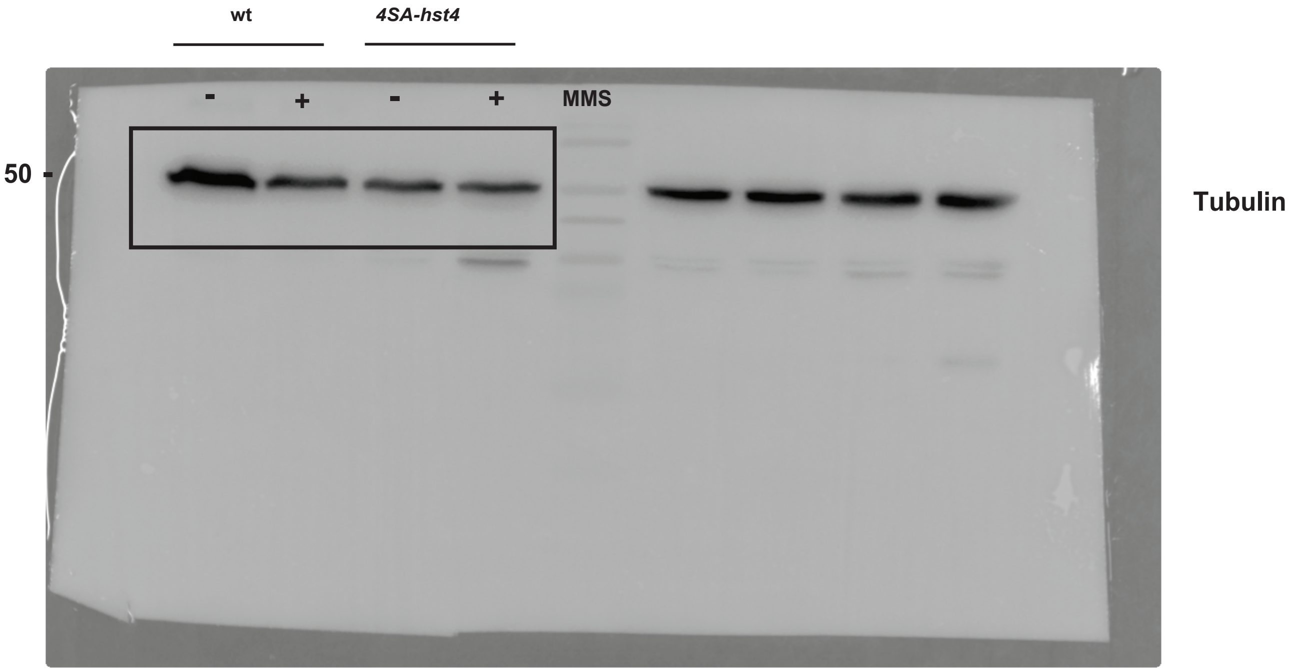

Figure 7-figure supplement 1-source data 1

Figure 7-figure supplement 1F

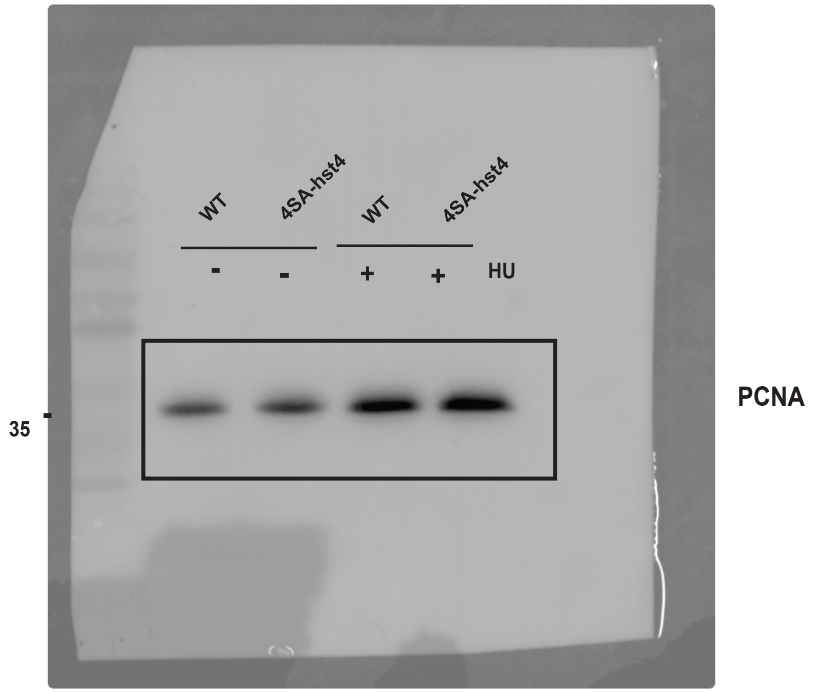

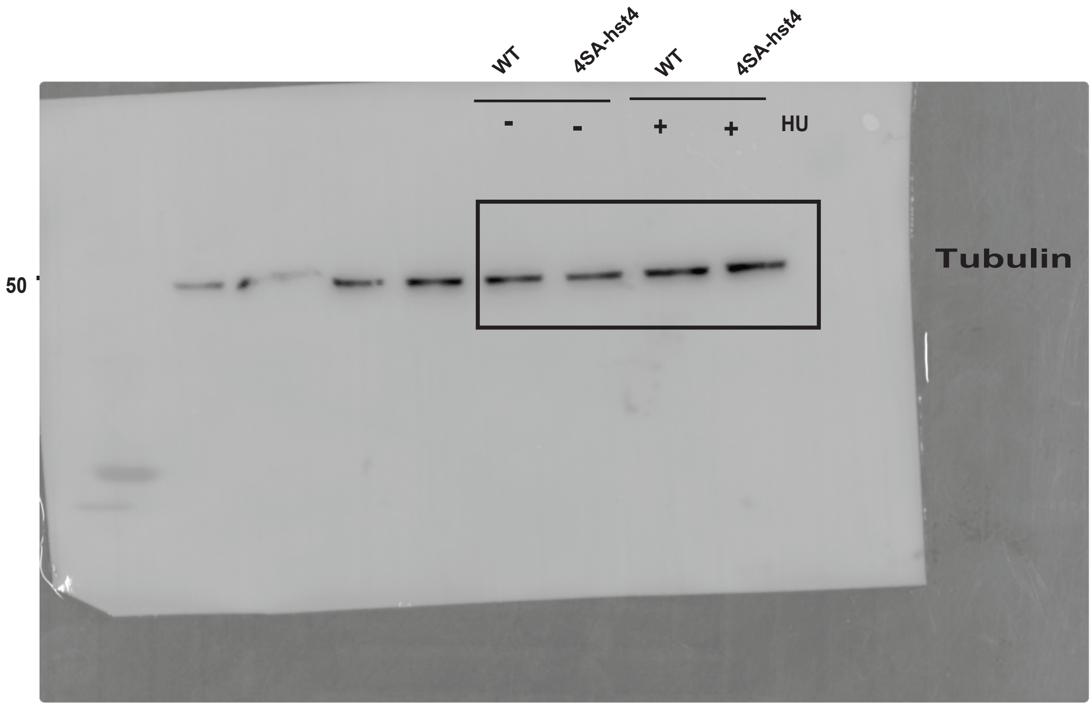

Figure 8-source data 1

Figure 8A

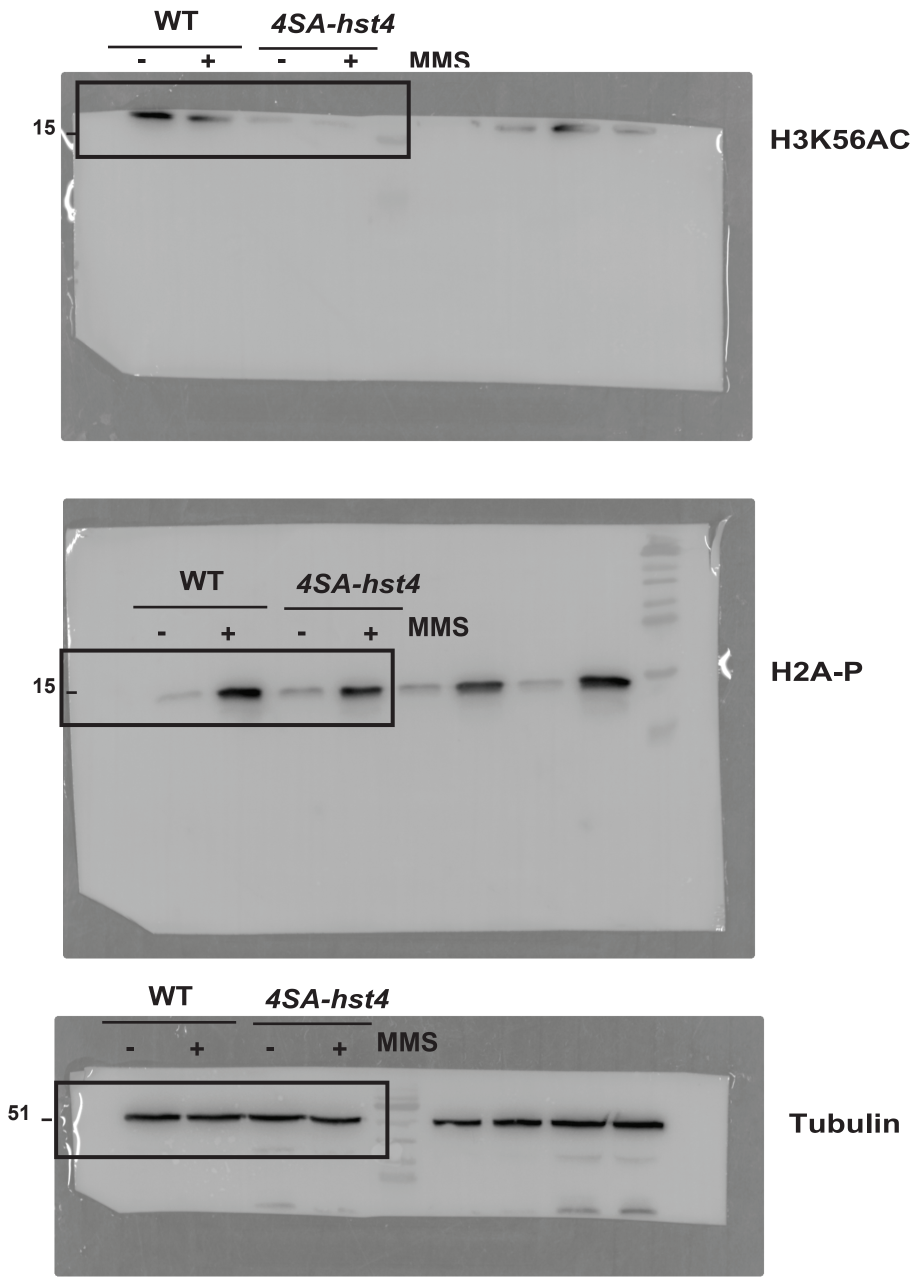

Figure 8-source data 1

Figure 8B

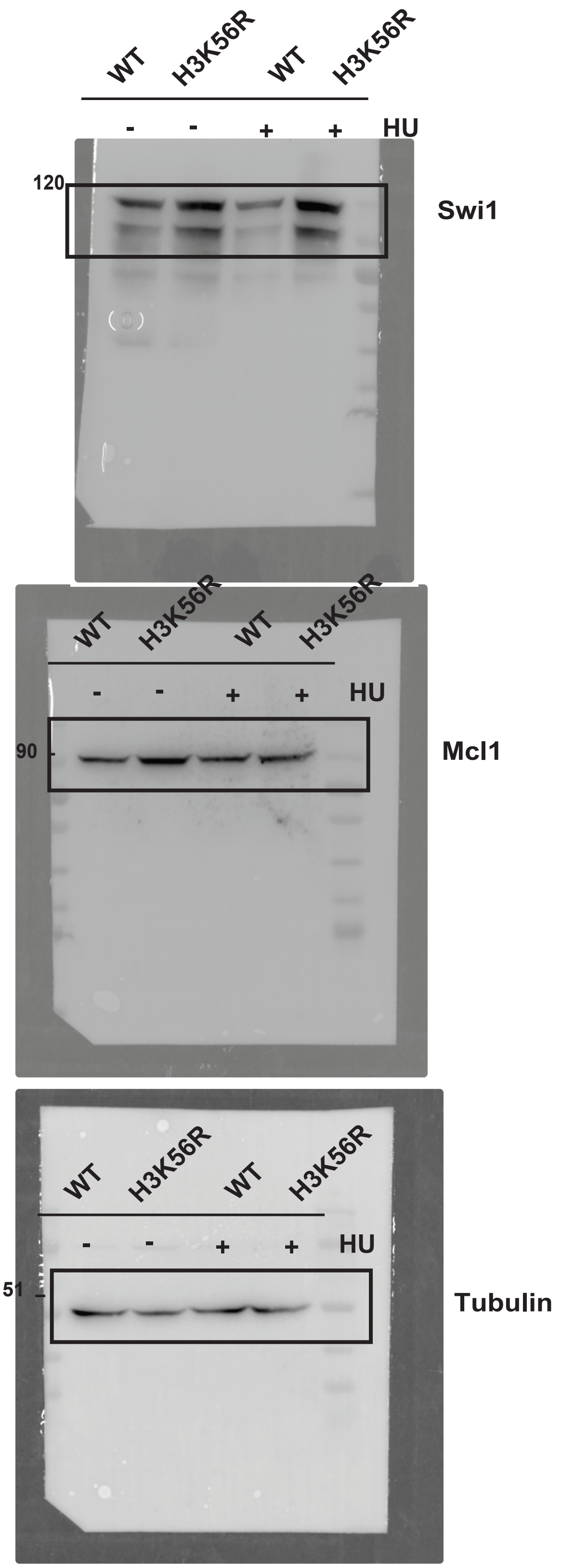

Figure 8-source data 1

Figure 8C

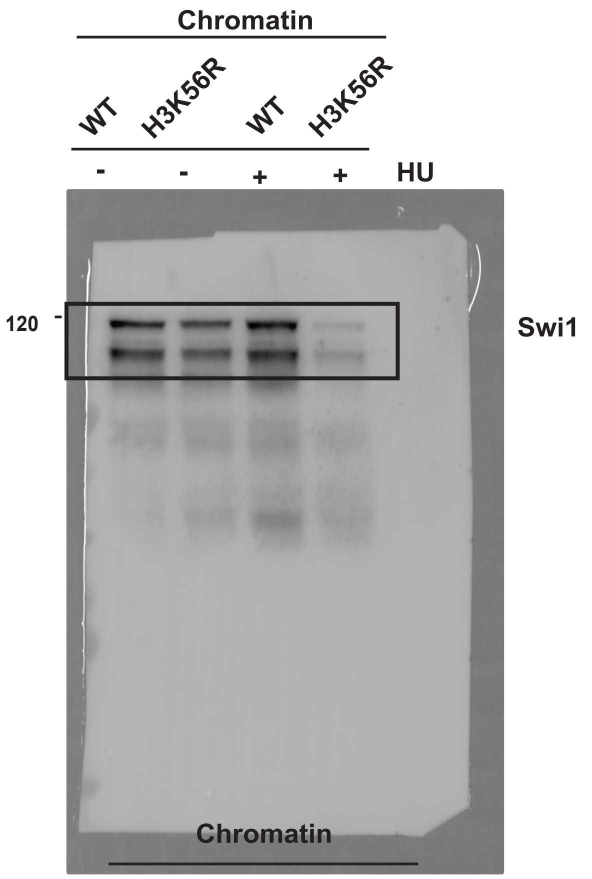

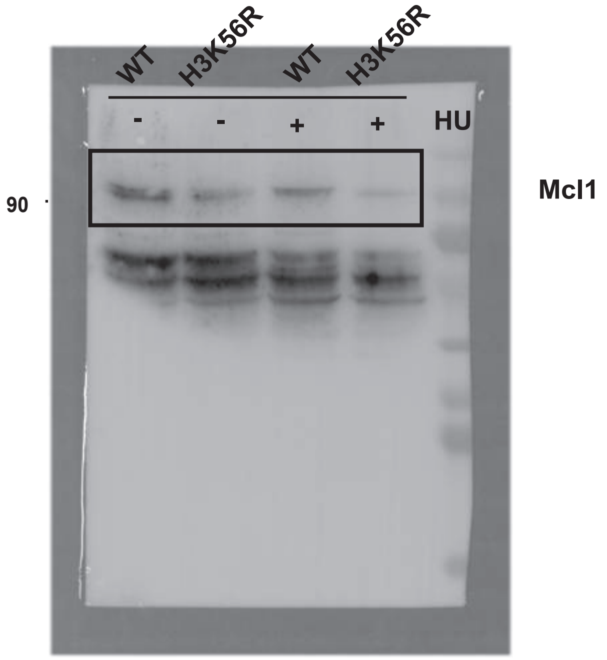

Figure 8-source data 1

Figure 8D

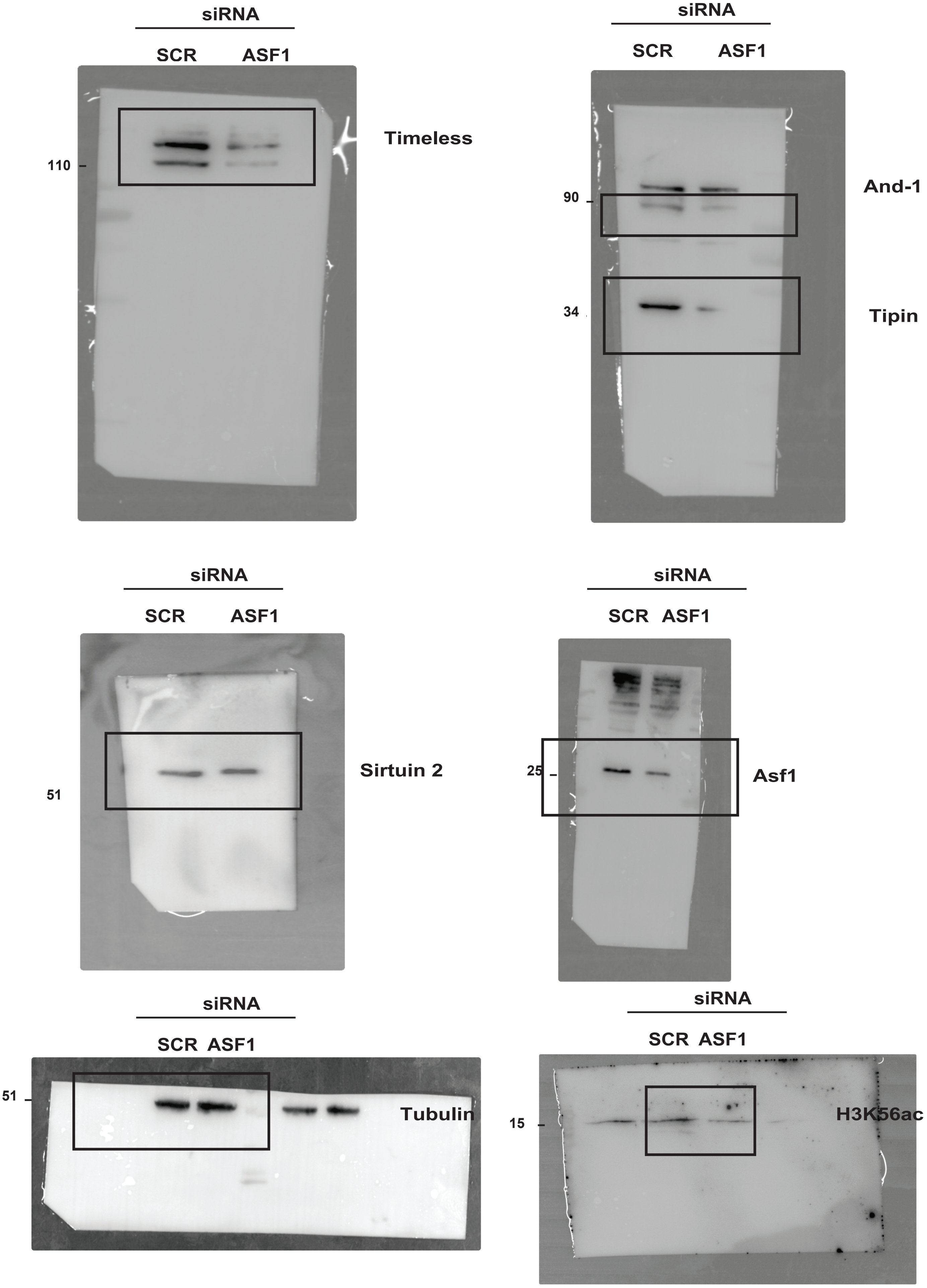

Figure 8-source data 1

Figure 8E

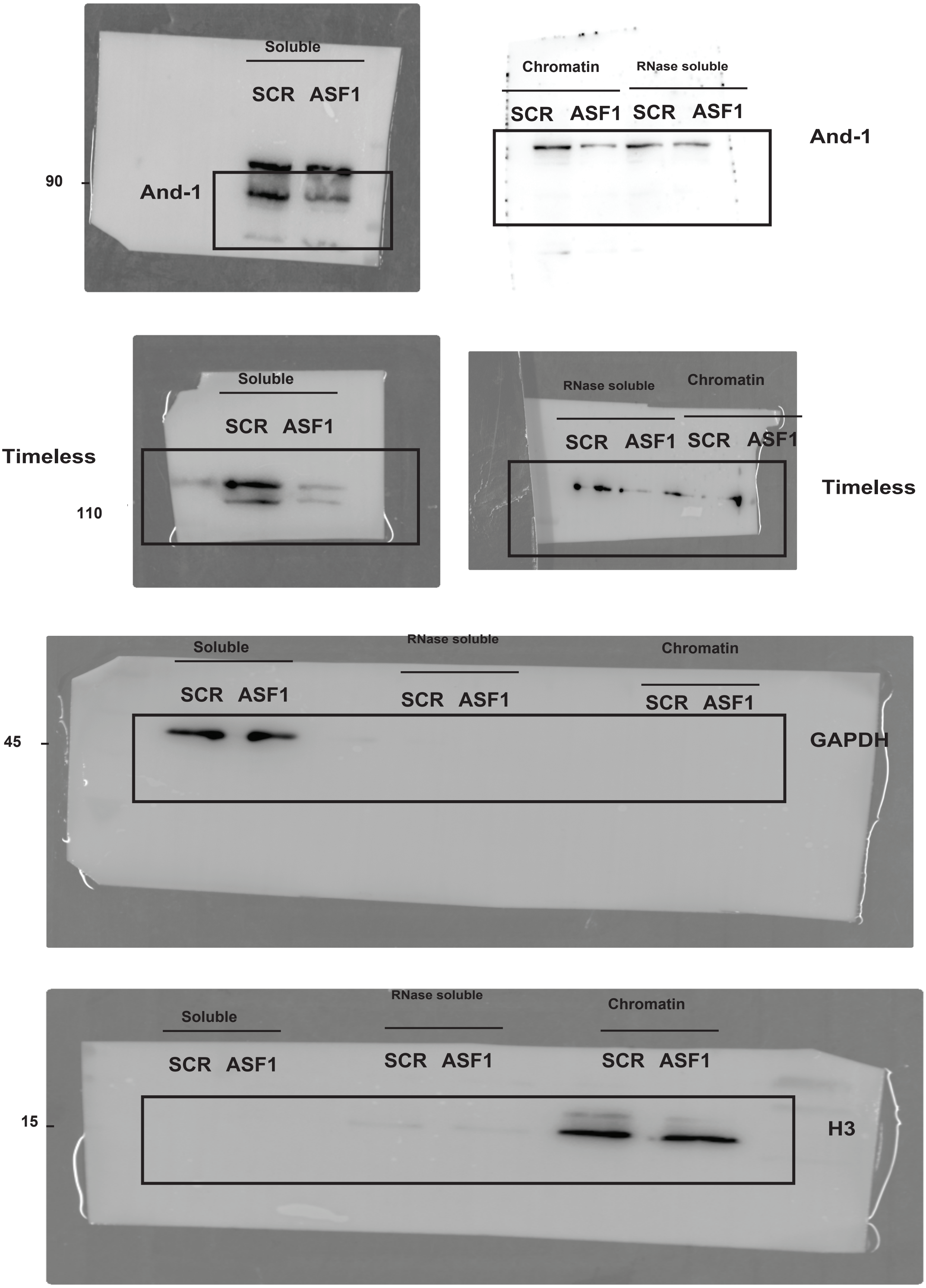

Figure 8-figure supplement 1-source data 1

Figure 8-figure supplement 1A

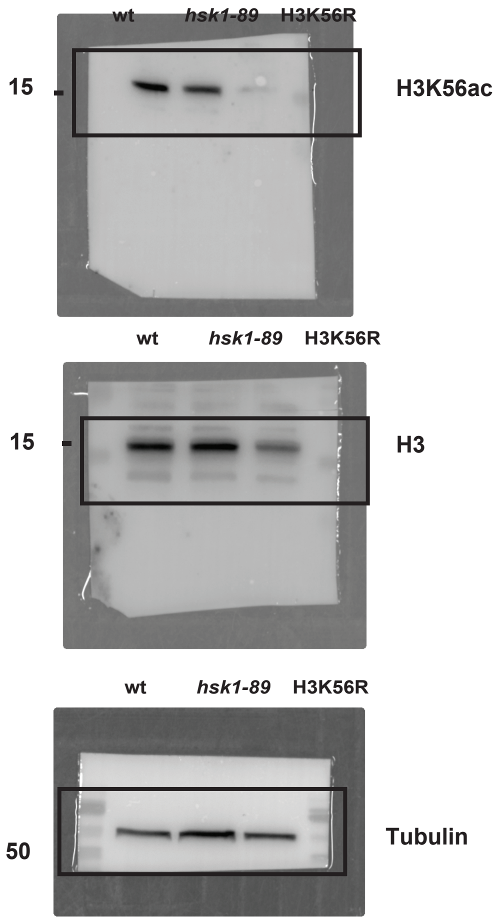

Figure 8-figure supplement 1-source data 1

Figure 8-figure supplement 1B

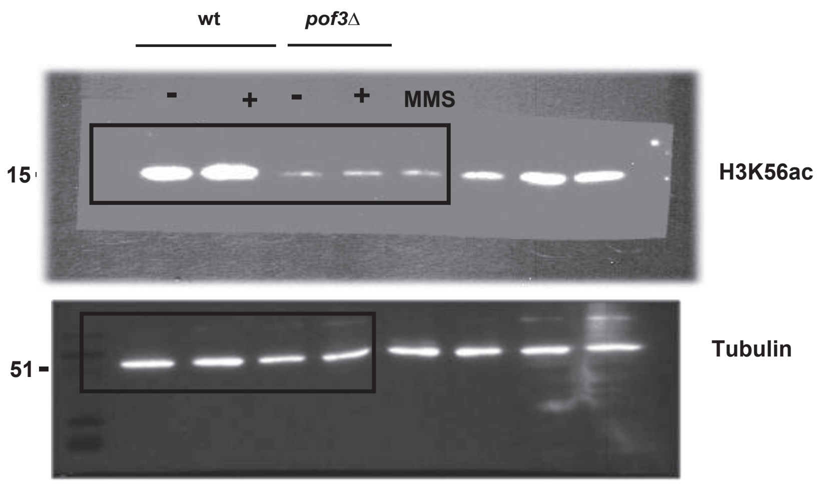

CellSure-Human: Human Cell line Authentication Service

Report

**Project ID:**	**M-1083**
**Service Code:**	**LC-M-321**
**Reference**	**PO No:**
	**Service Order Form Dated: 17/08/2016**
**Date**	**08/09/2016**
**Prepared For**	**Dr. Rashna Bhandari**<br>Laboratory of Cell Signalling, CDFD<br>Laboratory building, Tuljaguda complex,<br>4-1-714, Mozamjahi Road, Nampally<br>Hyderabad-500001
**Prepared By**	**Gaurave Bhatt**
**Reviewed By**	**Jameel Ahamd Khan, PhD**

Lifecode Technologies Private Limited
A5, Mohan Co-operative Industrial Estate, Mathura Road, New Delhi-110 044 (INDIA)
Email: info@lifecode.in | Phone: +91-11-41 306 509

Sample Information

Sample Type	Cells suspended in Transport Buffer
Sample IDs	1. RB-1
Shipping Method	Sample received through courier at room temperature
Receiving Date:	22/08/2016
Report submission date	08-09-2016

Methodology

The total cellular DNA was isolated using our standard protocol. For STR profiling, total ten Genetic loci viz: TH01, D21S11, D5S818, D13S317, D7S820, D16S539, CSF1PO, AMEL, vWA and TPOX were PCR amplified by labelled primers specific to repective loci. The labelled amplicons were resolved by capillary electrophoresis and alleles were called. The sample genotypes were compared with reference STR genotypes available in ATCC®, DSMZ® and Biosample databases to authenticate sample identity and check for cross contamination.

Results

Genotypes:

S. No	Sample ID	Cell Line Name	Ref Db ID		D5S818	D13S317	D7S820	D16S539	vWA	TH01	AMEL	TPOX	CSF1PO	D21S11	% Match with Ref.
**1.**	**RB-1**	U-2 OS	HTB-96	*Ref. Genotype (ATCC & Biosample)*	*11*	*13*	*11, 12*	*11, 12*	*14, 18*	*6, 9.3*	*X*	*11, 12*	*13*	*31*	
				Sample Genotype	*11*	*13*	*11, 12*	*11, 12*	*14, 18*	*6, 9.3*	*X*	*11, 12*	*12, 13*	*31*	***93.75***
**2.**	**Pos Ctrl**	2800M	-	*Ref. Genotype (Promega)*	*12*	*9, 11*	*8, 11*	*9, 13*	*16, 19*	*6, 9.3*	*X, Y*	*11*	*12*	*29, 31.2*	
				Sample Genotype	*12*	*9, 11*	*8, 11*	*9, 13*	*16, 19*	*6, 9.3*	*X, Y*	*11*	*12*	*29, 31.2*	***100***

Lifecode Technologies Private Limited
A5, Mohan Co-operative Industrial Estate, Mathura Road, New Delhi-110 044 (INDIA)
Email: info@lifecode.in | Phone: +91-11-41 306 509 | Web: www.lifecode.in

Comments:

1. **RB-1:** The STR marker profile of the sample matches with **93.75 % markers** of reference marker profile of U-2 OS (ATCC ID: HTB-96) cell line in NCBI-Biosample and **93.33%** markers in ATCC database (please also see additional data). According to ATCC guidelines >80 % match **may be considered to be authenticated.**

   However, it should be noted that the reason for not getting perfect (100 %) match is because of loss and/or gain of alleles at loci: CSF1PO (please see the highlighted markers in above table). This could be either because of sample from very high passage number or clonally selected cells, from a cell line of inherently very unstable genome. **For such cell lines, the STR profiling at regular intervals to check whether the current STR signature is maintained with increase in passage number of cells, is highly recommended.**

   However, it may also be a potential case of cros-contamination and/or mislabelling and should be checked at a later passage especially if another cell line with related STR marker profile (please see DSMZ database serch results in additional information) is being cultured in same lab. It may also be a laboratory developed cell line from patient samples which is not yet submitted in ATCC, DSMZ, JCRB and RIKEN databases and requires further charecterization.

Date: 08-09-2016 Jameel A. Khan, PhD

NOTE: THIS REPORT IS FOR RESEARCH PURPOSES ONLY. IT CAN NOT BE USED FOR TECHNO-LEGAL OR ANY OTHER PURPOSES.

Additional Data

Chromatograms

Allelic Ladder

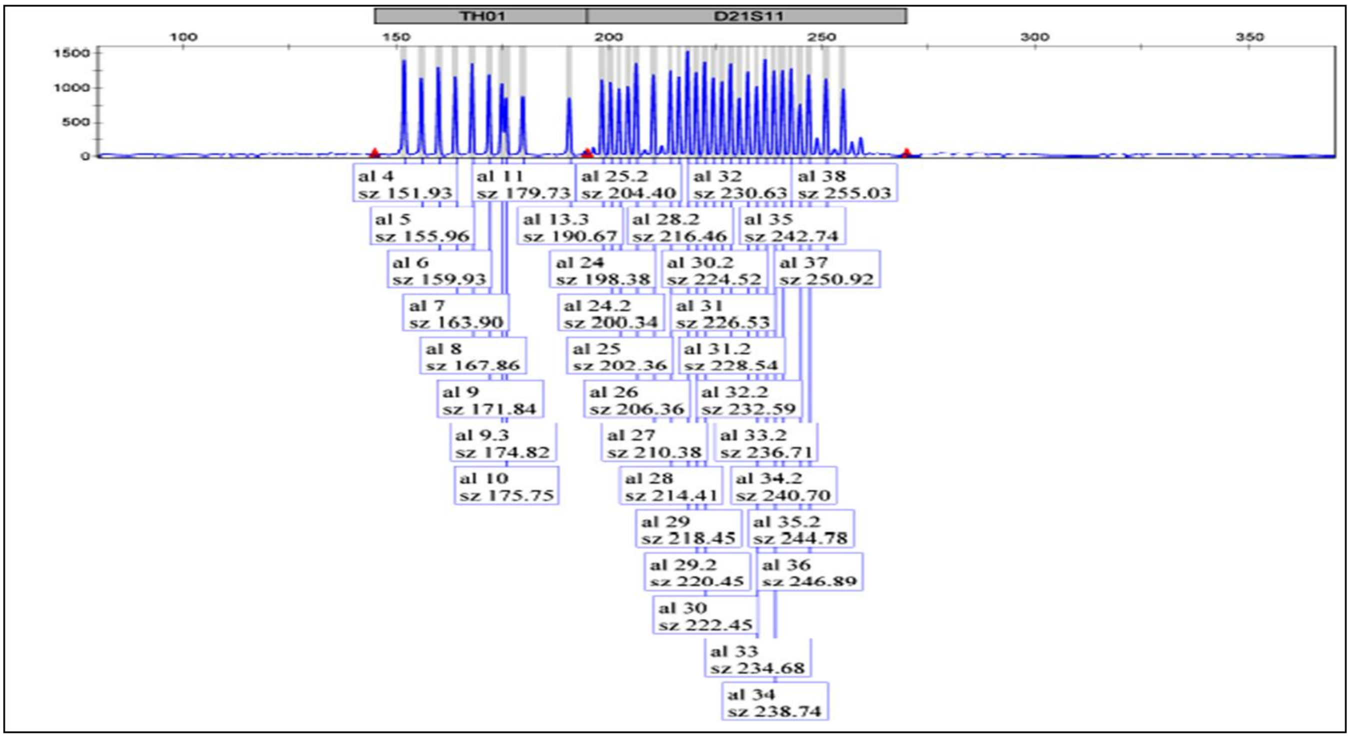

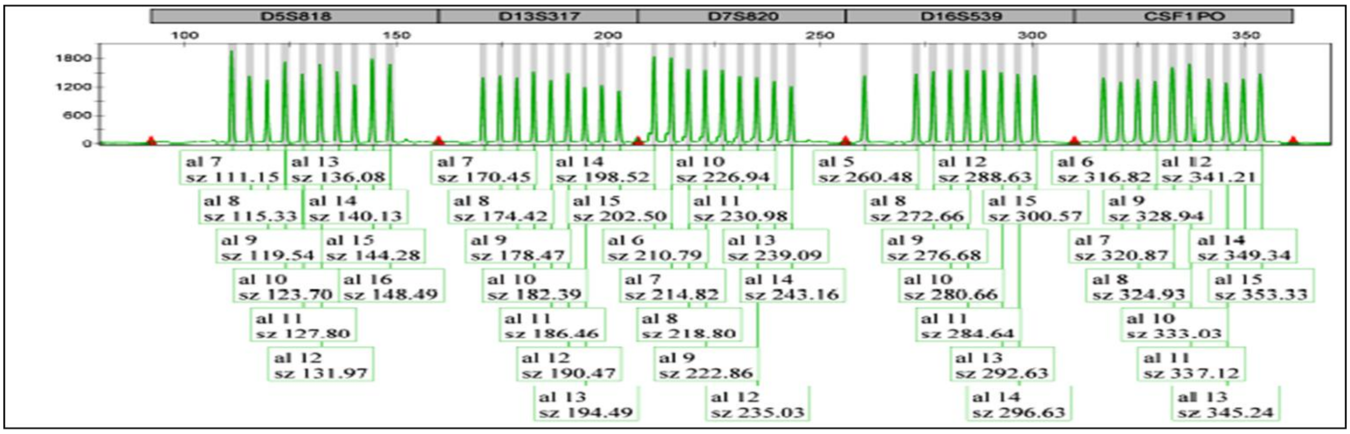

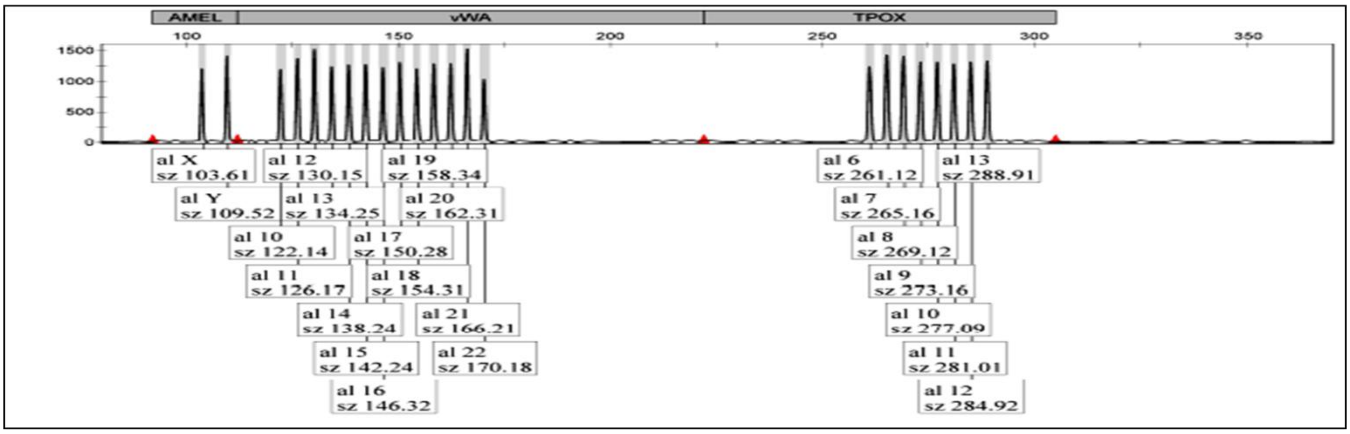

1- RB-1

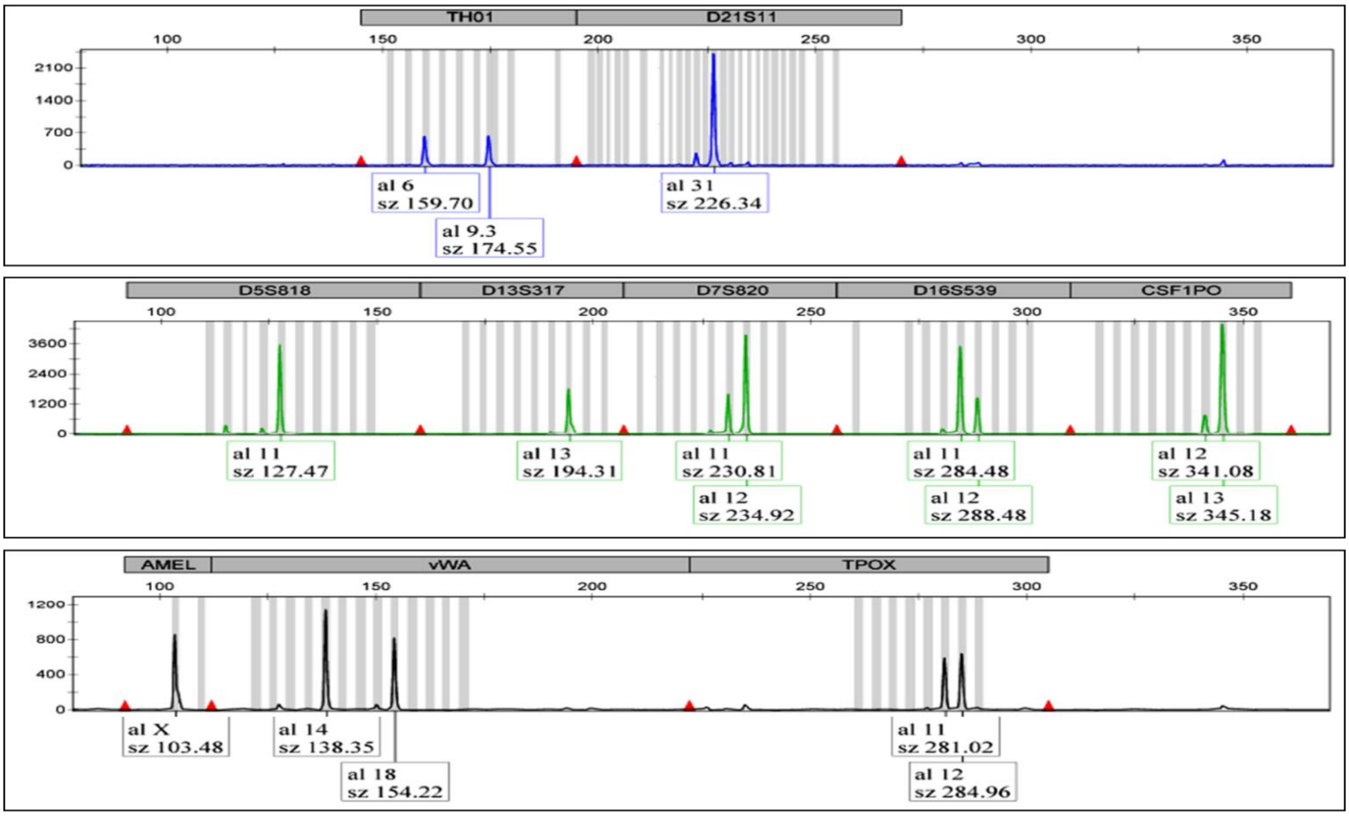

2- Positive Ctrl

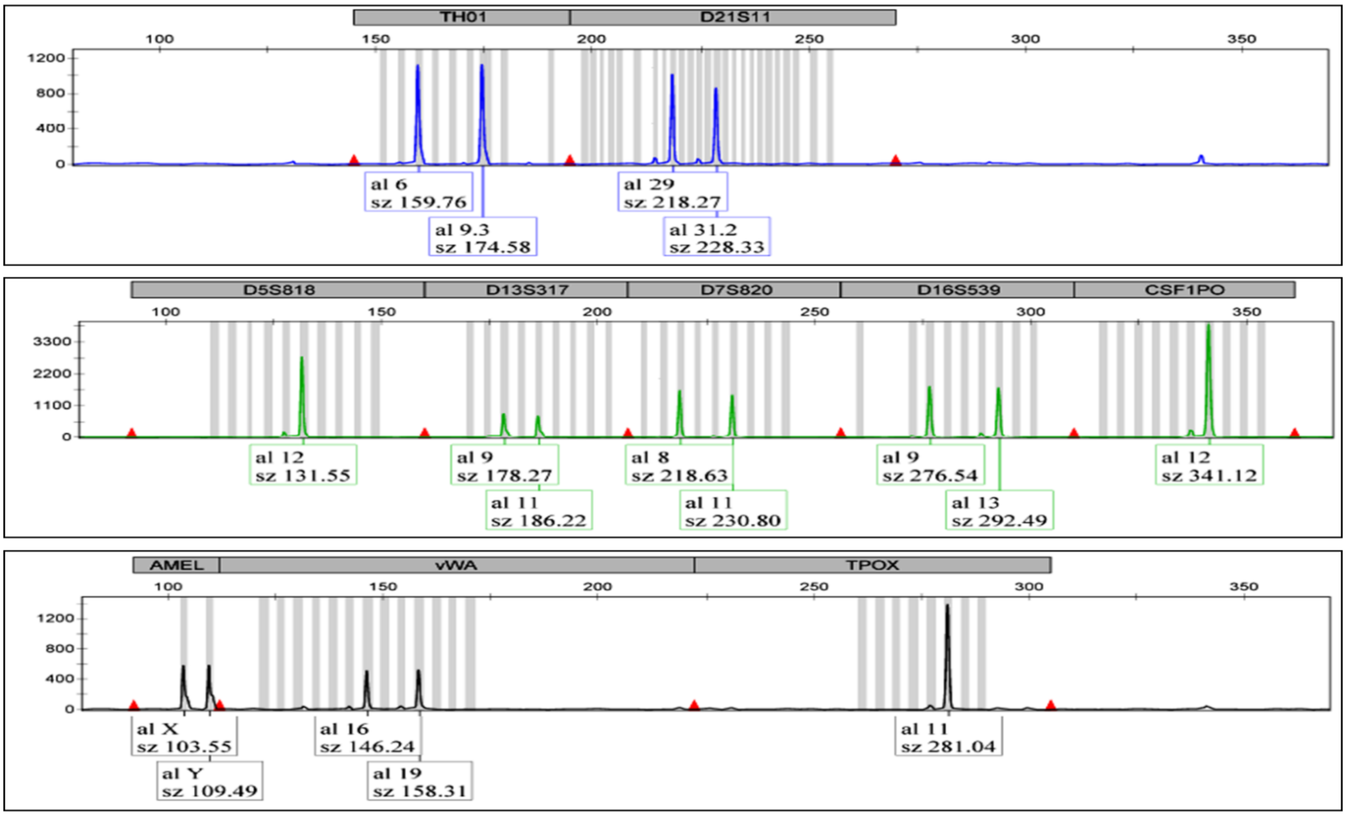

Lifecode Technologies Private Limited
A5, Mohan Co-operative Industrial Estate, Mathura Road, New Delhi-110 044 (INDIA)
Email: info@lifecode.in | Phone: +91-11-41 306 509 | Web: www.lifecode.in

Database Search Results: ATCC

1- RB-1

S. No.	% Match	No of Matches	ATCC Number	Designation	D5S818	D13S317	D7S820	D16S539	vWA	TH01	AMEL	TPOX	CSF1PO
			**RB-1 (sample genotype)**		***11***	***13***	***11, 12***	***11, 12***	***14, 18***	***6, 9.3***	***X***	***11, 12***	***12, 13***
**1**	93.33	14	HTB-96	U-2 OS	*11*	*13*	*11, 12*	*11, 12*	*14, 18*	*6, 9.3*	*X*	*11, 12*	*13*
**2**	93.75	15	785	U-2-OS	*8, 11*	*13*	*11, 12*	*11, 12*	*14, 18*	*6, 9.3*	*X*	*11, 12*	*12, 13*

Lifecode Technologies Private Limited
A5, Mohan Co-operative Industrial Estate, Mathura Road, New Delhi-110 044 (INDIA)
Email: info@lifecode.in | Phone: +91-11-41 306 509 | Web: www.lifecode.in

Database Search Results: DSMZ (top 30 hits)

1- RB-1

S. No.	EV (Expected Value)	Cell No.	Designation	D5S818	D13S317	D7S820	D16S539	vWA	TH01	AMEL	TPOX	CSF1PO
		**RB-1 (sample genotype)**		***11***	***13***	***11, 12***	***11, 12***	***14, 18***	***6, 9.3***	***X***	***11, 12***	***12, 13***
**1**	0.94(34/36)	785	U-2-OS	*8, 11*	*13, 13*	*11, 12*	*11, 12*	*14, 18*	*6, 9.3*	*X, X*	*11, 12*	*12, 13*
**2**	0.94(34/36)	HTB-96	U-2 OS	*11, 11*	*13, 13*	*11, 12*	*11, 12*	*14, 18*	*6, 9.3*	*X, X*	*11, 12*	*13, 13*
**3**	0.67(24/36)	326	SW-948	*11, 11*	*10, 11*	*9, 11*	*11, 12*	*16, 18*	*6, 9.3*	*X, X*	*8, 11*	*12, 12*
**4**	0.67(24/36)	CCL-237	SW948 [SW-948]	*11, 11*	*10, 11*	*9, 11*	*11, 12*	*16, 18*	*6, 9.3*	*X, X*	*8, 11*	*12, 12*
**5**	0.67(24/36)	CRL-5975	UMC-11	*11, 11*	*8, 13*	*9, 11*	*11, 12*	*14, 15*	*6, 9.3*	*X, Y*	*9, 11*	*12, 12*
**6**	0.67(24/36)	CRL-7242	Hs 329.T	*11, 13*	*9, 11*	*11, 12*	*11, 12*	*17, 18*	*6, 9.3*	*X, X*	*8, 11*	*11, 12*
**7**	0.67(24/36)	JCRB1020	RERF-LC-Ad1	*12, 12*	*13, 13*	*11, 12*	*9, 12*	*14, 17*	*6, 9*	*X, X*	*8, 11*	*12, 13*
**8**	0.67(24/36)	RCB2107	TN-1	*10, 11*	*8, 13*	*11, 12*	*11, 12*	*14, 19*	*6, 6*	*X, X*	*8, 11*	*11, 12*
**9**	0.67(24/36)	RCB2260	HE41	*11, 11*	*8, 11*	*11, 11*	*9, 12*	*14, 18*	*6, 7*	*X, X*	*8, 11*	*12, 13*
**10**	0.65(24/37)	RCB1021	T3M-4	*11, 11*	*10, 13*	*12, 12*	*11, 12*	*18, 19, 21*	*9.3, 9.3*	*X, X*	*11, 12*	*10, 10*
**11**	0.65(24/37)	RCB1426	PMF-ko14	*11, 11*	*13, 14, 15*	*8, 8*	*11, 12*	*17, 18*	*9.3, 9.3*	*X, X*	*11, 12*	*10, 12*
**12**	0.63(24/38)	665	UPCI-SCC-099	*11, 12*	*8, 13*	*7, 9, 10*	*11, 12*	*14, 18, 19*	*6, 9.3*	*X, X*	*11, 11*	*11, 12*
**13**	0.61(22/36)	182	COLO-680N	*11, 11*	*13, 13*	*10, 12*	*11, 12*	*17, 18*	*8, 8*	*X, X*	*6, 8*	*11, 12*
**14**	0.61(22/36)	185	LCL-HO	*11, 11*	*13, 13*	*8, 12*	*13, 13*	*14, 18*	*7, 9.3*	*X, X*	*9, 11*	*9, 11*

Lifecode Technologies Private Limited
A5, Mohan Co-operative Industrial Estate, Mathura Road, New Delhi-110 044 (INDIA)
Email: info@lifecode.in | Phone: +91-11-41 306 509 | Web: www.lifecode.in

**15**	0.61(22/36)	255	CADO-ES1	*11, 12*	*10, 13*	*11, 13*	*9, 11*	*14, 18*	*6, 9*	*X, X*	*8, 11*	*11, 12*
**16**	0.61(22/36)	294	SW-403	*11, 11*	*13, 13*	*8, 9*	*10, 12*	*14, 18*	*6, 6*	*X, X*	*8, 9*	*10, 13*
**17**	0.61(22/36)	557	ROS-50	*11, 11*	*8, 13*	*10, 11*	*8, 12*	*17, 18*	*6, 9.3*	*X, Y*	*11, 11*	*11, 12*
**18**	0.61(22/36)	721	NCEB-1	*9, 11*	*12, 12*	*10, 11*	*9, 10*	*14, 18*	*6, 9.3*	*X, X*	*11, 11*	*12, 13*
**19**	0.61(22/36)	CCL-230	SW403 [SW-403]	*11, 11*	*13, 13*	*8, 9*	*10, 12*	*14, 18*	*6, 6*	*X, X*	*8, 9*	*10, 13*
**20**	0.61(22/36)	CRL-1635	Hs68	*8, 11*	*11, 13*	*11, 12*	*11, 12*	*16, 16*	*6, 9.3*	*X, Y*	*11, 11*	*9, 12*
**21**	0.61(22/36)	CRL-2555	Panc 04.03	*11, 11*	*11, 12*	*8, 12*	*11, 12*	*14, 16*	*9.3, 9.3*	*X, X*	*10, 11*	*12, 12*
**22**	0.61(22/36)	CRL-3005	NCEB-1	*9, 11*	*12, 12*	*10, 11*	*9, 10*	*14, 18*	*6, 9.3*	*X, X*	*11, 11*	*12, 13*
**23**	0.61(22/36)	CRL-7193	Hs 228.T	*11, 12*	*8, 9*	*10, 11*	*11, 12*	*14, 18*	*8, 9.3*	*X, X*	*8, 11*	*11, 12*
**24**	0.61(22/36)	HTB-107	SW579 [SW 579, SW-579]	*11, 11*	*13, 13*	*8, 9*	*11, 11*	*14, 18*	*8, 9.3*	*X, X*	*8, 10*	*13, 13*
**25**	0.61(22/36)	IFO50350	Hs68	*8, 11*	*11, 13*	*11, 12*	*11, 12*	*16, 16*	*6, 9.3*	*X, Y*	*11, 11*	*9, 12*
**26**	0.61(22/36)	IFO50436	KS-1	*8, 12*	*11, 13*	*11, 11*	*11, 12*	*14, 18*	*6, 9*	*X, X*	*8, 11*	*10, 12*
**27**	0.61(22/36)	RCB0210	HF19	*11, 12*	*9, 13*	*11, 12*	*13, 14*	*14, 18*	*6, 7*	*X, X*	*8, 11*	*10, 13*
**28**	0.61(22/36)	RCB2352	633	*11, 11*	*11, 11*	*8, 11*	*11, 12*	*14, 14*	*8, 9.3*	*X, X*	*8, 11*	*10, 12*
**29**	0.59(22/37)	JCRB0183	QGP-1	*12, 12*	*13, 13*	*12, 12*	*10, 12*	*14, 17, 18*	*6, 9*	*X, X*	*8, 11*	*10, 12*
**30**	0.56(20/36)	41	LP-1	*11, 11*	*12, 12*	*11, 12*	*11, 12*	*17, 17*	*7, 8*	*X, X*	*11, 11*	*11, 12*

Lifecode Technologies Private Limited
A5, Mohan Co-operative Industrial Estate, Mathura Road, New Delhi-110 044 (INDIA)
Email: info@lifecode.in | Phone: +91-11-41 306 509 | Web: www.lifecode.in
